# Toxicological Effects of Air Pollutants on Human Airway Cell Models Using Air–liquid Interface Systems: A Systematic Review

**DOI:** 10.1007/s40572-025-00491-w

**Published:** 2025-07-28

**Authors:** Óscar Navarrete-Aliaga, María Muriach, Juana Maria Delgado-Saborit

**Affiliations:** 1 Department of Medicine, Faculty of Health Sciences, Universitat Jaume I, Avenida de Vicent Sos Baynat S/N, 12071 Castellón de La Plana, Spain; 2 Epidemiology and Environmental Health Joint Research Unit, Foundation for the Promotion of Health and Biomedical Research in the Valencian Region, FISABIO-Public Health, FISABIO–Universitat Jaume I–Universitat de València, Av. Catalunya 21, 46020 Valencia, Spain

**Keywords:** Air pollution, Aerosols, Human health, Toxicity, Oxidative stress, Cellular inflammation, Air–liquid interface

## Abstract

**Purpose of Review:**

Global air pollution has increased significantly in recent decades mainly due to anthropogenic emissions. This results in elevated concentrations of some airborne pollutants like nitrogen dioxide, ozone, volatile organic compounds (VOCs), and particulate matter (PM). In this review, we aim to provide an overview of the current state of knowledge on the toxicological effects of air pollution on airway epithelial cells, the first point of contact of the air pollutants with the body, using air–liquid interface (ALI) models.

**Recent Findings:**

Research on the health effects of air pollution has advanced through studies that take a multidisciplinary approach integrating toxicology, epidemiology, and molecular and cell biology. Submerged cell cultures have been used in most studies for the assessment of air pollution toxicity in vitro, but these show some important limitations. Thus, human airway cellular models based on ALI systems have emerged as very promising approaches in respiratory toxicology due to their closer resemblance to in vivo conditions. Results from 53 studies indicate that both, acute and prolonged exposures to air pollution induce oxidative, inflammatory, and genotoxic responses in airway epithelial cells. The changes in several biomarkers and genes related to the observed health effects were discussed through key molecular pathways, and particularly those related to the oxidative stress state. Lastly, we identified perspectives for future research in this field, such as the use of more complex test (e.g., photochemical ageing) atmospheres and exposure models that are reliable for long-term and repeated exposures.

**Summary:**

This review highlights the role of ALI cellular models as essential tools in respiratory toxicology and environmental health research, providing insights into the molecular mechanisms triggered by air pollution exposure.

**Supplementary Information:**

q1The online version contains supplementary material available at 10.1007/s40572-025-00491-w.

## Introduction

**Fig. 1:**
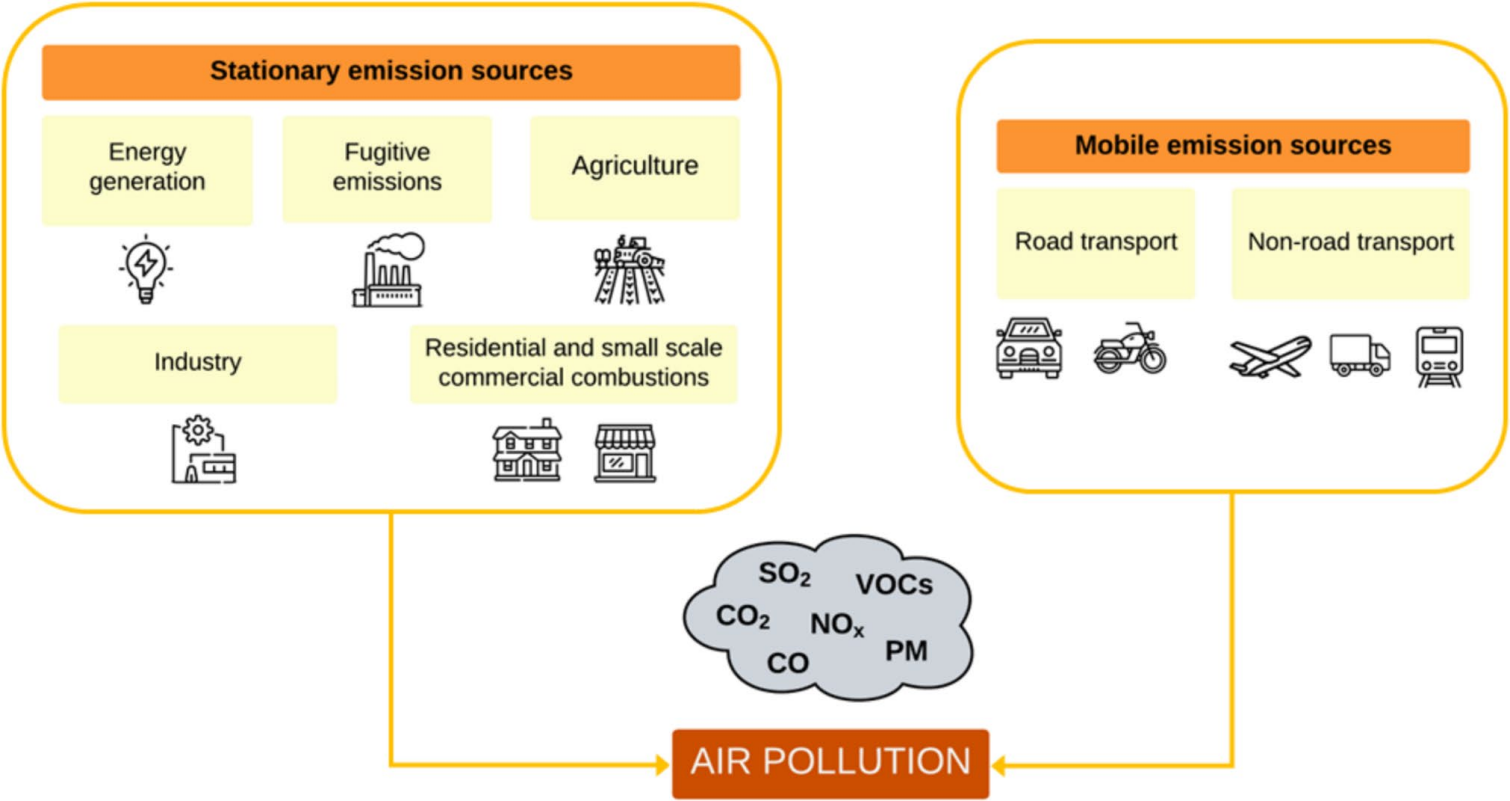
Traditional air pollutants and main emission sources of air pollution. Based on EAA Monograph of Assessment and Management of Urban Air Quality in Europe

Industrialization and population growth are contributing significantly to an increase in global air pollution in recent decades [1], mainly attributed to stationary emission sources, such as industrial activity [2], fugitive emissions [3], power generation facilities [4], agriculture [5] and residential emissions [6, 7] (Fig. 1). Additionally, mobile emissions have become the most important emission source in many areas, with both road [8] and non-road [9] transport making significant contributions and having a significant impact on public health [10, 11].

Some key gaseous pollutants resulting from these activities include nitrogen dioxide (NO_2_) and Volatile Organic Compounds (VOCs), both also contributing to an increase in tropospheric ozone (O_3_) concentrations [12]. Incomplete combustion of organic matter and road traffic emissions are both significant sources of carbon monoxide (CO), while sulphur dioxide (SO_2_) is a by-product of fossil fuel combustion [13]. Particulate matter (PM) is another critical constituent of air pollution, comprising a complex mixture of inhalable airborne particles of varying chemical compositions and sizes. PM is characterized by their aerodynamic diameter, with PM_2.5_ (diameter < 2.5 µm), PM_10_ (diameter < 10 µm) and ultrafine particles (UFP) fraction (diameter < 0.1 µm) being the most common classifications particularly relevant for human health [14–18].

Research on the health effects of air pollution has advanced through population, in vivo and in vitro studies that take a multidisciplinary approach, integrating toxicology, epidemiology, and molecular and cell biology [19].

According to the World Health Organization (WHO) global air quality guidelines, the pollutants of greatest concern for public health include PM, CO, O_3_, NO_2_, and SO_2_ [20]. Short-term and long-term exposure to particulate matter has been associated with increased morbidity and mortality. This includes health effects on the cardiovascular [21, 22] and respiratory systems [21, 22], cancer [23, 24], diabetes [23, 24], and adverse pregnancy outcomes [23]. Short term exposure to CO can lead to tissue hypoxia and mortality [25], whereas chronic exposure has been linked to an elevated risk of myocardial infarction and hypoxic cardiac dysfunction, which can be fatal at high concentrations [26]. Exposure to elevated O_3_ levels can lead to respiratory issues, ultimately increasing overall mortality rates [27]. As regards SO_2_, major health problems are associated with the respiratory system, skin redness, ocular damage and worsening of pre-existing cardiovascular diseases [28]. Diesel exhaust particulate matter (DEP) —a major constituent of traffic-related air pollution (TRAP) and big contributor to urban atmospheric pollution— has been linked to several respiratory, cardiovascular and neurologic disorders [29–31].

Finally, it has been debated whether NO_2_ is a surrogate of air pollution in toxicological studies or it plays its own role in eliciting toxicological effects. The discussion was fuelled due to the difficulty to disentangle whether the effects observed after the exposure to NO_2_ were intrinsic or a consequence of synergistic effects from combined exposures, since NO_2_ is highly correlated with other environmental pollutants, such as PM [32] and noise [33–35]. However, studies like the EpiAir multicentric study found significant and likely independent short-term effects of nitrogen dioxide on natural, cardiac, and respiratory mortality [36]. In addition, a recent systematic review and meta-analysis identified that the certainty of the evidence linking NO_2_ exposure and COPD mortality was high [27].

At the molecular level, the assessment of biological outcomes such as cytotoxicity, cellular inflammation, oxidative stress and genotoxicity in cell cultures can be crucial to better understand the biological mechanisms underlying the impact of air pollution in human health [37].

While there is substantial literature investigating the range of mechanisms that are elicited upon exposure to individual pollutants using in vitro and in vivo models [38], relatively few studies have addressed the potential synergistic or additive effects of pollutant mixtures [39]. Therefore, accurate and reliable methods are required to assess the cellular changes and biological mechanisms associated with both gas and particulate air pollution, individually and in combination. This is not easily achievable with traditional in vitro pulmonary toxicity models, which typically involve cells suspended in a culture medium that exhibit low proliferation, significant senescence, and challenging differentiation [40–42]. These models are simpler from a methodological standpoint but physiologically unrealistic, since culture medium itself can confer a protective effect on cells that may mask the true effects of pollutants [43]. Such limitations could be solved by using novel air–liquid interface (ALI) systems, which represent one of the most promising approaches in respiratory toxicology as an alternative to animal testing [44–47]. These systems closely mimic the characteristics of the pulmonary epithelial cells, including tight junction formation and cellular differentiation, while maintaining an extracellular environment highly similar to in vivo models [44].

Lakhdar et al. (2022) [48] have reviewed the available literature and offered a valuable overview on the biological changes elicited upon exposures to air pollutants based on toxicological studies using ALI systems. Their review pays particular emphasis on cell culture and exposure systems, as well as on cellular processes such as senescence, mitochondrial damage, and autophagy as emerging markers of pollutant-induced toxicity. On the other hand, oxidative stress and inflammation response as a biological mechanism underlying the health effects are less considered in Lakhdar´s review [48]. The review by Cho et al*.* [49], which included also some studies conducted using ALI systems, identified oxidative stress, inflammation and genotoxicity as key mechanisms involved in PM_2.5_. However, they did not review the evidence as regards gaseous pollutants, other size fractions beyond PM_2.5_, or a mixture of gases and aerosols. New evidence has also been generated since Cho et al*.* [49] conducted their review.

The main objective of this systematic review is to comprehensively evaluate the existing scientific evidence regarding the impact of air pollution on viability, cytotoxicity, inflammation, oxidative stress and genetic damage in human airway cellular models. We aim to achieve this by summarizing findings from studies using innovative air–liquid interface (ALI) systems. By integrating evidence from ALI systems, we seek to provide a better understanding of the biological mechanisms underpinning the health effects of air pollution and evaluate the advantages of ALI systems in respiratory toxicity assessment.

## Methods

A systematic review was conducted to study the toxicological effects produced on airway cell models upon exposure to several airborne pollutants using *Air–Liquid Interface* (ALI) systems. The PRISMA standards were followed as reporting guidance for this review (PRISMA checklist in Supplementary Information).

### Information Sources and Search Strategy

A literature search was conducted in PubMed, Web of Science and Scopus databases for all studies published up to December 2022. The following keywords associated with ALI exposure systems and air pollutants of bigger concern (based on the WHO Air Quality Guidelines) were selected: “air–liquid cellular model”, “air–liquid interface culture”, “human respiratory tract”, “human respiratory cellular model”, “particulate matter”, “PM”, “PM_2.5_”, “PM_10_”, “PM_coarse_”, “nitrogen dioxide”, “carbon monoxide”, “sulfur dioxide”, “sulphur dioxide”, “ozone”, “aerosol”, “aerosols”, “air pollution”, “air pollutants”, “inflammation”, “oxidative stress”, “cytotoxicity”, “gene expression”, “proliferation”.

An update on March 11, 2024 identified 30 additional studies through database searching, with one study meeting the inclusion criteria.

### Eligibility Criteria

Studies were included in the review according to the following criteria:

1. Experimental studies in which the use of a specific in vitro cell culture design based on an ALI system was reported.
2. Exposure agents in the study are one of the key outdoor pollutants established by the WHO, such as PM_2.5_, PM_10_, O_3_, NO_2_, SO_2_ or CO.
3. Exposure agents in the study include diesel or gasoline exhaust.
4. Studies focus on the following biological outcomes: cytotoxicity, cell viability, cellular inflammation, oxidative stress, or genotoxicity.
5. Values of the outcomes produced upon exposure of the target air pollutant agents are clearly reported.

Some articles were excluded because the outcomes of interest were not measured, or the results of the outcomes were not reported. More specifically, articles were excluded according to any of the following exclusion criteria:

1. Studies related with tobacco smoke, e-cigarettes or water pipe smoke.
2. In vivo studies or used animal cellular models.
3. Studies based exclusively on traditional in vitro cellular models under submerged conditions.
4. Studies related with wood smoke, biomass smoke or cookstove emissions.
5. Studies related with engineering nanomaterials.
6. Studies not written in English.
7. Review articles.

Additional articles cited in the studies reviewed were also included whenever compliant with the eligibility criteria.

### Selection Process

**Fig. 2:**
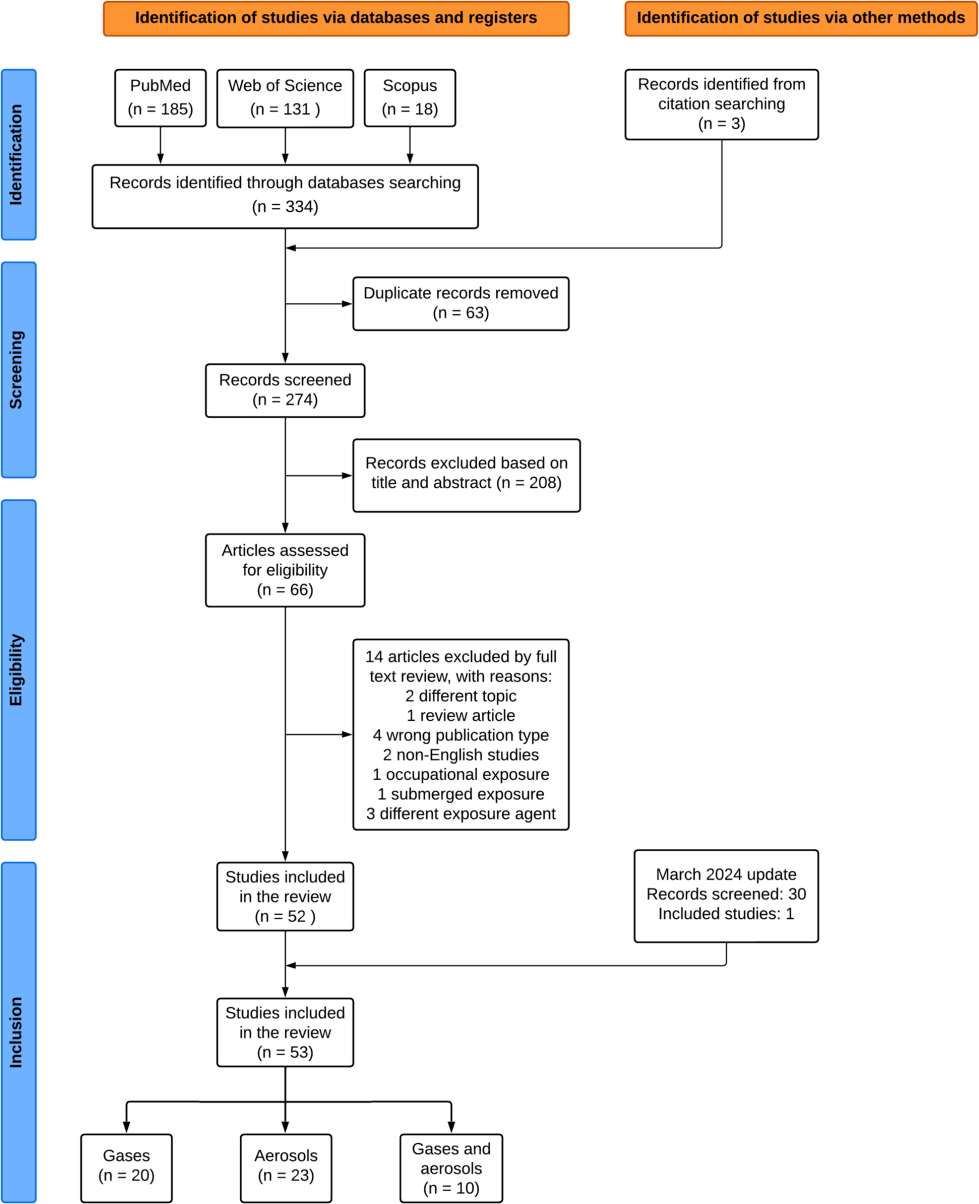
Flow diagram of study selection based on the PRISMA statement

All the articles found in the literature search were imported into Rayyan, a tool for systematic reviews. First, duplicates were resolved for the 337 articles identified through database searching. Then, the title and abstract screening was performed by two independent reviewers (ONA and JMDS), and conflicts were solved by a third reviewer (MMS). In the second phase, the full text of articles selected for inclusion was reviewed by ONA and JMDS, and controversies in this stage were resolved by MMS. Selected articles were classified in three groups depending on the exposure agent, which were 1) exposure to gases, 2) exposure to aerosols or PM and 3) combined exposure to gases and aerosols. The whole selection process is depicted in Fig. 2.

### Data Collection Process and Items

Data was collected by one reviewer (ONA) and supervised by the other two reviewers (JMDS and MMS). An excel datasheet (Supplementary Information) was created for each group of articles to extract all the relevant information methodically, including the following fields: Author & publication year, location, ALI culture type, cell type, culture conditions, biological and technical replicates, exposure agent, exposure system, exposure duration, exposure concentration, outcomes of interest, outcome measurement, results, statistical analysis and controls.

### Study of Risk of Bias Assessment

The risk of bias (RoB) assessment was performed using the NTP Handbook for Conducting a Literature-based Health Assessment Using OHAT Approach for Systematic Review and Evidence Integration [50]. Studies were rated and classified by one reviewer (ONA) and revised by the other two reviewers (JMDS and MMS). All questions and criteria selected for the RoB assessment of the in vitro epidemiology studies included in this review are presented in the Supplementary Information (Tables S1-S3). Three key criteria were highlighted (exposure characterization, experimental conditions, and outcome assessment), and four additional quality criteria were selected (statistical approach, selective reporting bias, adequate randomization, and temporality).

In addition to the quality criteria mentioned above, a tiering approach according to the OHAT Handbook was applied. This method supports the inclusion of most studies in the body of evidence, except those with a high risk of bias across multiple key quality domains. Following the guidelines of the OHAT Handbook, results from articles rated as Tier 3 were not considered in the integration of evidence due to their high risk of bias.

## Results

A total of 53 articles were included in the review. Twenty studies reported the effect of exposure to gases (Table 1), 23 studies focused on aerosols or particulate matter exposures (

**Table 1:** Summary of characteristics and outcome assessment of studies reporting the effects of exposure to gaseous pollutants included in the literature review

Reference	Location	ALI culture	Cell type(s)	Exposure agent(s)	Exposure system	Exposure dose	Exposure duration	Outcome assessment			
								**Cell viability/Cytotoxicity**	**Cellular inflammation**	**Genotoxicity**	**Oxidative stress**
[51]	Sydney, Australia	DMEM/Ham's F12 mixture, snapwell inserts	A549, skin fibroblasts	NO_2_	NaviCyte horizontal diffusion chamber	5000–20000 µg/m^3^ NO_2_	0.5–2 h	✓			
[52]	Würzburg, Germany	BEGM, collagen-coated inserts	Human nasal epithelial cells (HNE)	NO_2_	Vitrocell® system	20–20000 µg/m^3^ NO_2_	0.5 h	✓		✓	
[53]	Würzburg, Germany	BEGM, collagen-coated inserts	HNE	NO_2_	Vitrocell® system	200 µg/m^3^ NO_2_	0.5–3 h	✓		✓	
[54]	Würzburg, Germany	BEGM, collagen-coated inserts	HNE	NO_2_	Vitrocell® system	20 µg/m^3^ NO_2_	0.5–3 h	✓		✓	
[55]	Würzburg, Germany	BEGM, collagen-coated inserts	HNE	NO_2_ + Der p1	Vitrocell® system	200–20000 µg/m^3^ NO_2_	1 h		✓		
[56]	Mainz, Germany and Louisiana, USA	DMEM/Ham's F12 mixture, 3 × 10^5^ cells/mL, collagen-coated inserts	HNE	O_3_	Collagen-coated polycarbonate-membrane	100, 500 and 1000 µg/m^3^ O_3_	4 weeks **(long-term)**	✓	✓		
[57]	Ottawa, Canada	DMEM, collagen-coated PTFE inserts	A549	O_3_	Exposure chamber based on CelTox Sampler	400 µg/m^3^ O_3_	2 h	✓			
[58]	North Carolina, USA	BEGM and KBM, transwell inserts	HBEpC, BEAS-2B	O_3_	US EPA exposure chambers with standard conditions	1000 µg/m^3^ O_3_	2 h		✓		✓
[59]	North Carolina, USA	BEGM/DMEM-H mixture, transwell inserts	HBEpC	O_3_	US EPA exposure chambers with standard conditions	1000 µg/m^3^ O_3_	2 h		✓		✓
[60]	North Carolina, USA	F-12-K complete medium, collagen-coated Snapwell inserts	A549, Epiairway™ 3D	O_3_	Gas In Vitro Exposure System (GIVES) with standard conditions	800 µg/m^3^ O_3_	4 h	✓	✓		
[61]	North Carolina, USA	BEGM, transwell inserts	HBEpC	O_3_, NO_2_	Exposure chambers with standard conditions	2000–10000 µg/m^3^ NO_2_	2 h		✓	✓	✓
[62]	Hannover, Germany	RPMI 1640, 3.5 × 10^4^ Lk004 and 2.0 × 10^4^ HFBE-21 cells/cm^2^	Lk004, HFBE-21	O_3_, NO_2_	Cultex®-based system	400 and 1000 µg/m^3^ O_3_, 150–2400 µg/m^3^ NO_2_	1–2 h	✓			✓
[63]	Paris, France	Ham’s F12, collagen-coated inserts	A549	HCHO	Vitrocell® system	25–75 µg/m^3^ FA	0.5 and 1 h	✓			
[64]	Paris, France	Ham’s F12, collagen-coated inserts	A549, BEAS-2B	HCHO	Vitrocell® system	50 µg/m^3^ FA	0.5 h	✓	✓		
[65]	Innsbruck, Austria	DMEM/Ham's F12 mixture, polyester membrane inserts	A549	HCHO	Polypropylene exposure chamber with standard conditions	120 and 610 µg/m^3^ FA	72 h	✓		✓	
[66]	Strasbourg, France	DMEM/Ham's F12 mixture, PET inserts	Calu-3	HCHO, NO_2_	Polystyrene exposure chamber with standard conditions	50–200 µg/m^3^ HCHO, 200–800 µg/m^3^ NO_2_	0.5, 1 or 2 h	✓	✓		
[67]	Queensland, Australia	DMEM, hanging drop culture and Alvetex® scaffold inserts	A549	Benzene, toluene and xylenes	Hanging drop Medium diffusion Periodic exposure Apical surface exposure	5 and 10 µL benzene	1-h and 24-h acute exposures 1–20 days chronic exposure **(long-term)**	✓	✓	✓	
[43]	Birmingham, UK	RPMI 1640, collagen-coated inserts	A549	Benzene	Cultex®-based system	96–960 µg/m^3^ benzene	2 and 4 h			✓	✓
[68]	Mol, Belgium	MEM, ThenCert™ polystyrene membrane inserts	A549	Ethylbenzene, NO_2_	Vitrocell® system	3 × 10^7^—5 × 10^7^ µg/m^3^ EB, 40,000 µg/m^3^ NO_2_	4 h	✓	✓		✓
[69]	Beirut, Lebanon	DMEM, transwell inserts	A549	Gasoline VOCs	Static exposure system	3–735 ppm	1 h	✓	✓	✓	

Table 2) and 10 studies assessed the combined effect of gases and aerosols (Table 3). The included literature originates mainly from Europe (34), followed by North America (15) and Asia (3). Additionally, two studies reported data from Australia.

**Table 2:** Summary of characteristics and outcome assessment of studies reporting the effects of exposure to aerosols and particulate matter included in the literature review

Reference	Location	ALI culture	Cell type(s)	Exposure agent(s)	Exposure system	Exposure dose	Exposure duration	Outcome assessment			
								**Cell viability/Cytotoxicity**	**Cellular inflammation**	**Genotoxicity**	**Oxidative stress**
[70]	North Carolina, USA	MEM and DMEM/Ham’s F12 mixture, transwell-clear inserts	Calu-3, A549	DEP	Direct aerosol exposure and liquid exposure, 24 h incubation	10–100 µg/cm^2^	n.d	✓	✓		✓
[71]	Cluj-Napoca, Romania	75% DMEM, 15% RPMI 1640 and 10% IMDM, BD Falcon inserts	A549, Ea.hy 926, THP-1, HMC-1 (tetraculture)	DEP	Vitrocell® system;	1.75–5 µg/cm^2^	2.5–20 min	✓	✓	✓	
[72]	California, USA	MEM, collagen-coated inserts	16HBE14o	DEP	ALI exposure at an environmental chamber and particle suspension exposure	1 × 10^–4^ µg/cm^2^ **(ALI)** 0.13–12.5 µg/cm^2^ **(suspension)**	6 h	✓	✓		
[73]	Sweden	PneumaCult™-Ex medium, transwell inserts	PBEC, THP-1	DEP	XposeALI system	12.7 µg/cm^2^	3 min	✓	✓		✓
[74]	Esch-sur-Alzette, Luxembourg	DMEM. RPMI 1640, IMDM, BD falcon inserts	A549, Ea.hy 926, THP-1, HMC-1 (tetraculture)	DEP	Vitrocell® system	0.04–0.24 µg/cm^2^	1–7 min	✓	✓		✓
[75]	Fribourg, Switzerland	RPMI 1640, PET membrane inserts	16HBE14o, MDMs, MDDCs	DEP	Exposure chambers with standard conditions	1.7 × 10^7^ DEP/cm^2^ 7.4 × 10^7^ DEP/cm^2^	2 h 6 h	✓	✓		✓
[76]	Paris, France	DMEM/F12: BEGM mixture, collagen-coated inserts	HNE	DEP, PM_2.5_	Particle suspension exposure	10–80 µg/cm^2^	24 h	✓	✓		✓
[77]	Bern. Switzerland	DMEM, collagen-coated inserts	NHBE, distressed HBE, CF HBE, BEAS-2B	GEP	Exposure chamber with standard conditions	20–1000 µg/m^3^	2 h	✓	✓		
[78]	Fribourg, Switzerland	RPMI 1640, BD falcon inserts	A549, MDMs, MDDCs	Brake wear PM	Environmental chamber simulating urban driving; exposure,	12–48 µg/cm^2^ non-airborne samples, 3.7 µg/cm^2^ brake wear particles	24 h	✓	✓		✓
[79]	Bern, Switzerland	RPMI 1640, BD falcon inserts	A549	Brake wear PM	Exposure box with standard conditions; direct exposure to PM with different braking behaviours and 24-h incubation	1760–4730 µg/m^3^	8–16 min	✓	✓		✓
[80]	North Carolina, USA	BEGM and KBM, collagen-coated inserts	NHBE, BEAS-2B	UPM	Particle suspension exposure	2–10 µg/µL UPM	4 or 24 h	✓	✓		✓
[81]	Californa, USA	BEGM, transwell inserts with polyester membranes	AECs	UPM	Particle suspension exposure	1 mg/mL UPM	4 days		✓	✓	
[82]	Singapore	S-ALI™/PneumaCult™-ALI differentiation media	SAECs	Urban PM_2.5_ surrogates	Particle suspension exposure	7–30 µg/m^3^	24 h	✓	✓	✓	
[83]	Lille, France	BEGM, polyester transwell inserts	NHBE, COPD-DHBE	PM_4_	Particle suspension exposure	1–20 µg/cm^2^ PM_4_	4 h	✓	✓	✓	✓
[84]	Bern, Switzerland	n.d	HBE, CF HBE	PM_2.5_, PM_10_	Exposure to water-soluble filter extracts	0.9–2.5 μg/cm^2^ **(low dose)** 8.8–25.4 μg/cm^2^ **(high dose)**	4 h	✓	✓	✓	
[85]	Colorado, USA	BEGM, collagen-coated inserts	NHBE	PMcoarse	Direct-air exposure Indirect liquid exposure	2 µg/cm^2^ 7–65 µg/cm^2^	3 h	✓	✓		
[86]	Paris, France	MucilAir nutrient medium, ALI culture on inserts	Human bronchial epithelial cells and human airway fibroblasts	PM_2.5–0.3_	Exposure to 10 µL of PM twice a week	45 or 90 µg/cm^2^	3 weeks **(long-term)**			✓	
[87]	Lille, France	BEGM, collagen-coated transwell inserts with polyester membranes	NHBE, asthma and COPD-diseased HBE	FP, UFP	Acute and repeated exposures to particle suspension	5 µg/cm^2^	6 h or 3 × 6 h exposure with 18-h intervals	✓	✓	✓	
[88]	Netherlands	MEM, transwell inserts with polyester membrane	Calu-3	UFP from aviation and road traffic	Vitrocell® system	0.09—2.07 µg/cm^2^		✓	✓		
[89]	Utah, USA	DMEM, collagen-coated inserts	A549, THP-1	Jet-fuel surrogate particles	CelTox sampler system, ALI, pseudo-ALI and submerged exposures	2–6 µg/cm^2^	0.5 or 1 h	✓	✓		✓
[90]	Munich, Germany	DMEM/F-12, Anodisc filter membranes (47 mm, 0.2 µm pore size)	A549	Aerosol generated from ultrafine carbonaceous particles (C-ufp)	Minucell system	44–230 ng/cm^2^	6 h	✓	✓		✓
[91]	Bern, Switzerland	n.d	NHBE	Aerosol from soot particles and SP coated with SOM (α-pinene and mesitylene)	Exposure to atmospherically aged SP aerosol	9–279 μg/m^3^ SP per week	1 h	✓	✓		
[92]	Germany	DMEM/F-12, transwell inserts	A549, Ea.hy926	Soot particles (SP), secondary organic aerosol (SOA)	Vitrocell®-based custom-made system	1000 μg/m^3^ SP, 4000 μg/m^3^ naphtalene or β-pinene	4 h	✓	✓	✓	✓

**Table 3:** Summary of characteristics and outcome assessment of studies reporting the effects of exposure to particles and gases included in the literature review

Reference	Location	ALI culture	Cell type(s)	Exposure agent(s)	Exposure conditions	Exposure dose	Exposure duration	Outcome assessment			
								**Cell viability/Cytotoxicity**	**Cellular inflammation**	**Genotoxicity**	**Oxidative stress**
[93]	California, USA	MEM, collagen-coated inserts	16HBE14o	Diesel exhaust (DE)	Environmental chamber exposure	5–7.2 ppm NOx	4–6 h	✓	✓		
[94]	Netherlands	DMEM:BEGM mixture, transwell inserts (40,000 cells/cm^2^)	PBECs	Diesel exhaust (DE)	Vitrocell® exposure system	0.04–1.87 µg/cm^2^	60–375 min	✓	✓	✓	
[95]	Brisbane, Australia	B-ALI medium, collagen-coated inserts	HBECs	Diesel and biodiesel emissions	Cultex® radial flow system	0.74–1.30% CO_2_, 29.36–65.43 ppm NOx, 60–790 µg/m^3^ PM	30 min	✓	✓	✓	✓
[96]	Sweden	PneumaCult™ medium, pre-coated transwell inserts (10^5^ cells/insert)	PBECs	Diesel exhaust particles (DEP), NO_2_, SO_2_	Humidified exposure chamber	12.5 μg/cm^2^ DEP, 190 or 380 μg/m^3^ NO_2_, 520 or 1040 μg/m^3^ SO_2_	0.5 h acute exposure or 0.5 h/day (3 days)	✓	✓	✓	
[97]	Hannover, Germany	RPMI 1640, PET track-etched membranes	HFBE-21	Motor exhaust (ME)	Cultex® exposure chamber	35 ppm HC, 1–50 ppm CO, 1–30 ppm NOx, 1.5–2 ppm CH_4_ and 1 × 10^3^–1 × 10^9^ particles/cm^3^	1 h	✓			
[98]	Beijing, China	DMEM, transwell inserts	A549, BEAS-2B	Motorcycle Exhaust (ME)	Direct aerosol exposure	227.5 μg/m^3^ PM_10_, 289.4 μg/m^3^ PM_2.5_ and 14.6 μg/m^3^ PM_0.1_ for non-filtered ME; 5.1 μg/m^3^ PM_2.5_ and 0.8 μg/m^3^ PM_0.1_ for filtered ME	1 h for A549 cells 0.5–1.5 h for BEAS-2B cells	✓			✓
[99]	Czech Republic	BEGM and MucilAir™ medium, transwell inserts (10^5^ cells/insert)	BEAS-2B, MucilAir™	Complete gasoline exhaust	World Harmonized Light Vehicle Test Cycles (WLTC) as exposure unit	175 µg/m^3^ PM	30-min exposures up to 5 days	✓		✓	
[100]	Czech Republic	BEGM and MucilAir™ medium (10^5^ BEAS-2B cells/insert)	BEAS-2B, MucilAir™	Complete gasoline-ethanol blend exhaust (E20)	Toxicological incubator with an exposure chamber	0.05 mg/m^3^ PM, 0.02 mg/m^3^ black soot	1 or 5 days	✓		✓	
[101]	Czech Republic	BEGM and MucilAir™ medium, transwell inserts	BEAS-2B, MucilAir™	Complete gasoline exhaust, gasoline- ethanol blend exhaust and EOM	Toxicological incubator with an exposure chamber	65–330 pg PAHs/insert	1 or 5 days		✓		
[102]	Fribourg, Switzerland	RPMI, collagen-coated inserts	16HBE14o, MDM, MDDC	Ambient air from Fribourg (PM_10_, O_3_, NO_2_, NO and NOx 30-min averages)	Mobile cell exposure system	30 μg/cm^3^ PM_10_, 28 μg/cm^3^ O_3_ and 34 μg/cm^3^ NO_2_ (winter) and 17 μg/cm^3^ PM_10_, 87 μg/cm^3^ O_3_ and 8 μg/cm^3^ NO_2_ (summer)	12 h	✓	✓		✓

In these studies, a variety of healthy and diseased respiratory cell lines were used to develop different air–liquid interface (ALI) cellular models that closely represented the human pulmonary system. These cell lines included human alveolar adenocarcinoma cells (A549) in 19 studies, human nasal epithelial cells (HNE) in 6 studies. Human bronchial epithelial cells were used in several studies, including HBEpC in 7 studies, BEAS-2B in 7 studies, 16HBE14o- in 4 studies, HFBE-21 in 2 studies, HBE in 2 studies and NHBE in 5 studies. Human lung fibroblasts (Lk004) were used in one study, human epithelial lung adenocarcinoma cells (Calu-3) in 3 studies, small airway epithelial cells (SAECs) in one study and primary airway epithelial cells (AECs) in one study. However, in certain studies cells were obtained directly from patients undergoing sinus surgery [52–55], or through enzymatic digestion of bronchial tissue [94]. These approaches required additional procedures to isolate the specific cell types of interest.

**Fig. 3:**
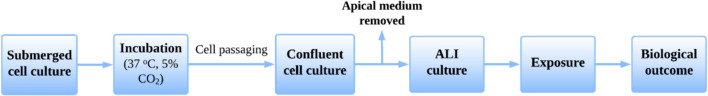
Schematic representation of the general experimental timeline for in vitro toxicology studies using ALI systems

As a standard protocol, cells were cultured in flasks using the most suitable culture medium for each specific cell type. Commonly used culture media included Airway Epithelial Cell Growth Medium (BEGM) in 13 studies, Minimum Essential Medium (MEM) in 5 studies, Ham’s F12 in 9 studies, RPMI 1640 in 5 studies and Dulbecco's Modified Eagle Medium (DMEM) in 13 studies. Following culture, cells were transferred to inserts with porous membranes, typically made of PET, and sometimes coated with collagen. These cells were then incubated under standard conditions, including a temperature of 37 ºC and an atmosphere with 5% CO_2_, to mimic physiological conditions and support cell viability. During this incubation period, routine cell culture procedures such as passaging, culture media replacement and cell counting were implemented to maintain optimal cell density and ensure consistent growth. Once the cells reached confluence, typically within one week, the apical medium was aspirated to initiate the establishment of the ALI. This involved maintaining the cell monolayer with medium supplied only to the basal compartment. The ALI culture was sustained for at least one additional week, during which the apical surface was periodically rinsed with phosphate-buffered saline (PBS) to remove accumulated mucus and cellular debris, helping to preserve epithelial integrity and function. The basal medium was refreshed every other day to provide nutrients to the cells and remove waste products. This whole process is depicted in Fig. 3.

In some cases, cell lines were co-cultured with macrophages and dendritic cells to create more physiologically relevant ALI systems. Additionally, complex three-dimensional ready-to-use models were used to better mimic in vivo conditions. Examples include the EpiAirway™ 3-D model derived from human tracheal/bronchial epithelial cells [60], the MucilAir™ 3-D model representing nasal epithelium [99, 101], and the MucilAir-HF™ [86]. As described in a previous review by Lakhdar et al*.* [48], several studies proposed alternative in vitro systems known as organs-on-chip, which emulate the complex features of the respiratory epithelium, such as tight junction formation or mucin secretion.

**Fig. 4:**
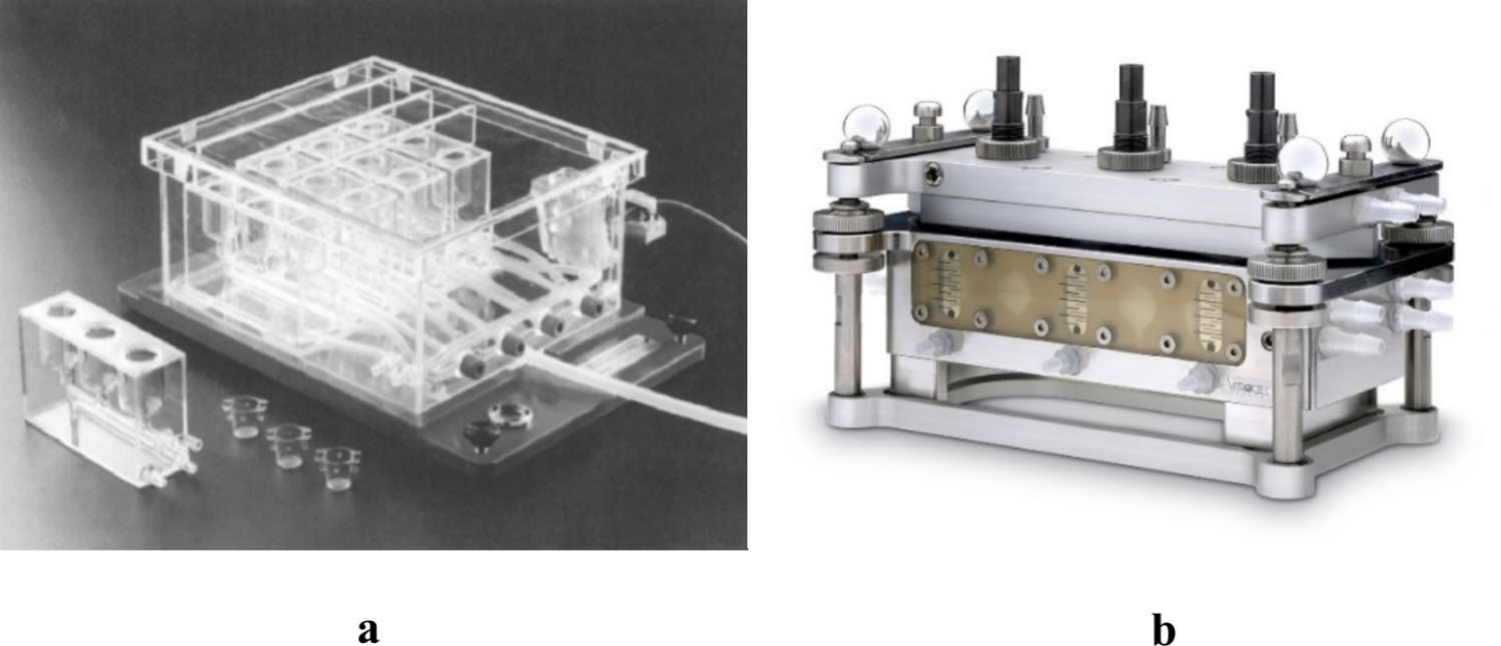
Examples of commercial ALI Systems: a) CULTEX ® exposure chamber [103] and b) Vitrocell ® exposure chamber. Extracted from https://www.vitrocell.com/inhalation-toxicology/exposure-systems/for-6-well-inserts/6-3-cf-stainless-steel/. Others (not pictured) include NaviCyte horizontal diffusion chamber [51], MINUCELL perfusion unit [90], XposeALI system [73], and the ExpoCube device [48]

The ALI systems mentioned above were exposed to various airborne contaminants using diverse exposure setups. Two particularly significant ones were the CULTEX® and Vitrocell® exposure chambers. The CULTEX® exposure chamber (Fig. 4) was used in two studies [95, 97], and user made adaptations of it were used in one study [43]. The Vitrocell® system (Fig. 4) was used in 11 studies [52–55, 63, 64, 68, 71, 74, 88, 94], and user made adaptations of it were used in two studies [62, 92]. Some other examples of exposure devices are the NaviCyte horizontal diffusion chamber [51], the MINUCELL perfusion unit [90], the XposeALI system [73], and the ExpoCube device [48].

Regarding the exposure agents of interest, the impact of the main urban pollutants defined by the WHO [104] on human health was assessed in most studies. In the case of gaseous pollutants, 9 studies evaluated the effects of exposure to nitrogen dioxide (NO_2_) and 7 studies reported ozone (O_3_) exposure. Three studies focused on benzene and benzene derivatives such as ethylbenzene, toluene and xylenes, while volatile organic compounds (VOCs) from gasoline were tested in one study [69]. Additionally, the effects of formaldehyde, a common indoor air pollutant, were evaluated in 4 studies.

When considering aerosols and particulate matter, exposure to urban particulate matter (UPM) was reported in two studies, and DEP were studied in 8 articles as one of the main traffic-related sources of pollution in urban environments. However, cell cultures were also exposed to more specific types of particulate matter, such as PM_2.5_ in 3 studies, PM_10_ in one study, PM_4_ in one study, and ultrafine particles (UFP) in 3 studies. In studies that simultaneously assessed gas and aerosol exposures, mixtures of the pollutants mentioned before were employed, as well as complete motor exhaust or ambient air.

Most studies included in this review involved exposure durations of up to 72 h, aligning with the definition of *short-term exposure* established by Pfaller et al*.* [105]. In contrast, only three studies [56, 67, 86] evaluated *long-term exposure* characterized by durations exceeding 5 days.

As for the biological outcomes, cytotoxicity was assessed in 45 studies and cellular inflammation in 40 studies. Induction of oxidative stress was reported in 26 studies, especially in the case of aerosol pollutants, and genotoxic effects of the exposure agents were evaluated in 16 studies. Additionally, the trans-epithelial electrical resistance (TEER) was measured in 6 studies as an indicator of cell monolayer membrane integrity and strength of tight junctions formed between cells. The oxidative potential of particulate matter was measured in one study after the extraction of urban PM_10_ and PM_2.5_ samples in simulated lung fluid [84]. Depletion of various antioxidants was quantified using spectrophotometry through dithioerythritol (DTT), dichlorofluorescein (DCF), and ascorbic acid (AA) assays. Description of the main findings by biological outcome are presented below. Detailed description of individual study main findings by biological outcome can be found in the Supplementary Information.

### Cell Viability and Cytotoxicity

Cytotoxicity refers to the ability of an agent to cause damage or death to living cells, and its quantification allows for the assessment of how different compounds ultimately affect cell physiology or viability [106, 107]. The concept of cytotoxicity does not necessarily imply immediate cell death, but rather damage to the cell, which often leads to death. However, commonly used tools to evaluate the degree of cytotoxicity include in vitro assays that either measure cell viability, such as the MTT assay [108], or assess the extent of cell damage or death, such as the LDH assay [109], which detect membrane integrity loss by quantifying the release of lactate dehydrogenase into the culture medium.

In the studies reviewed, a variety of cell viability and cytotoxicity assays were employed. The trypan blue assay was used in 5 studies [52, 53, 73, 82, 110], the MTT assay in 5 studies [66, 68, 70, 72, 93], the WST-1 assay in 4 studies [62, 90, 95, 97], and the resazurin assay in 4 studies [57, 65, 74, 92]. The MTS assay was employed in 4 studies [51, 67, 69, 88], the CellTiterGlo® assay in 3 studies [51, 83, 87] and the CCK-8 assay in 2 studies [89, 98]. Additionally, the XTT assay was used in one study [63] and another study utilized the NRU assay [51].

The lactate dehydrogenase (LDH) assay was used in 24 studies [56, 60, 64, 66, 68–71, 75–77, 79, 80, 84, 85, 88, 91, 92, 94–96, 99, 100, 102], whereas the Annexin V assay was only used in 4 studies [69, 73, 78, 110]. Additionally, G6PD [83, 87] and Caspase-3 (CASP3) [54, 82] assays were used respectively in two studies. Changes in the expression of some genes involved in apoptosis such as B-cell leukemia/lymphoma 2 protein (BCL2) and CASP3 were assessed by Reverse transcription polymerase chain reaction (RT-PCR) in two studies [75, 95].

#### Gaseous Pollutants

A summary of the studies evaluating the cytotoxic effects of gaseous pollutants on airway epithelial cells is provided in Table 4.

**Table 4:**
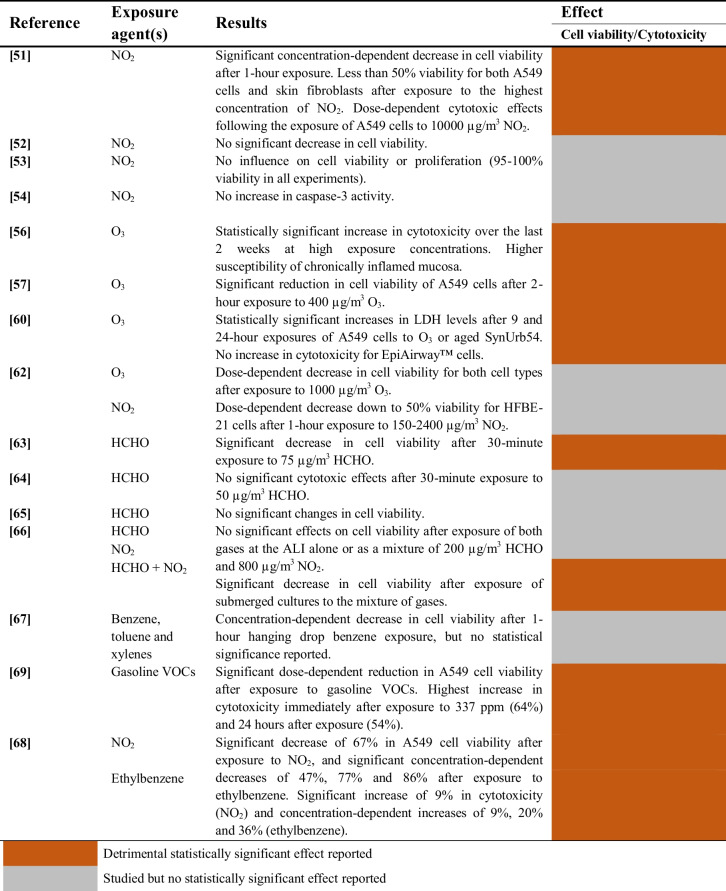
Cytotoxic effects of gaseous pollutants on airway epithelial cells

##### Nitrogen dioxide (NO2)

The cytotoxicity of NO_2_ was examined in 10 studies. Two studies reported that NO_2_ elicits a dose-dependent cytotoxic effect on airway epithelial cells with the corresponding decrease in cell viability [51, 68]. However, such effect might not reach statistical significance after short exposure durations and/or at low exposure concentrations [51]. On the other hand, three studies did not report any effect on cell viability or proliferation [52–54], whereas one study did not include any statistical analyses [62]. Similarly, two studies did not report any cytotoxic effects [54, 66].

##### Ozone (O3)

Ozone exposure consistently showed cytotoxic effects on airway epithelial tissues across 4 studies. Two of these studies report statistically significant and dose-dependent cytotoxicity [56, 60], except for EpiAirway™ cells in one study [60]. One study shows significantly reduced cell viability of A549 cells [57]. Similarly, one study observed a substantial decrease in cell viability for two different cell types, but the authors did not report any statistical analyses [62].

##### Volatile Organic Compounds (VOCs)

The cytotoxic effects of different volatile organic compounds (VOCs) were tested in 7 studies, including 4 studies focused on formaldehyde [63–66], one study on benzene, toluene and xylenes [67], one study on gasoline VOCs [69], and another study on ethylbenzene [68]. Results suggest that there is a detrimental effect of VOCs on A549 cells, since significant cytotoxic effects were reported after exposure to gasoline VOCs [69] or ethylbenzene [68]. However, no statistical significance was reported for benzene, toluene and xylenes using a hanging drop exposure method [67]. Finally, only one study was able to report statistically significant cytotoxicity following formaldehyde exposure [63].

#### Aerosols and Particulate Matter (PM)

A summary of the studies evaluating the cytotoxicity of aerosols and particulate matter on airway epithelial cells is provided in Table 5.

**Table 5:**
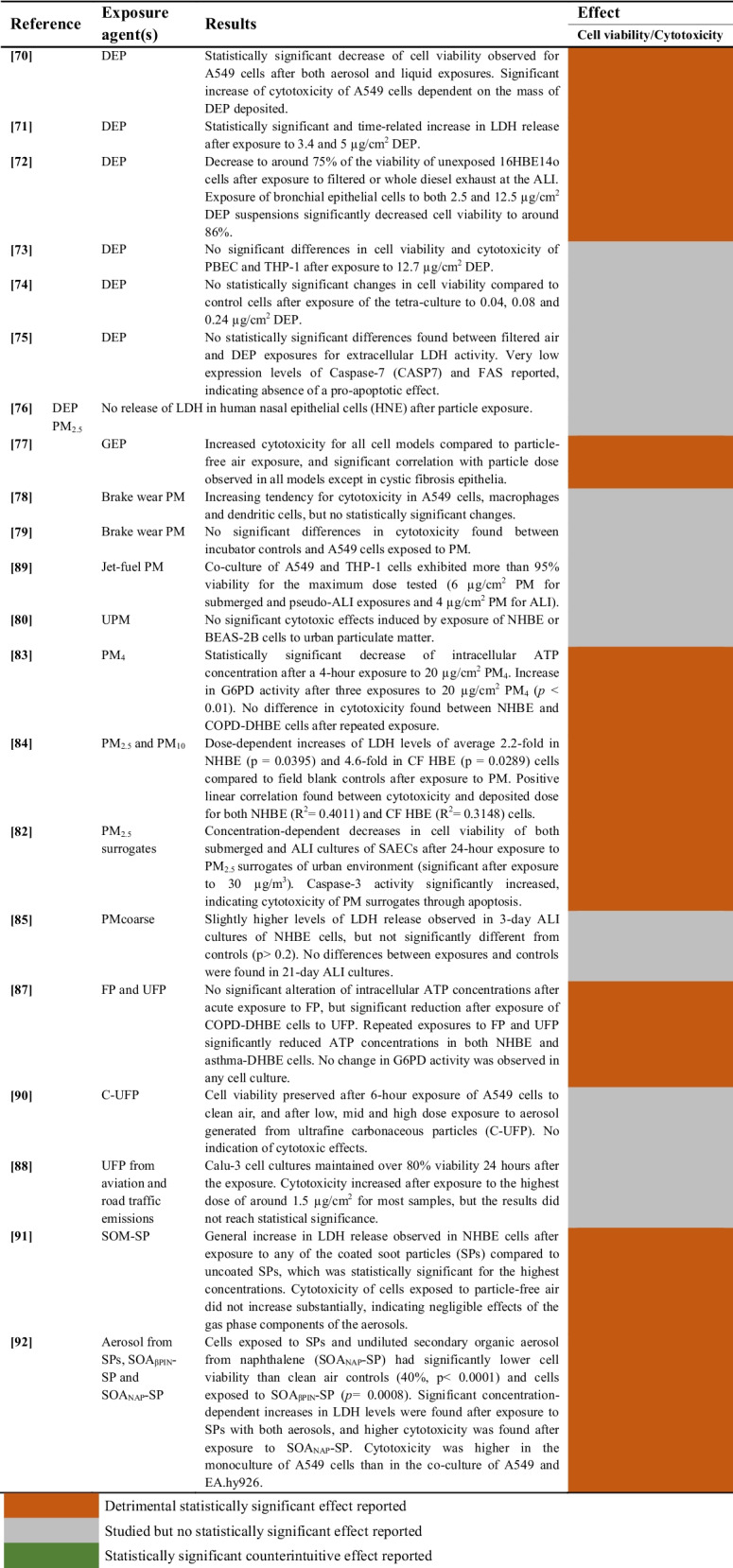
Cytotoxic effects of aerosols and PM on airway epithelial cells

##### Diesel Exhaust Particles (DEP)

The cytotoxicity of diesel exhaust particles was evaluated in 7 studies. Three studies reported statistically significant cytotoxic effects of DEP [70–72], while 4 studies did not find significant variations following DEP exposure [73–76]. Moreover, no significant differences in cytotoxicity were observed following DEP filtration [75].

##### Particulate Matter (PM) and Secondary Aerosol from Soot Particles (SP)

The cytotoxicity of particulate matter (PM) from brake wear debris was evaluated in two studies [78, 79]. No significant differences between the particle-exposed group and incubator controls were reported. Similarly, no decrease in cell viability was observed in A549 cells and THP-1 macrophages exposed to jet-fuel particulate matter [89].

On the other hand, the impact of fine and coarse particulate matter was tested in 4 studies [82–85]. Cytotoxicity increased significantly after exposure to PM_4_ [83] and both PM_2.5_ and PM_10_ [84] in healthy and diseased human bronchial epithelial cell cultures. Additionally, cell viability of Small Airway Epithelial Cells (SAECs) decreased significantly after exposure to PM_2.5_ surrogates of urban environment [82]. A slight increase of cytotoxicity of ALI cultures was reported following exposure to PM_coarse_, although this effect did not reach statistical significance [85].

Cell cultures exposed repeatedly to fine particles (FP) and ultra-fine (UFP) particles experienced a significant reduction of cell viability in one study [87]. Interestingly, the cytotoxic response was exacerbated by exposure to UFP in the case of diseased human bronchial epithelial cells (COPD-DHBE). However, aerosol generated from carbonaceous ultrafine particles (C-UFP) did not induce cytotoxic effects on A549 cells [90], and the effects of UFP from aviation and road traffic emissions were not statistically significant [88].

Finally, the impact of exposure to secondary organic matter and aerosols derived from soot particles (SPs) was assessed in two studies [91, 92]. The cytotoxicity increased significantly for NHBE cells after exposure to high concentrations of coated SPs, whereas cells exposed to particle-free air did not experience substantial increases [91]. Moreover, exposure to SPs with aerosols from naphthalene and β-pinene had significant concentration-dependent cytotoxic effects on A549 cells [92].

#### Combined Aerosols and Gaseous Pollutants

A summary of the studies evaluating the combined cytotoxic effects of aerosols and gaseous pollutants on airway epithelial cells is provided in Table 6.

**Table 6:**
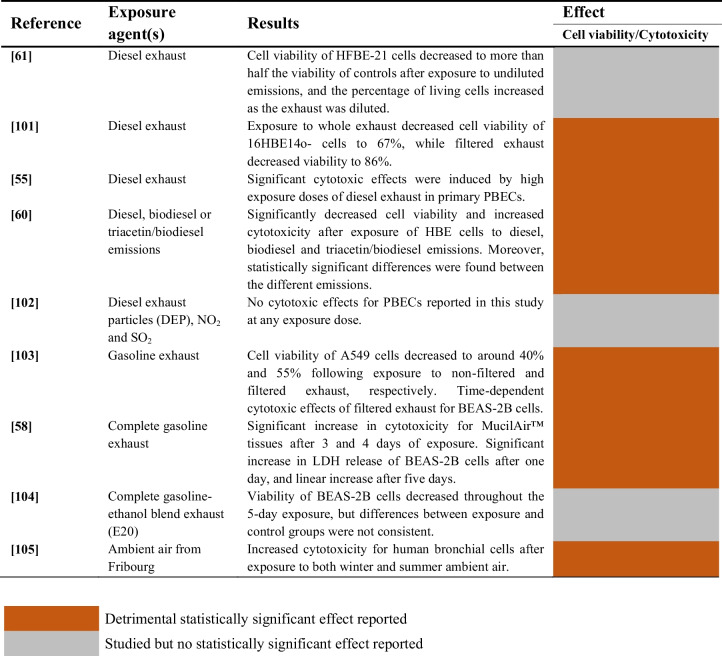
Cytotoxic effects of the mixture of aerosols and gases on airway epithelial cells

The cytotoxicity of diesel exhaust was assessed in 4 studies, and significant effects were reported in 3 studies [93–95]. Additionally, a notable decrease in cell viability was found in one study, but no statistical analyses were conducted [97].

The combined effect of diesel exhaust particles (DEP) with NO_2_ and SO_2_ was evaluated in one study, but no cytotoxic effects were found for primary bronchial epithelial cells (PBECs) at any exposure dose [96]. Cytotoxicity changed not only after exposure to diesel exhaust, but also to biodiesel and triacetin/biodiesel mixtures [95]. In fact, significantly different responses were reported in this study between the different emission compounds.

Gasoline exhaust emissions increased cytotoxicity in A549 cells [98], MucilAir™ tissues [99] and BEAS-2B cells [99, 100]. However, no significant differences between control and exposed BEAS-2B cell cultures were observed after exposure to gasoline-ethanol blend exhaust for five days [100]. On the other hand, one study from Bisig et al*.* reported significant cytotoxic effects of ambient air on human bronchial cells [102].

### Cellular Inflammation

Cellular inflammation is a complex defence biological process that involves the release of inflammatory mediators such as cytokines and chemokines, which can be quantified in the culture media using well-established methods [111], such as the Enzyme-linked immunosorbent assay (ELISA) [112].

The pro-inflammatory effects of the tested pollutants were assessed using the ELISA assay to measure the release of several pro-inflammatory markers such as IL-6, IL-8, IL-1α, IL-1β or MCP-1 in 28 studies [55, 56, 60, 63, 64, 66–73, 75, 76, 78–82, 88, 89, 92–96, 102]. Moreover, changes in the expression of several genes related to cellular inflammation like COX2 or PTGS2 were analysed using and RT-PCR in 15 studies [55, 58, 59, 61, 63, 68, 73, 75, 80, 82, 85, 90, 94, 96, 102]. Additionally, 7 studies utilized multiplex assays to measure panels of multiple cytokines and chemokines simultaneously [74, 77, 83, 84, 87, 91, 101].

#### Gaseous Pollutants

A summary of the studies evaluating the effects of gaseous pollutants on cellular inflammation of airway epithelial cells is provided in Table 7.

**Table 7:**
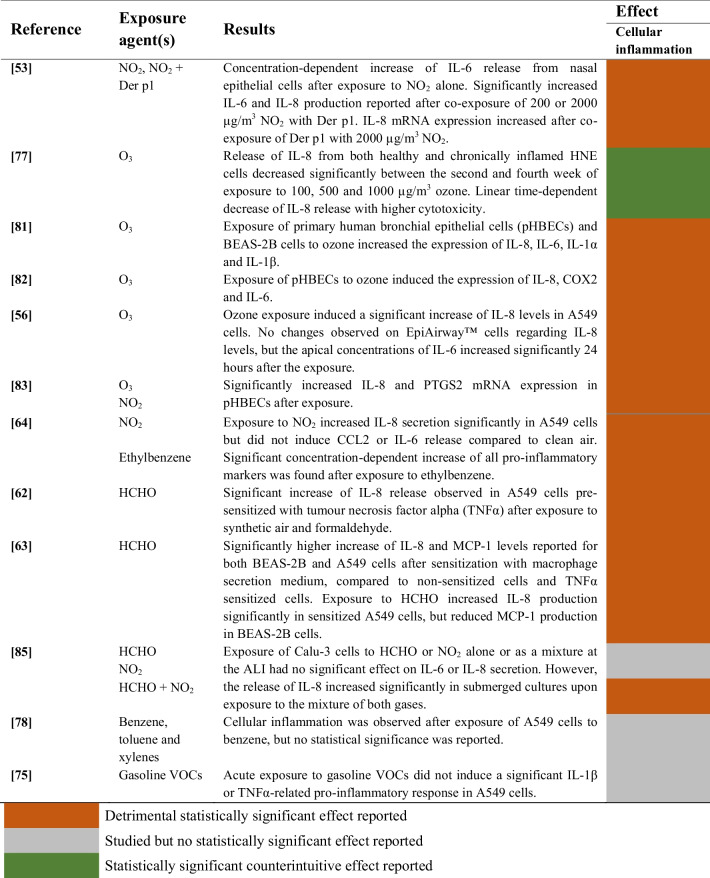
Effects of gaseous pollutant exposure on cellular inflammation of airway epithelial cells

##### Nitrogen Dioxide (NO_2_)

The impact of nitrogen dioxide on cellular inflammation was evaluated in 4 studies, and a significant increase of several pro-inflammatory cytokines (e.g., IL-6 and IL-8) was reported in 3 studies [55, 61, 68]. However, single exposure to NO_2_ at the air–liquid interface did not significantly increase the production of these cytokines in one study [66].

##### Ozone (O_3_)

The effects of ozone in cellular inflammation were assessed in 5 studies. After the exposures, statistically significant increases in cytokine release were reported in 4 studies [58–61]. However, a significant decrease of IL-8 release was reported in one study with human nasal epithelial cells, probably due to the decrease in cell viability [56].

##### Volatile Organic Compounds (VOCs)

Volatile OrganiFormaldehyde was tested in 3 studies, showing significant increases in the production of interleukins in 2 studies [63, 64]. Interestingly, both studies reported that the pro-inflammatory effect of formaldehyde was significantly enhanced by the previous sensitization of cells using TNFα.c Compounds (VOCs)

#### Aerosols and Particulate Matter (PM)

A summary of the studies evaluating the effects of aerosols and PM on cellular inflammation of airway epithelial cells is provided in Table 8.

**Table 8:**
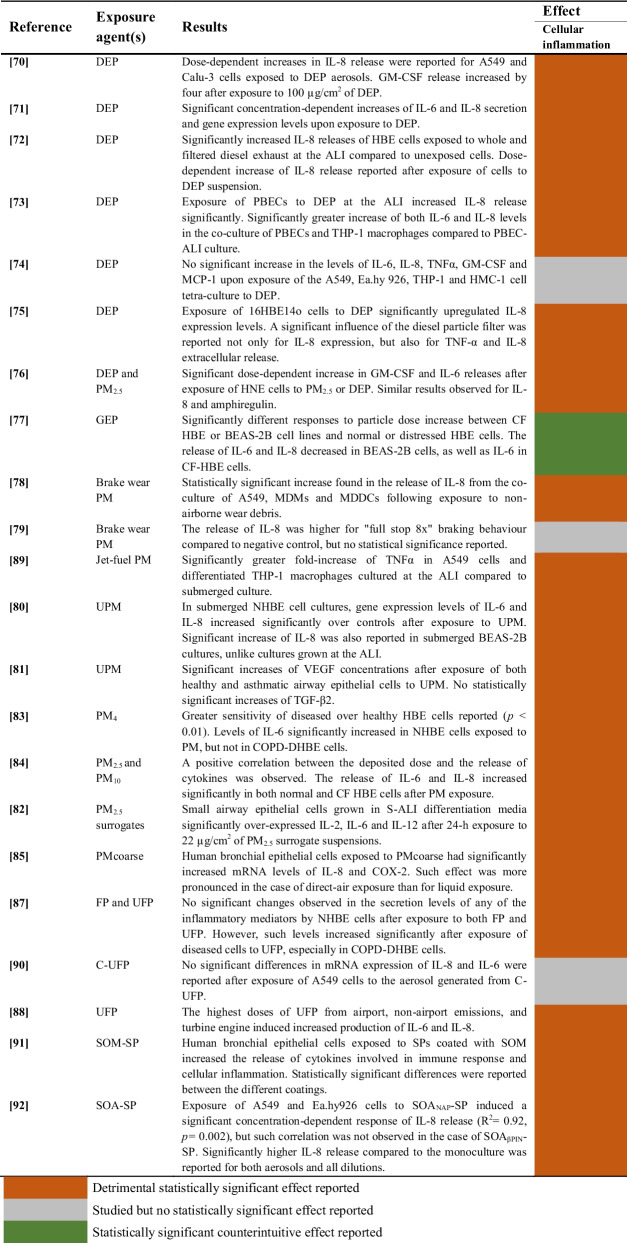
Effects of exposure to aerosols and particulate matter on cellular inflammation of airway epithelial cells

##### Diesel Exhaust Particles (DEP)

The effects of exposure to diesel exhaust particles on cellular inflammation were evaluated in 7 studies, and 6 of them reported significant effects [70–73, 75, 76]. However, one study did not find significant increases in the levels of the analysed cytokines [74]. On the other hand, particles from gasoline exhaust were tested in one study using bronchial epithelial cells, and significant decreases of IL-6 and IL-8 release were reported [77].

##### Particulate Matter (PM) and Secondary Aerosol from Soot Particles (SP)

Exposure to brake wear PM was tested in two studies, with significant effects on cellular inflammation found in one study [78] and no statistically significant results reported in the other [79]. Additionally, two studies found significantly increased levels of cytokine release in airway epithelial cells following exposure to urban particulate matter (UPM) [80, 81]. The effects of exposure to PM with different particle sizes were evaluated in 4 studies [82–85]. Significant upregulations of several pro-inflammatory markers (IL-2, IL-6, IL-8, IL-12 and COX-2) were reported in all these studies after exposure of different airway epithelial cell lines to PM_2.5_, PM_4_, PM_coarse_ or PM_10_. Pro-inflammatory effects of fine and ultrafine particles were assessed in 3 studies [87, 88, 90]. Two studies reported significantly increased levels of cytokines following exposure at high doses [88] or using diseased cellular models [87]. However, no statistically significant changes in IL-6 or IL-8 mRNA expression levels were found in one study using aerosol generated from ultrafine carbonaceous particles (C-UFP) [90].

Two studies assessed the effects of secondary aerosols from soot particles and found significantly induced release of interleukins in both human bronchial epithelial cells [91] and A549 cells [92].

#### Combined Aerosols and Gaseous Pollutants

Studies evaluating the impact of aerosols combined with gaseous pollutants on cellular inflammation of airway epithelial cells are summarized in Table 9.

**Table 9:**
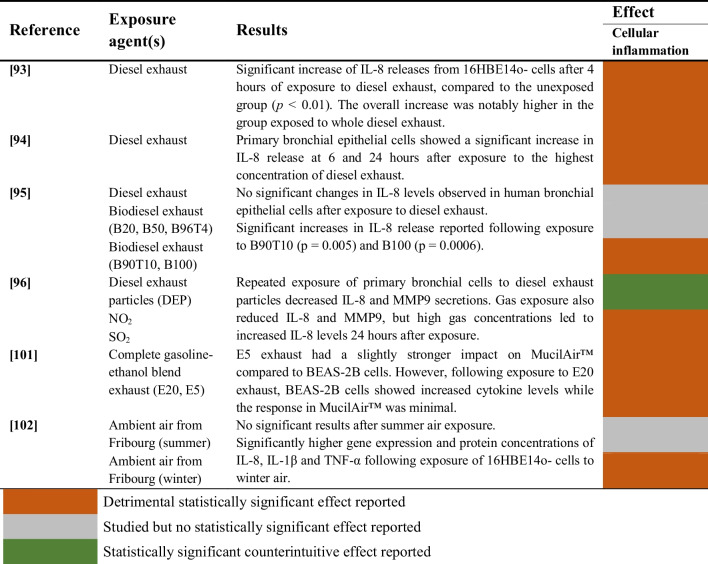
Effects of exposure to the mixture of aerosols and gases on cellular inflammation of airway epithelial cells

The effect of diesel exhaust on cellular inflammation was assessed in 3 studies [93–95]. Two studies found significant increases in cytokine release [93, 94], whereas one study reported no significant changes in IL-8 levels after the exposure [95]. Additionally, IL-8 and MMP9 secretions significantly decreased in one study after exposure to diesel exhaust particles combined with NO_2_ and SO_2_ [96].

On the other hand, exposure to gasoline-ethanol blends showed significantly increased cytokine levels for two different cell models in one study [101]. Finally, one study assessed the effect of ambient air in summer and winter, reporting significantly higher cellular inflammation after exposure to winter air [102].

### Genotoxicity

Genotoxicity refers to the capability of an agent to damage the genetic material within a cell, leading to mutations or other genetic alterations [113, 114] causing damage that may lead to adverse health outcomes [115–118]. The single-cell gel electrophoresis assay (Comet assay) is often used for the detection of DNA damage and repair in cells both in vitro and in vivo [119, 120].

In this review, the genotoxic effects of tested pollutants were assessed by the Comet assay in 7 studies [43, 52–54, 67, 69, 92], while genes related to DNA damage were analyzed using RT-PCR in 5 studies [61, 65, 81, 83, 99]. Additionally, other studies developed human 60-mer microarray [87] and transcriptomic [100] analyses. Although changes in gene expression alone do not necessarily imply genotoxicity, this section includes those studies that showed evidence of DNA damage, epigenetic alterations, or activation of DNA repair pathways following exposure to air pollutants.

#### Gaseous Pollutants

Studies included in this review evaluating the genotoxicity induced by the exposure of human airway epithelial cells to gaseous pollutants are summarized in Table 10.

**Table 10:**
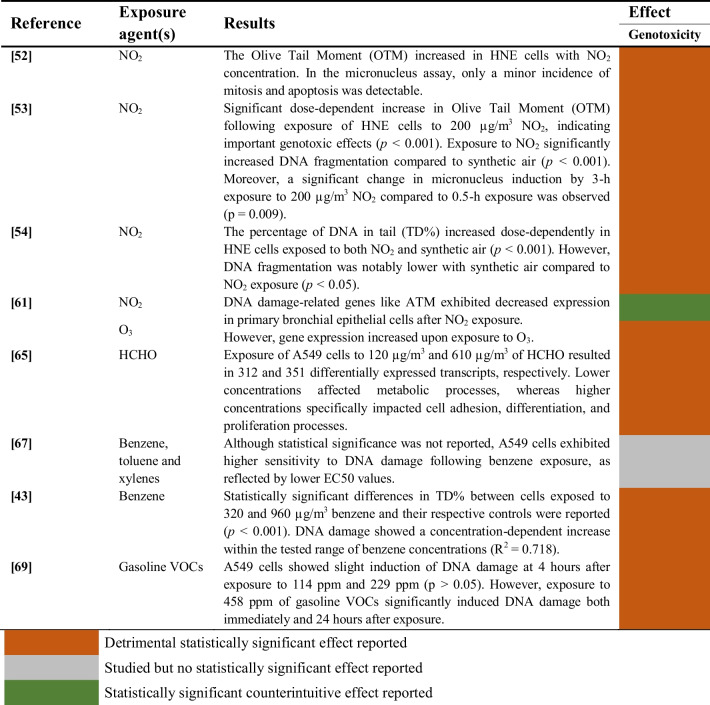
Genotoxic effects of gaseous pollutant exposure on human airway epithelial cells

The genotoxic effects induced by exposure to nitrogen dioxide were investigated in 4 studies [52–54, 61]. Three studies found a significant association between exposure to NO_2_ and increased DNA damage [52–54], whereas another study reported the downregulation of genes related to DNA damage such as ATM [61]. Conversely, an increased expression of these genes was observed in the same study after exposure to ozone.

Exposure to formaldehyde was genotoxic for A549 cells in one study, particularly at the highest concentrations [65]. In the case of benzene and other VOCs (BTEX), some effect on DNA damage was reported in one study [67], although not statistically significant. However, another study observed a significant concentration-dependent increase of DNA damage in A549 cells after exposure to benzene [43]. Similarly, exposure to gasoline VOCs significantly increased DNA damage in one study [69].

#### Aerosols and Particulate Matter (PM)

Studies evaluating the genotoxicity induced by the exposure of human airway epithelial cells to aerosols and particulate matter are summarized in Table 11.

**Table 11:**
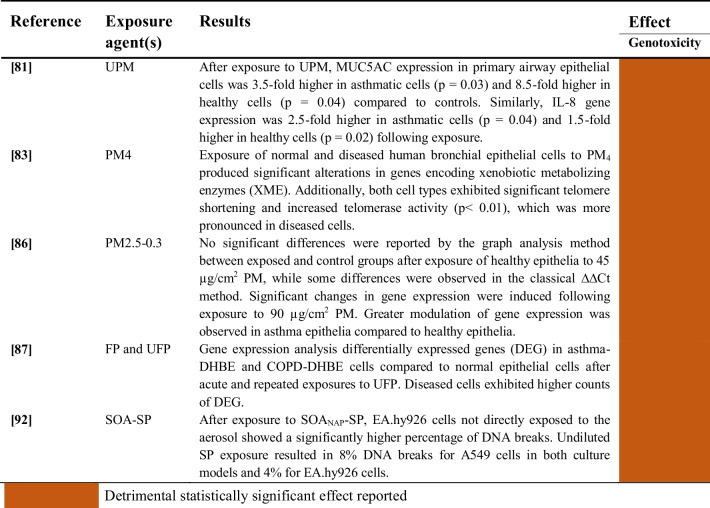
Genotoxic effects of exposure to aerosols and particulate matter on human airway epithelial cells

All studies outlined in Table 11 consistently show statistically significant genotoxic effects linked to exposure to aerosols and particulate matter of diverse sizes and chemical compositions across various human airway epithelial cell types.

#### Combined aerosols and gaseous pollutants

The two studies evaluating the genotoxic effects induced by the exposure of human airway epithelial cells to combined aerosols and gaseous pollutants are summarized in Table 12. Significant genotoxicity was induced by exposure to complete gasoline exhaust [99] and complete gasoline-ethanol blend exhaust [100] in both BEAS-2B cells and MucilAir ™ tissues.

**Table 12:**
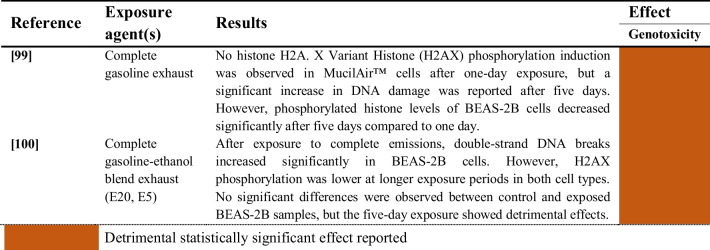
Genotoxic effects of combined exposure to aerosols and gaseous pollutants on human airway epithelial cells

### Oxidative Stress

Oxidative stress is a disturbance in the prooxidant-antioxidant balance in favour of the former [121], leading to potential oxidative damage [122, 123]. In the presence of an oxidative aggression, cells may initially react reinforcing antioxidant defences [124]. If the increased antioxidant response is not enough to counteract the oxidizing agents, then oxidative damage to macromolecules occurs. In case the oxidative aggression persists, living organisms finally exhaust their antioxidant agents and a situation of strong oxidative stress is established, in which a decrease in antioxidant defences is observed and the oxidative damage to macromolecules reaches its maximum levels [125].

The effect of exposure to pollutants on oxidative stress was assessed using the dichlorofluorescein (DCF) assay in 5 studies [43, 70, 76, 79, 98, 110], while changes in gene expression of markers involved in the cellular oxidative state, such as nuclear factor kappa B (NF-κB), Superoxide dismutase 1 (SOD1), Glutathione peroxidase (GPx) or Glutathione S-transferases (GST), were analysed through RT-PCR in 15 studies [59, 61, 68, 71, 73–75, 80, 84, 89, 90, 94–96, 102]. Additionally, two studies utilized the assay for quantitative determination of glutathione and glutathione disulfide (GSH/GSSG assay) [126] to measure the oxidative status of cells exposed to environmental pollutants [75, 78]. This assay is particularly relevant for the assessment of oxidative stress, since glutathione is arguably the most important intracellular non-enzymatic antioxidant that is produced in the body [127–129].

#### Gaseous Pollutants

Studies evaluating the induction of oxidative stress upon exposure of human airway epithelial cells to gaseous pollutants are summarized in Table 13

**Table 13:**
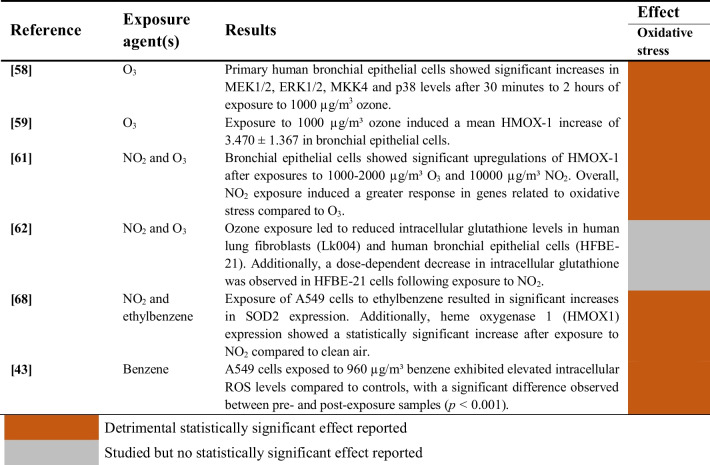
Oxidative stress induction of gaseous pollutants on human airway epithelial cells

The impact of nitrogen dioxide on oxidative stress was examined in 3 studies [61, 62, 68], while ozone-induced oxidative stress was evaluated in 4 studies [58, 59, 61, 62]. Moreover, the effects of exposure to ethylbenzene [68] and benzene [43] were assessed. Some of these studies show significantly increased levels of intracellular ROS [43] and protein kinases from the Mitogen-activated protein kinases (MAPKs) cascade [58], as well as a reduction of intracellular glutathione levels [62]. However, other studies reported the upregulation of antioxidant enzymes such as HMOX1 [59, 61, 68] and SOD2 [68], probably as a compensatory mechanism to the oxidative aggression.

#### Aerosols and Particulate Matter (PM)

Studies evaluating the induction of oxidative stress upon exposure of human airway epithelial cells to aerosols and particulate matter are summarized in Table 14.

**Table 14:**
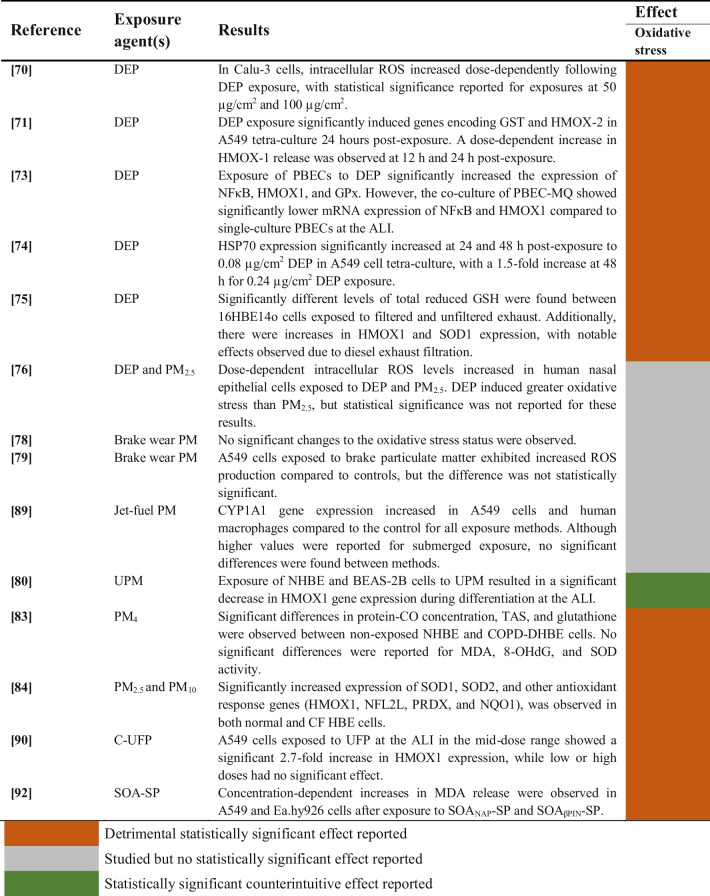
Oxidative stress induction of aerosols and particulate matter on human airway epithelial cells

##### Diesel Exhaust Particles (DEP)

The oxidative effects of DEP were assessed in 6 studies [70, 71, 73–76]. All these studies revealed increased intracellular ROS levels in human airway epithelial cells after exposure to DEP, and the expression of genes related to oxidative stress such as NFκB, HMOX1 or SOD1 was significantly altered as well. One study observed greater induction of oxidative stress of DEP compared to PM_2.5_, but the authors did not report any statistical analyses [76].

##### Particulate matter (PM) and Secondary Organic Aerosol from Soot Particles (SOA-SP)

Two studies investigated the effects of brake wear particulate matter on oxidative stress [78, 79], while another study assessed the impact of jet-fuel [89]. Despite a trend towards an increase in the oxidative stress response post-exposure, none of these studies reported statistically significant results. On the other hand, one study evaluating exposure to urban particulate matter (UPM) found a significant downregulation of HMOX1 after differentiation at the ALI [80].

Other studies found statistically significant associations between particulate matter or aerosol exposures and increased oxidative stress [83, 84, 90, 92]. These included not only alteration of antioxidants genes like SOD2 [84] or HMOX1 [84, 90], but also markers of oxidative DNA damage (8-OHdG) [83] and lipid peroxidation (MDA) [83, 92]. Moreover, oxidative damage of PM_4_ was reported in one study based on statistically significant increases of protein-CO concentration and glutathione oxidation in HBE cells, as well as significant decreases in total antioxidant status (TAS) [83].

### Combined Aerosols and Gaseous Pollutants

Studies evaluating the induction of oxidative stress following exposure of human airway epithelial cells to aerosols and particulate matter are summarized in Table 15.

**Table 15:**
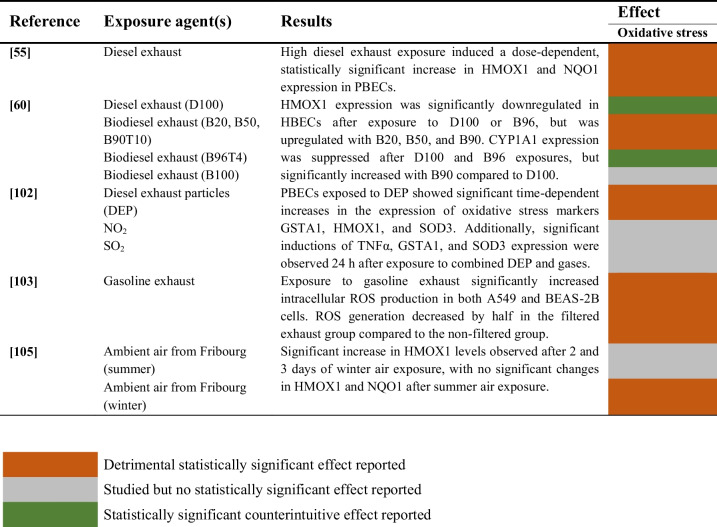
Oxidative stress induction of combined aerosols and gases on human airway epithelial cells

The induction of oxidative stress by diesel exhaust was evaluated in two studies [94, 95]. One study reported a dose-dependent statistically significant increase in HMOX1 and NQO1 expression [94]. Another study observed a downregulation of HMOX1 after exposure to D100 and B96, but this gene was upregulated following exposure to other mixtures of biodiesel exhaust, including B20, B50 and B90 [95]. On the other hand, a multi-cellular lung model exposed to winter ambient air showed increased levels of HMOX1 in one study [102]. In a different study, gasoline exhaust was found to increase intracellular ROS production significantly [98].

### Risk of Bias Assessment

The evaluation of the risk of bias is summarised in Table 16 (individual assessments in Supplementary Information). Nearly all studies included in this review were classified as Tier 1, since they had “definitely low” (+ +) or “probably low” (+) risk of bias for all the key aspects and other criteria selected. Only four studies [62, 75, 97, 102] were categorized as Tier 2 due to a “probably high” (** −**) or “definitely high” (**− −**) risk of bias in some key aspects. These included the lack of appropriate statistical approaches in three studies [62, 75, 97] and evidence of inadequate randomization of administered dose between study groups in one study [102]. Findings from all studies were integrated in the review, since no studies were classified as Tier 3.

**Table 16:**
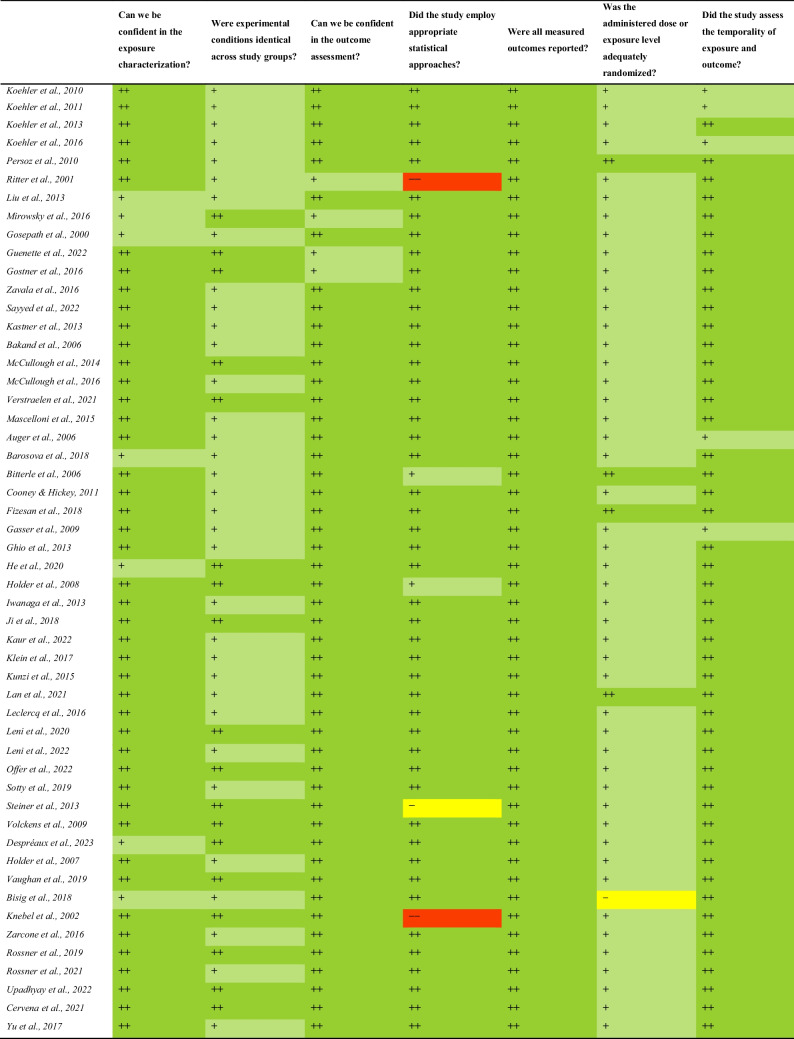
Summary of the risk of bias assessment of all studies included in the literature review

## Discussion

**Fig. 5:**
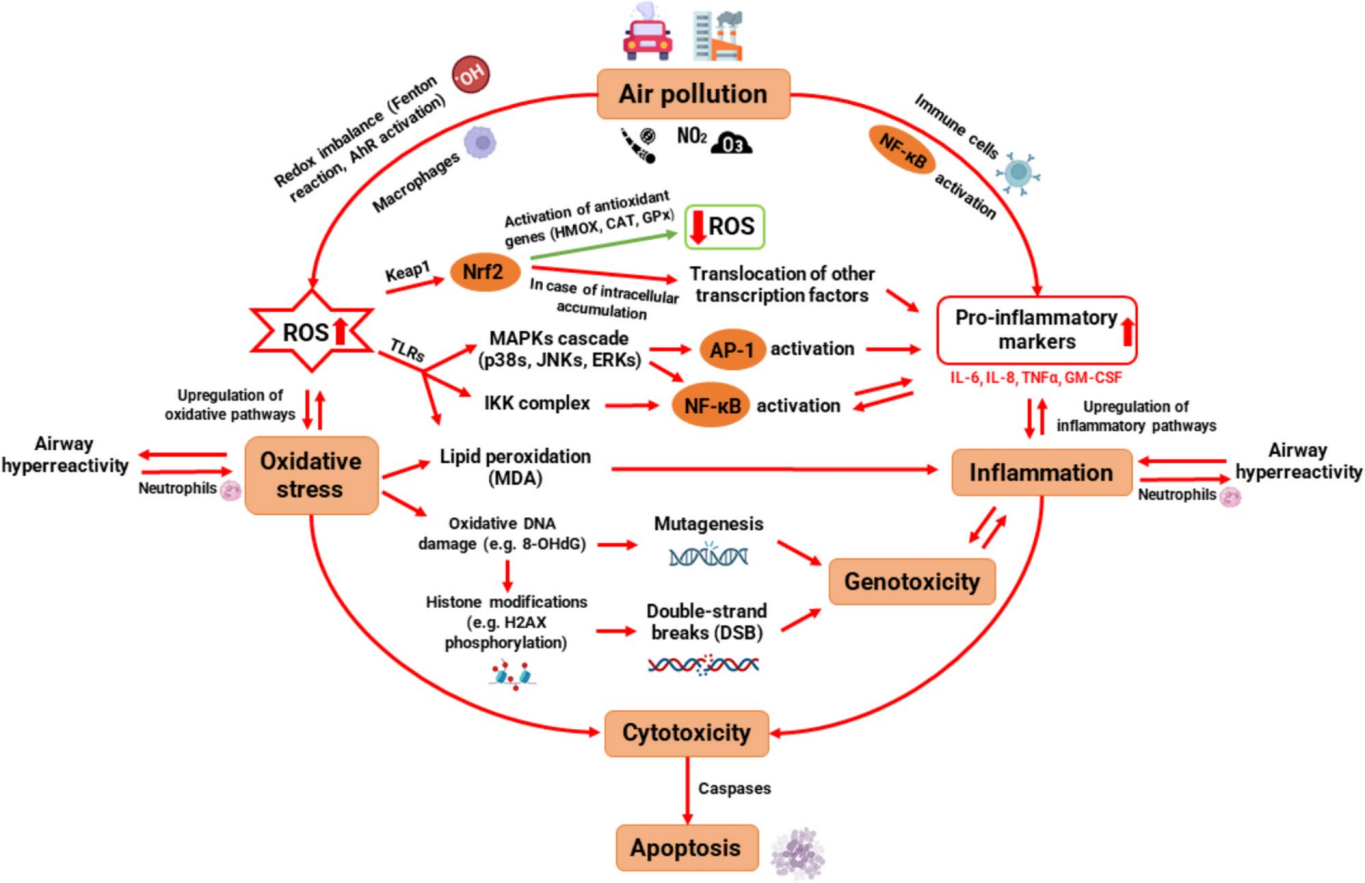
Schematic representation of several molecular pathways involved in the biologic responses induced by air pollution in human airway epithelial cells. AhR = aryl hydrocarbon receptor; ROS = reactive oxygen species; Keap1 = kelch-like ECH-associated protein 1; Nrf2 = nuclear factor erythroid 2–related factor 2; SOD = superoxide dismutase; CAT = catalase; GPx = glutathione peroxidase; TLRs = toll-like receptors; MAPKs = mitogen-activated protein kinases; ERKs = extracellular signal-regulated kinases; JNKs = c-Jun N-terminal kinases; p38s = p38 mitogen-activated protein kinases; AP-1 = activator protein 1; IKK = inhibitor of nuclear factor-κB (IκB) kinase; NF-κB = nuclear factor kappa B; MDA = malondialdehyde; 8-OHdG = 8-hydroxy-2'-deoxyguanosine; H2AX = H2A histone family member X

In this literature review, the impact of air pollution on human health was analysed by assessing the evidence of various in vitro toxicological studies using air–liquid interface (ALI) systems. These studies involved different combinations of air pollutants, exposure conditions and experimental designs using human airway cellular models, which represent the first point of contact of the air pollutants with the body. Such models included lung epithelial cell lines such as BEAS-2B, 16HBE14o or Calu-3, which reflect the bronchial epithelium, and A549 as model of alveolar epithelium. Results indicate that both acute and prolonged exposure to air pollution induce oxidative, inflammatory, and genotoxic responses in airway epithelial cells. Despite the differing physicochemical characteristics of air pollutants, evidence suggests that cells often respond within a common framework to the oxidative aggression. These responses are interconnected by many different pathways and often trigger cell death pathways like apoptosis after an enhanced cytotoxic response, as shown in Fig. 5. However, not all pollutants might trigger the same biological pathways, despite being interconnected. Aerosols might trigger the release of pro-inflammatory markers through phagocytosis [130]. On the other hand, the chemical composition of the aerosol might trigger oxidative stress, leading to inflammation, if not properly resolved [131]. Metals might increase oxidative stress through the Fenton reaction [132], whilst PAHs might do so through the AhR activation pathway [133]. Thus, not all pollutants might activate the same pathways, nor elicit the same effects.

Air pollutants increase the production of reactive oxygen species and pro-inflammatory markers through the activation of the immune system and redox imbalance. This leads to the upregulation of inflammatory and oxidative pathways, inducing airway hyperreactivity. Oxidative stress triggers several molecular pathways that enhance the inflammatory response through the activation of key transcription factors, including nuclear factor erythroid 2–related factor 2 (Nrf2), activator protein 1 (AP-1) and NF-κB. At the same time, lipid peroxidation and oxidative DNA damage induce genotoxic effects through mutagenesis and double-strand breaks, and both lipid peroxidation and genotoxicity can induce inflammation. These inflammatory and oxidative pathways increase the cytotoxic response, triggering regulated cell death pathways like apoptosis through the activity of caspases.

### The Oxidative Stress State Induced by ROS Overproduction and Redox Imbalance

**Fig. 6:**
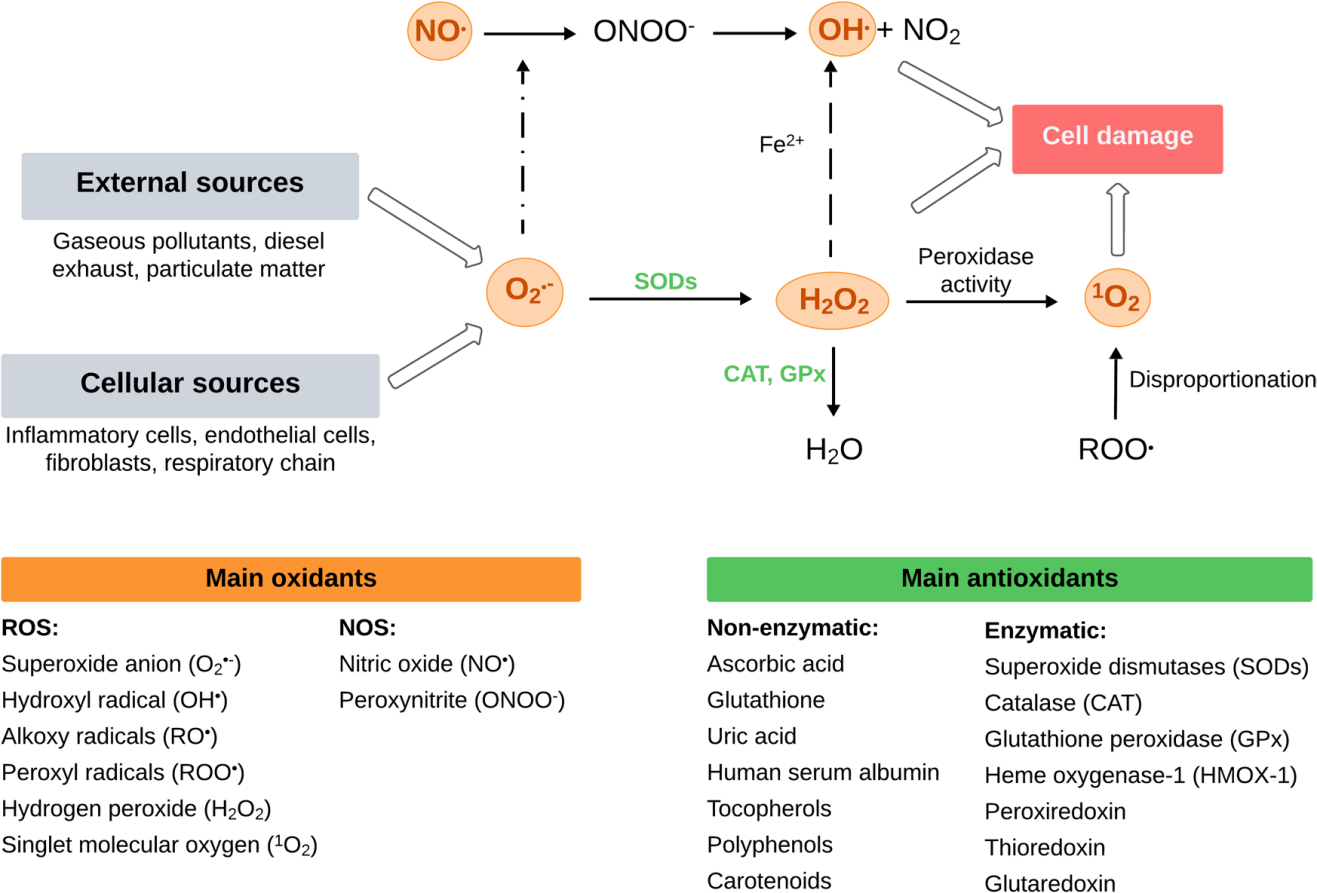
Cellular damage induced by generation of reactive oxygen species and antioxidant defense system. Adapted from Rahman et al. 2006 [136]

Upon inhalation, pollutants can cause lung damage due to oxidative stress by acting directly on the production of free ROS [134], or indirectly by inducing inflammation [19]. Direct induction of ROS can result from redox imbalance via Fenton reactions and activation of the aryl hydrocarbon receptor (AhR) (Fig. 6). Transition metals from particulate matter generate hydroxyl radicals and other ROS through Fenton reactions, increasing oxidative stress by the balance disruption between ROS production and antioxidant defences [135].

As shown in Fig. 6, ROS generated from oxidant air pollutants and cellular metabolism contribute to an increased oxidative stress state in the lungs [136]. First, superoxide (O_2_^•−^) and hydrogen peroxide (H_2_O_2_) are formed in vivo, and H_2_O_2_ is also originated through enzymatic and non-enzymatic superoxide dismutation [137]. Moreover, the hydroxyl radical (OH^•^) can be produced not only from H_2_O_2_ but also via the reaction of superoxide with nitric oxide (NO^•^) to produce peroxynitrite (ONOO^−^), which decomposes to form nitrogen dioxide and OH^•^. In fact, this hydroxyl radical is considered the most harmful and reactive oxidant species [138]. Some other relevant ROS are alkoxy and peroxyl radicals, which are mostly derived from lipids in biological systems [139]. Furthermore, singlet molecular oxygen (^1^O_2_) often arises from sunlight in the presence of molecular oxygen but may also originate from peroxidase activities in biological processes and the disproportionation of organic peroxyl radicals (ROO^•^) [140]. In fact, singlet oxygen is involved in the oxidation of lipids, nucleic acids, and proteins, and promotes detrimental processes such as lipid peroxidation, membrane damage, and cell death [140].

Oxidative stress is counteracted by an increase in the concentrations of antioxidant compounds to prevent oxidative damage to macromolecules. From a biochemical perspective, two classes of antioxidants are defined: enzymatic antioxidants (e.g., superoxide dismutases, glutathione peroxidase or catalase) and non-enzymatic like ascorbic acid, uric acid or glutathione (Fig. 6).

Main findings reported in this review regarding the induction of oxidative stress are consistent with the oxidant-antioxidant interplay described above. For instance, exposure of 16HBE14o- cells to diesel exhaust increased glutathione (GSH) oxidation in one study from this review [75]. Interestingly, diesel exhaust filtration reduced both GSH oxidation and IL-8 release, probably due to the lower concentrations of metals and organic compounds in the filtered exhaust that reduced its oxidative potential. In another study, exposure to DEP increased IL-8 release and ROS generation in Calu-3 cells [70], which is consistent with findings of other toxicological studies on lung epithelial cells [141–143]. At the same time, the oxidative aggression induced by air pollution described above can finally deplete cellular antioxidants faster than they are produced, further contributing to a redox imbalance [144]. However, several studies included in this review report significant upregulations of antioxidant enzymes such as HMOX1, SOD1 or SOD2 following exposure of airway epithelial cells to gases [59, 61, 68], particulate matter [71, 73, 75, 83, 84] and a combination of both [96]. Such increase of antioxidant gene expression might be explained by a temporary adaptive response to the oxidative aggression from air pollutants, as has been previously reported in several in vitro and in vivo studies [145–147]. However, it would be expected that these antioxidant levels decay as the oxidative aggression persists.

Ghio et al*.* argue that differentiation of bronchial epithelial cells at the ALI is associated with a diminished biological response from exposure to urban particulate matter (UPM), as there is a significant decrease in the gene expression of HMOX1 and IL-8 [80]. However, the authors did not assess the variation of any marker of oxidative damage, and intracellular ROS levels were not analysed either. These assessments would be useful to draw proper conclusions about the impact of differentiation on the potential oxidative stress state of the cell.

In the case of gaseous pollutants, a higher induction of antioxidant response was reported in this review after exposure to NO_2_ compared to O_3_, based on the higher upregulation of HMOX1 in primary HBE cells a mechanism of compensation to the oxidative aggression [61]. It has been reported that NO_2_ deposits mainly in the terminal bronchioles, whereas O_3_ deposits in the proximal airways [148, 149]. However, both gases interact with components from the epithelial lining fluid (ELF) barrier [150–152]. This might be a potential mechanism for toxicity as well, but such interactions are almost impossible to reproduce in vitro.

Other common air pollutants like polycyclic aromatic hydrocarbons (PAHs) are able to activate the AhR, leading to increased expression of cytochrome P450 enzymes like CYP1A1 that can generate excessive ROS during metabolism [153, 154]. This upregulation of CYP1A1 induced by air pollution was reported in two studies included in this review after exposure to biodiesel exhaust [95] and jet-fuel particulate matter [89].

### The Interplay Between NF-κB and Nrf2 and the Three-tiered Oxidative Stress Response to Air Pollution Exposure

Nuclear factor-κB (NF-κB) are a family of inducible transcription factors that regulate a large array of genes involved in different processes of the immune and inflammatory responses [155]. The ROS-mediated activation of the canonical NF-κB signalling pathway [156] has been described as a critical step in the inflammatory response to particulate matter [157, 158].

This transcription factor can be triggered by the activation of the IKK complex [159] or the MAPKs cascade [160], both induced by ROS overproduction (Fig. 6). Additionally, intracellular accumulation of ROS induces the translocation of NF-κB and other transcription factors, leading to an increased production of pro-inflammatory mediators and a final response of pulmonary and systemic inflammation [161]. Evidence from one study included in this review supports these observations, as Ji et al*.* identified significantly increased expression of NF-κB after exposure of primary bronchial epithelial cells to DEP, which induced inflammatory effects by the upregulation of IL-8 and TNFα [73]. On the other hand, Fig. 5 illustrates the activation of the nuclear factor erythroid 2-related factor 2 (Nrf2) through ROS-mediated modification of kelch-like ECH-associated protein 1 (Keap1). It is important to highlight that there is a complex interplay between NF-κB and Nrf2 through multiple molecular interactions, and any imbalance between these pathways is associated with a significant number of diseases [162]. These transcription factors have opposing effects on cellular processes, since NF-κB promotes inflammation and oxidative stress and Nrf2 activates antioxidant and cytoprotective genes [163]. Moreover, both NF-κB and Nrf2 are regulated by redox sensitive factors, and the absence of Nrf2 is associated with oxidative stress leading to increased cytokine production, since NF-κB is more readily activated in oxidative environments [164].

These complex molecular mechanisms mentioned above were discussed in two studies included in this review through the concept of the three-tiered hierarchical oxidative stress response to diesel exhaust particle exposure. Initially, Nrf2 translocation and antioxidant gene expression increase, as a defence mechanism to oxidative aggression from ROS production induced by DEP. As the production of ROS increases, an oxidative stress state is induced because oxidative aggression overcomes antioxidant defences, causing damage to macromolecules and pro-inflammatory effects. Finally, a cytotoxic response is often reflected by an increase in extracellular LDH release, likely due to membrane leakage or necrosis, and upregulation of pro-apoptotic genes (FAS and CASP7) [71, 74].

### Oxidative Damage to Macromolecules Induced by Reactive Oxygen Species (ROS) in Airway Epithelial Cells

As part of this oxidative aggression to macromolecules through the three-tiered oxidative stress concept, increased levels of ROS and oxidant pollutants can interact with polyunsaturated fatty acids (PUFAs) in cell membranes leading to lipid peroxidation. These interactions create lipid radicals and hydroperoxides, which decompose to form reactive aldehydes such as malondialdehyde (MDA) [165]. Subsequently, oxidized lipids can activate inflammatory pathways contributing to local and systemic inflammation [166, 167]. Offer et al*.* reported significant concentration-dependent increases of both MDA and IL-8 release in A549 and Ea.hy926 cells after exposure to secondary organic aerosols (SOA) from soot particles [92]. Moreover, after the exposure to SOA from soot particles and naphthalene, the authors observed a greater release of IL-8 and higher percentage of DNA breaks compared with exposure to SOA from soot particles and β-pinene. Even though no direct association was reported, it is reasonable to think that such increase in cytokine release could have been induced by all the ROS-dependent pathways described in Fig. 5 as well as from lipid peroxidation and overproduction of MDA.

Another outcome derived from the oxidative damage of air pollutants are the genotoxic effects, such as mutagenesis [168] and the formation of double strand breaks (DSB) [169]. Koehler et al*.* observed DNA fragmentation after NO_2_ exposure, possibly induced by the generation of reactive nitrogen species [52–54]. In these studies, exposure duration seems to have a strong influence on genotoxicity, though similar time-dependent effects were observed during prolonged exposure to synthetic air. This suggests that results on genotoxicity may be biased by cell dehydration effects.

Oxidative stress might also contribute to DSB indirectly through histone modifications that potentially increase the susceptibility of DNA to alterations in the chromatin structure [170]. However, some histone modifications like H2AX phosphorylation are considered sensitive biomarkers for genotoxicity, since they are part of the DNA damage response and help recruit repair factors to DSBs [169, 171, 172]. Some disparities about the effects of gasoline exhaust on H2AX phosphorylation have been reported in this review, which significantly increased after five days in one study [99] and decreased in another study at longer exposure periods [100]. This highlights the crucial role of the cell model and culture conditions used in toxicological tests for a better interpretation of the genotoxic effects.

### The Role of Alveolar Macrophages and Neutrophils in Airway Hyperreactivity and Inflammation

The mechanism inducing ROS production through the activation of alveolar macrophages (AMs) and their ability to phagocytose particulate matter in vivo and in vitro has been reported in several studies [173, 174]. These macrophages are activated, produce proinflammatory cytokines, and undergo apoptosis after being in contact with particulate matter [175]. Two studies included in this review reported statistically significant increases in the levels of proinflammatory cytokines in co-cultures of PBECs [73] or A549 cells [89] with THP-1 macrophages, following exposures to DEP and jet-fuel PM, respectively.

On the other hand, neutrophils are also able to stimulate the generation of ROS and induce airway hyperreactivity [176], which has been linked to many respiratory and cardiovascular diseases [134, 177, 178]. In fact, the synergy between Der p1 and NO_2_ observed by Koehler et al*.* [55] may be related with the upregulation of cytokines that are actively involved in early infiltration of circulatory polymorphonuclear neutrophils (PMNs) during acute pulmonary inflammation [179]. Thus, the inhibition of this mechanism may be a potential therapeutic target to alleviate the enhanced pro-inflammatory response in human nasal epithelial cells after acute exposures to NO_2_. A similar synergistic effect between air pollution and respiratory conditions was also reflected in the literature by the exacerbated inflammation in RV16-infected cells from continuous exposure to ambient levels of oxidant pollutants like NO_2_ and O_3_ [180]. As for volatile organic compounds (VOCs), formaldehyde is also known to exacerbate airway inflammation occurring in severe asthma by interacting with the respiratory epithelium, and even synergize the effects of other air pollutants and allergens [63–66]. Iwanaga et al*.* observed an increased inflammatory response in asthmatic cells compared to healthy cells following exposure to urban particulate matter (UPM), suggesting higher sensitivity of diseased cells [81].

Airway hyperreactivity and neutrophilic inflammation can be triggered by toll-like receptors (TLRs). These receptors are involved in different pathways, including lipid peroxidation [181], MAPKs cascade activation [182], or activation of the inhibitor of nuclear factor-κB (IKK) kinases complex [183], all leading to the upregulation of pro-inflammatory markers. Such inflammatory response induced by activation of TLRs was previously reported in mice after exposure to ozone [184]. McCullough et al*.* proposed a mechanism that is included in Fig. 6, in which O_3_-induced oxidative stress activates the epidermal growth factor receptor and cellular stress kinases, as a part of the MAPKs cascade that is initiated by the increase of ROS and activation of TLRs. The subsequent activation of the AP-1 transcription factor increases the levels of pro-inflammatory mediators in bronchial epithelial cells [58]. The role of TLRs was also investigated in one study based on a co-culture model of primary bronchial epithelial cells and macrophages exposed to DEP. Findings suggest that macrophages may reduce the sensitivity of PBECs during co-culture by downregulating TLR expression, but the strong impact of DEP might be able to mask this protective effect [73].

### Relationship Between Oxidative, Genotoxic and Inflammatory Responses Leading to Cytotoxicity

The genotoxic effects of air pollutants can also lead to an inflammatory response in cells through a complex interplay of oxidative stress [185], DNA damage [186] and epigenetic changes [187]. For instance, McCullough et al*.* linked inter-individual variability in proinflammatory and oxidative stress gene induction to the baseline levels of specific biomarkers of genotoxicity following ozone exposure [59]. However, the authors argue that one important limitation of their study is that they did not measure cytokine concentrations, as changes in gene expression might not directly correlate with increased cytokine levels. Despite this, some findings suggest that there is an important interaction between inflammatory processes and genotoxicity induced by exposure to environmental pollutants [186]. The genotoxic effects of benzene [43, 67] and gasoline VOCs [69] that were reported in this review align with studies showing irreversible DNA damage in A549 cells from benzene and toluene mixtures [188].

The inflammatory and oxidative pathways that are described above can ultimately lead to an enhanced cytotoxic response, which depends on the specific pollutants, exposure levels, and individual susceptibility factors. According to the findings of this review, cytotoxicity increased significantly after exposure to gaseous pollutants like nitrogen dioxide [51, 68], ozone [56, 60] and VOCs [67–69]. However, many tested concentrations greatly exceed typical annual mean levels found in urban areas, and factors such as flow rate and humidity may lead to overestimation of the cytotoxic and pro-inflammatory effects. On the other hand, interpretation of the results of one study is challenging because exposure concentration could not be measured [67], and all studies evaluating the impact of VOCs focused on the A549 cell line.

As for traffic-related emissions, it has been reported that biodiesel exhaust induces greater biological effects than diesel exhaust in primary human bronchial epithelial cells. This is probably due to the higher proportion of organic components in biodiesel, which are considered the most toxic components [189]. This hypothesis is supported by the significant effects on cell viability, inflammation (IL-6 and IL-8), antioxidant production (HO-1) and xenobiotic metabolism (CYP1A1) that were reported by Vaughan et al*.* after exposure to biodiesel emissions [95]. Moreover, both dilution and filtration of biodiesel exhaust significantly mitigate its cytotoxicity, which is in agreement with the reduction of pro-inflammatory and oxidative stress response after DEP filtration discussed above [75]. On the other hand, elimination of volatile and semi-volatile organic compounds from the exhaust resulted in minimal differences in viability compared to the whole exhaust, but showed reduced IL-8 release. This implies PAHs and other organic compounds play a key role in the inflammation caused by diesel exhaust.

Our results are consistent with a review by Cho et al*.* [49]. They also identified oxidative stress, inflammation and genotoxicity as key mechanisms involved in PM_2.5_-induced disease progression in both in vitro and in vivo models.

### Air–liquid Interface (ALI) vs Traditional Toxicological in vitro Studies

Air–liquid interface (ALI) systems allow for a better representation of the lung microenvironment and direct exposure to both particulate and gaseous components [89]. Repeated exposures at the ALI significantly increase cellular stress [63, 190], which can be reduced using the submerged exposure method. However, in such case the toxicological response is influenced by a protective effect of culture medium [85]. The definition of particle dose delivered to the cells in submerged exposures is challenging, but also crucial for an accurate representation of the particle interaction with the cell [93]. In fact, particles must diffuse through the media to reach cells, thus reporting lower delivered doses than the administered dose in some cases [191]. In contrast, ALI models allow for more precise dosimetry, reducing the need for assumptions about particle deposition and properties [89].

On the other hand, changing from a submerged to an ALI culture might increase oxidative stress response [192], and make the cells more susceptible to the effects of the particle exposure. Air–liquid interface systems also exhibit greater sensitivity, offering a similar response to the conventional particle suspension exposure at lower doses [72]. Additionally, they can be particularly useful for the assessment of the synergistic between gases and particles [193].

Despite the limitations mentioned above, traditional in vitro toxicology studies are easier to implement with a lower cost, and they have established protocols and wider availability [194]. Moreover, these systems can be very useful for initial screening of large numbers of compounds [195]. Therefore, both ALI and submerged systems have their place in toxicological research. However, their use should be guided by the specific research question, technical feasibility, and regulatory context.

ALI models offer improved physiological relevance and exposure conditions for respiratory toxicology, and constitute a valuable bridge between conventional in vitro assays and in vivo studies. However, they still lack important features of the living lung, such as tissue architecture, systemic circulation, and immune interactions, which may influence the nature and extent of the observed responses. These limitations can affect the interpretation of health risks and molecular mechanisms, particularly for endpoints involving chronic effects, immune modulation, or multi-organ interactions. To achieve a more comprehensive understanding of pollutant effects, findings from in vitro models should ideally be complemented with studies using primary human airway cells in more complex settings. Likewise, studies characterising toxicological responses in vivo to air pollution exposures in animal models, as well as in both healthy and susceptible populations should be conducted.

### Strengths and Limitations

Toxicological studies using cellular models require specific thermal, humidity and airflow conditions that promote cell viability for successful results. For this reason, first studies employing ALI systems were focused only on very short-term exposures and low flow rates. However, these conditions may not adequately reflect the biological changes from acute or sub-acute exposure scenarios in vivo and delivered doses might be insufficient for exposure assessment. When comparing the studies included in this review it is crucial to consider differences in particle size fractions, pollutant sources, and exposure doses. These factors can significantly influence the cellular response and may confound the interpretation of apparent toxicity gradients observed in summary tables. Therefore, such comparisons should be contextualized within the specific exposure conditions of each study, which may limit the interpretation of some of the findings discussed.

The review focused on studies using immortalized cell lines to elucidate the mechanism underpinning the health effects associated with exposures to air pollutants. As discussed in a previous review by Lakhdar et al*.*, immortalized cell lines are easily accessible and can be used at high passages, but do not show normal differentiation into the various cell populations that comprise the airway epithelium in vivo [48]. This could make them suboptimal for the evaluation of the mechanistic effects of air pollution compared to primary epithelial cell cultures. Nonetheless, the results of the review shows consistent results as regards eliciting oxidative, inflammatory, and genotoxic responses despite examining different endpoints, air pollutants and cellular models.

As highlighted by Persoz et al*.*, suboptimal exposure conditions compromising the number of viable cells may lead to underestimation of the actual impact of the test substance when compared to controls and misinterpretation of some biological outcomes [63]. Additionally, biological functions and processes are often characterized under diseased stages or situations of stress, which might be an important limitation for testing the effects of low-dose exposures [65]. Thus, optimization and verification of cell culture characteristics should be key factors to avoid overestimating the single effects of pollutants [57]. Despite this, Kunzi et al*.* argue that most in vitro studies are greatly over-dosed compared to the levels of realistic inhalation exposure and significantly deviate from realistic modes of particle application and target tissue [196]. In fact, realistic internal deposition requires atmospherically relevant mass, number concentration and composition of atmospheric particles and, critically, incorporation of atmospheric ageing [77].

Some of the studies included in this review use primary cells for in vitro exposure assessments, which are regarded as the most physiologically relevant cell models due to their closest resemblance to the native tissue. However, the isolation procedure and culturing of these cells are complex, and their use for large scale experiments such as toxicant screening is limited by their heterogeneity, high inter-donor variability and limited life span [71]. The use of very few cell lines and a small sample size in ALI systems often results in interpersonal variability, which might be a limitation as well. Co-cultures are probably the most suitable cell models, as they allow for the analysis of more endpoints at the same time compared to monocultures [102, 197].

One possible limitation of this review is the difficulty in comparing the results of the identified studies due to the heterogeneity in study designs and the wide range of variables considered, such as exposure conditions, cell cultures used and outcomes assessed. On the other hand, this review provides an overview of the consistency of the effects observed despite the heterogeneity in the study designs. Consistency across settings and study designs is one of the features for causality highlighted by Bradford Hill [198]. Thus, the consistency of findings across such diverse studies strengthens the evidence on possible biological mechanisms underlying the health effects derived from air pollution.

On the other hand, evidence integrated in this literature review was evaluated based on the OHAT approach and adapted for in vitro air–liquid interface exposures. Only few studies had high or definitely high risk of bias (RoB) due to the lack of statistical analyses reported [62, 97], or the use of suboptimal statistical approaches [75]. However, one strength of most studies in this literature review is that all the evaluated criteria from the evidence reported had low or definitely low RoB. Additionally, these studies are based mainly on 3D cellular models, also known as organs-on-a-chip [199], thus making important contributions to minimize the use of animal models in toxicology.

This systematic literature review only includes articles in English published until March 11, 2024. Thus, any relevant scientific article published after this date or in a different language has not been included in this review. Moreover, it includes few studies focused on the assessment of exposure to ambient air, and the synergistic effect between particles and gases has not been thoroughly investigated.

One particular strength of this work is that, to the best of our knowledge, it is one of the first systematic reviews assessing the effects of gases, aerosols, and combinations of both types of environmental pollutants on human health using airway epithelial cells cultured on air–liquid interface systems.

## Conclusions and Future Perspectives

Taken together, nearly all studies included in this review indicate significant toxicological responses from air pollutants on human airway epithelial cells cultured at the air–liquid interface, which are particularly enhanced in diseased cells. Upon inhalation, the oxidative potential of gases, aerosols and particulate matter creates a redox imbalance that increases the production of reactive oxygen species (ROS). In response, the Nrf2 pathway is activated as a compensatory mechanism, increasing the production of antioxidant species to neutralize ROS. However, this is eventually overcome by the oxidative aggression of air pollutants creating a state of oxidative stress. At this stage, the release of pro-inflammatory markers is increased by the activation of immune cells and transcription factors such as NF-κB and AP-1. In parallel, oxidative damage to cellular macromolecules through processes such as lipid peroxidation and DNA strand breaks can trigger genotoxic effects, epigenetic changes, and inflammation. These events may ultimately lead to cytotoxicity and cell death, primarily via caspase activation.

Most of the existing literature has focused on the individual effects of gases or particulate matter, whereas few studies have assessed the synergistic effects of both types of airborne pollutants through repeated daily exposure. However, considering that, real-life inhalation involves simultaneous exposure to gases and particles, future research should prioritize experimental designs that reflect better these complex exposure scenarios. Additionally, it would be important to consider factors influencing cellular sensitivity, such as culture duration prior to exposure, epithelial-macrophage interactions, and interindividual variability between cell donors. More specifically, it could be useful to elucidate the potential role of lipid biochemical pathways as part of the cell’s response to formaldehyde, as well as the ability of diesel particle filters in combination with diesel oxidation catalysts to reduce oxidative and inflammatory responses induced by DEP.

Further development of ALI co-culture models is also needed to better mimic the structural and functional complexity of the airway epithelium [200, 201]. This includes the use of dynamic exposure systems capable of simulating prolonged and repeated exposures, as well as accounting for photochemical ageing of aerosols and particle deposition patterns. Such improvements would help bridge the gap between in vivo and in vitro approaches. However, as previously discussed by Lakhdar et al*.* [48], it is important to acknowledge that these cellular models cannot fully replicate critical features of living tissue, such as the presence of a functional immune system and organ-level interactions. These features are essential for accurately assessing complex biological responses that are seen in vivo after inhalation of gases and particles, hence representing an intrinsic restriction of ALI systems, common to submerged in vitro experiments.

The current review complements the insights offered by Lakhdar et al*.*, focused on senescence, mitochondrial damage, and autophagy, which were not extensively discussed in the present work. Instead, this review focuses on oxidative stress as a central mechanism underlying pollutant-induced toxicity, which is discussed through the concept of *three-tiered oxidative stress* and the importance of oxidant-antioxidant balance.

In conclusion, this review contributes to the existing body of evidence regarding the molecular and biochemical mechanisms through which air pollution impacts human health, with particular emphasis on oxidative stress.

## Key References

- Kampa, M. and E. Castanas, Human health effects of air pollution. Environ Pollut, 2008. 151(2): p. 362–7.
  - ⚬ A brief review of the effects of air pollutants on different organs and systems, as well as the cellular mechanisms involved in their adverse effects.
- Yang, Y., et al., Short-term and long-term exposures to fine particulate matter constituents and health: A systematic review and meta-analysis. Environ Pollut, 2019. 247: p. 874–882.
  - ⚬ This paper effectively reviews both short- and long-term health impact of toxic components from PM_2.5_, finding significant associations with all natural, cardiovascular mortality and morbidity.
- Chiusolo, M., et al., Short-Term Effects of Nitrogen Dioxide on Mortality and Susceptibility Factors in 10 Italian Cities: The EpiAir Study. Environ Health Perspect, 2011. 119(9): p. 1233–8.
  - ⚬ The study suggests significant effects of NO_2_ on natural, cardiac, and respiratory mortality, which were independent from those of PM_10_ and O_3_.
- Upadhyay, S. and L. Palmberg, Air–Liquid Interface: Relevant In Vitro Models for Investigating Air Pollutant-Induced Pulmonary Toxicity. Toxicol Sci, 2018. 164(1): p. 21–30.
  - ⚬ This review highlights the relevance of air–liquid interface systems compared to traditional in vitro and in vivo toxicological studies.
- Rahman, I., S.K. Biswas, and A. Kode, Oxidant and antioxidant balance in the airways and airway diseases. Eur J Pharmacol, 2006. 533(1–3): p. 222–39.
  - ⚬ The manuscript extensively reviews the molecular mechanisms involved in the oxidative stress state of airway epithelium, as well as the most common inhaled oxidants and antioxidant species in the lungs.

## Supplementary Information

Below is the link to the electronic supplementary material.

Supplementary file1 (DOCX 35.4 KB)

Supplementary file2 (DOCX 16 KB)

Supplementary file3 (XLSX 111 KB)

Supplementary file4 (DOCX 209 KB)

Supplementary file5 (DOCX 205 KB)

## Data Availability

No datasets were generated or analysed during the current study.
