# Supplementary material for: Toxicological Effects of Air Pollutants on Human Airway Cell Models Using Air–liquid Interface Systems: A Systematic Review
Source: Curr Environ Health Rep. 2025 Jul 28;12(1):26. doi: 10.1007/s40572-025-00491-w (PMC12304069; doi:10.1007/s40572-025-00491-w)
Supplement: Supplementary file 2 — Supplementary file2 (DOCX 16 KB) [file 40572_2025_491_MOESM2_ESM.docx]

**Literature search strategy**

**Search terms**

1. Air-liquid cellular model or Air-liquid interface culture

2. Human respiratory tract or human respiratory cellular model

3. Particulate matter or PM or PM2.5 or PM10 or PMcoarse

4. Nitrogen dioxide

5. Carbon monoxide

6. Sulfur dioxide

7. Ozone

8. Aerosols or aerosol

9. Air pollution or air pollutants

10. 3 or 4 or 5 or 6 or 7 or 8 or 9

11. Inflammation

12. Oxidative stress

13. Cytotoxicity

14. Gene expression

15. Proliferation

16. 1 and 2 and 10 and 11

17. 1 and 2 and 10 and 12

18. 1 and 2 and 10 and 13

19. 1 and 2 and 10 and 14

20. 1 and 2 and 10 and 15

21. 16 or 17 or 18 or 19 or 20

**Search strings**

**Pubmed**

((((((Air-liquid cellular model OR Air-liquid interface culture) AND (Human respiratory tract OR human respiratory cellular model)) AND ((Particulate matter OR PM OR PM2.5 OR PM10 OR PMcoarse) OR (Nitrogen dioxide) OR (Carbon monoxide) OR (Sulfur dioxide) OR (Ozone) OR (Aerosols OR aerosol) OR (Air pollution OR air pollutants) AND (Inflammation)))

OR

(((Air-liquid cellular model OR Air-liquid interface culture) AND (Human respiratory tract OR human respiratory cellular model)) AND ((Particulate matter OR PM OR PM2.5 OR PM10 OR PMcoarse) OR (Nitrogen dioxide) OR (Carbon monoxide) OR (Sulfur dioxide) OR (Ozone) OR (Aerosols OR aerosol) OR (Air pollution OR air pollutants) AND (Oxidative stress))))

OR

(((Air-liquid cellular model OR Air-liquid interface culture) AND (Human respiratory tract OR human respiratory cellular model)) AND ((Particulate matter OR PM OR PM2.5 OR PM10 OR PMcoarse) OR (Nitrogen dioxide) OR (Carbon monoxide) OR (Sulfur dioxide) OR (Ozone) OR (Aerosols OR aerosol) OR (Air pollution OR air pollutants) AND (Cytotoxicity))))

OR

(((Air-liquid cellular model OR Air-liquid interface culture) AND (Human respiratory tract OR human respiratory cellular model)) AND ((Particulate matter OR PM OR PM2.5 OR PM10 OR PMcoarse) OR (Nitrogen dioxide) OR (Carbon monoxide) OR (Sulfur dioxide) OR (Ozone) OR (Aerosols OR aerosol) OR (Air pollution OR air pollutants) AND (Gene expression))))

OR

(((Air-liquid cellular model OR Air-liquid interface culture) AND (Human respiratory tract OR human respiratory cellular model)) AND ((Particulate matter OR PM OR PM2.5 OR PM10 OR PMcoarse) OR (Nitrogen dioxide) OR (Carbon monoxide) OR (Sulfur dioxide) OR (Ozone) OR (Aerosols OR aerosol) OR (Air pollution OR air pollutants) AND (Proliferation)))

**Scopus**

TITLE-ABS-KEY ( ( ( ( ( ( ( air-liquid AND cellular AND model OR air-liquid AND interface AND culture ) AND ( human AND respiratory AND tract OR human AND respiratory AND cellular AND model ) ) AND ( ( particulate AND matter OR pm OR pm2.5 OR pm10 OR pmcoarse ) OR ( nitrogen AND dioxide ) OR ( carbon AND monoxide ) OR ( sulfur AND dioxide ) OR ( ozone ) OR ( aerosols OR aerosol ) OR ( air AND pollution OR air AND pollutants ) AND ( inflammation ) ) ) OR ( ( ( air-liquid AND cellular AND model OR air-liquid AND interface AND culture ) AND ( human AND respiratory AND tract OR human AND respiratory AND cellular AND model ) ) AND ( ( particulate AND matter OR pm OR pm2.5 OR pm10 OR pmcoarse ) OR ( nitrogen AND dioxide ) OR ( carbon AND monoxide ) OR ( sulfur AND dioxide ) OR ( ozone ) OR ( aerosols OR aerosol ) OR ( air AND pollution OR air AND pollutants ) AND ( oxidative AND stress ) ) ) ) OR ( ( ( air-liquid AND cellular AND model OR air-liquid AND interface AND culture ) AND ( human AND respiratory AND tract OR human AND respiratory AND cellular AND model ) ) AND ( ( particulate AND matter OR pm OR pm2.5 OR pm10 OR pmcoarse ) OR ( nitrogen AND dioxide ) OR ( carbon AND monoxide ) OR ( sulfur AND dioxide ) OR ( ozone ) OR ( aerosols OR aerosol ) OR ( air AND pollution OR air AND pollutants ) AND ( cytotoxicity ) ) ) ) OR ( ( ( air-liquid AND cellular AND model OR air-liquid AND interface AND culture ) AND ( human AND respiratory AND tract OR human AND respiratory AND cellular AND model ) ) AND ( ( particulate AND matter OR pm OR pm2.5 OR pm10 OR pmcoarse ) OR ( nitrogen AND dioxide ) OR ( carbon AND monoxide ) OR ( sulfur AND dioxide ) OR ( ozone ) OR ( aerosols OR aerosol ) OR ( air AND pollution OR air AND pollutants ) AND ( gene AND expression ) ) ) ) OR ( ( ( air-liquid AND cellular AND model OR air-liquid AND interface AND culture ) AND ( human AND respiratory AND tract OR human AND respiratory AND cellular AND model ) ) AND ( ( particulate AND matter OR pm OR pm2.5 OR pm10 OR pmcoarse ) OR ( nitrogen AND dioxide ) OR ( carbon AND monoxide ) OR ( sulfur AND dioxide ) OR ( ozone ) OR ( aerosols OR aerosol ) OR ( air AND pollution OR air AND pollutants ) AND ( proliferation ) ) ) )

**Web of science**

((((TS=(((((Air-liquid cellular model OR Air-liquid interface culture) AND (Human respiratory tract OR human respiratory cellular model)) AND ((Particulate matter OR PM OR PM2.5 OR PM10 OR PMcoarse) OR (Nitrogen dioxide) OR (Carbon monoxide) OR (Sulfur dioxide) OR (Ozone) OR (Aerosols OR aerosol) OR (Air pollution OR air pollutants) AND (Inflammation))) )) OR TS=((((((Air-liquid cellular model OR Air-liquid interface culture) AND (Human respiratory tract OR human respiratory cellular model)) AND ((Particulate matter OR PM OR PM2.5 OR PM10 OR PMcoarse) OR (Nitrogen dioxide) OR (Carbon monoxide) OR (Sulfur dioxide) OR (Ozone) OR (Aerosols OR aerosol) OR (Air pollution OR air pollutants) AND (Oxidative stress)))))) OR TS=((((((Air-liquid cellular model OR Air-liquid interface culture) AND (Human respiratory tract OR human respiratory cellular model)) AND ((Particulate matter OR PM OR PM2.5 OR PM10 OR PMcoarse) OR (Nitrogen dioxide) OR (Carbon monoxide) OR (Sulfur dioxide) OR (Ozone) OR (Aerosols OR aerosol) OR (Air pollution OR air pollutants) AND (Cytotoxicity)))))) OR TS=((((((Air-liquid cellular model OR Air-liquid interface culture) AND (Human respiratory tract OR human respiratory cellular model)) AND ((Particulate matter OR PM OR PM2.5 OR PM10 OR PMcoarse) OR (Nitrogen dioxide) OR (Carbon monoxide) OR (Sulfur dioxide) OR (Ozone) OR (Aerosols OR aerosol) OR (Air pollution OR air pollutants) AND (Gene expression)))))) OR TS=((((Air-liquid cellular model OR Air-liquid interface culture) AND (Human respiratory tract OR human respiratory cellular model)) AND ((Particulate matter OR PM OR PM2.5 OR PM10 OR PMcoarse) OR (Nitrogen dioxide) OR (Carbon monoxide) OR (Sulfur dioxide) OR (Ozone) OR (Aerosols OR aerosol) OR (Air pollution OR air pollutants) AND (Proliferation))) )))))
