# Supplementary material for: Toxicological Effects of Air Pollutants on Human Airway Cell Models Using Air–liquid Interface Systems: A Systematic Review
Source: Curr Environ Health Rep. 2025 Jul 28;12(1):26. doi: 10.1007/s40572-025-00491-w (PMC12304069; doi:10.1007/s40572-025-00491-w)
Supplement: Supplementary file 4 — Supplementary file4 (DOCX 209 KB) [file 40572_2025_491_MOESM4_ESM.docx]

**Description of studies included in the systematic review by outcomes and air pollutants**

1. **Cell viability and cytotoxicity**
   1. **Gaseous pollutants**
      1. *Nitrogen dioxide (NO_2_)*

Human lung fibroblasts (Lk004) and HFBE-21 epithelial cells were exposed to increasing concentrations of NO_2_ ranging from 150 to 2400 µg/m^3^ using a CULTEX ® exposure system ([Ritter et al., 2001](https://ujies-my.sharepoint.com/personal/onavarre_uji_es/Documents/Doctorado/2.%20Artículos/1.%20Systematic%20review%20ALI/Texto%20completo%20systematic%20review%20ALI%2031-5-2024%20(Óscar)_JMDS.docx#_ENREF_117)). After a 2-hour exposure, Lk004 cell number and viability decreased to 70-80% at 300 µg/m^3^ and 40-50% at 600 µg/m^3^, as measured by the WST-1 assay. Meanwhile, viability of HFBE-21 cells exhibited a dose-dependent decrease, reaching 50% after 1-hour exposure to 2400 µg/m^3^ NO_2_.

In a similar study, Bakand *et al.* assessed the effects of direct airborne exposure to NO_2_ at concentrations ranging from 5000 to 20000 µg/m^3^ on A549 cells and skin fibroblasts ([Bakand et al., 2006](https://ujies-my.sharepoint.com/personal/onavarre_uji_es/Documents/Doctorado/2.%20Artículos/1.%20Systematic%20review%20ALI/Texto%20completo%20systematic%20review%20ALI%2031-5-2024%20(Óscar)_JMDS.docx#_ENREF_8)). Cell viability and cytotoxicity were determined using three different assays: MTS assay, neutral red uptake (NRU) and measurement of the intracellular ATP content. The results indicated a significant reduction in cell viability for both cell types, even at the lowest concentration of 5000 µg/m^3^. Moreover, concentration-dependent decreases in cell viability were observed across the entire concentration range after 1-hour exposures (p< 0.05, Student’s t-test). At the highest concentration of 20000 µg/m^3^, cell viability dropped below 50%, reaching 49.4 ± 9.9% for A549 cells (NRU assay) and 46.15 ± 7.6% for skin fibroblasts (ATP assay). Dose-dependent cytotoxic effects of NO_2_ were observed in A549 cells following exposure to 10000 µg/m^3^ for durations ranging from 30 minutes to 2 hours. However, it is important to highlight that the authors found a significant reduction in cell viability as the flow rate of synthetic air was increased to 50 mL/min, which could potentially lead to overestimation of NO_2_ cytotoxicity. These results are consistent with those reporting a significant decrease in A549 cell viability to 67% (p< 0.001, ANOVA test compared to clean air) in the MTT assay and a 9% (p< 0.01, ANOVA) increase in mean cytotoxicity in the LDH assay after exposure to approximately 40000 µg/m^3^ NO_2_ for 4 hours ([Verstraelen et al., 2021](https://ujies-my.sharepoint.com/personal/onavarre_uji_es/Documents/Doctorado/2.%20Artículos/1.%20Systematic%20review%20ALI/Texto%20completo%20systematic%20review%20ALI%2031-5-2024%20(Óscar)_JMDS.docx#_ENREF_135)).

In the case of human nasal epithelial cells cultured at the air-liquid interface, exposure to NO_2_ at concentrations ranging from 20 to 20000 µg/m^3^ for 30 minutes was carried out in one study ([Koehler et al., 2010](https://ujies-my.sharepoint.com/personal/onavarre_uji_es/Documents/Doctorado/2.%20Artículos/1.%20Systematic%20review%20ALI/Texto%20completo%20systematic%20review%20ALI%2031-5-2024%20(Óscar)_JMDS.docx#_ENREF_69)). The results revealed that there was no decrease in cell viability, and no significant effects were observed on cell proliferation, apoptosis, membrane integrity, or necrosis. Subsequent studies with the same cell type showed that cell proliferation was not affected by the exposure duration, as both cell viability (trypan blue assay) and cytotoxicity (Caspase-3 assay) did not exhibit statistically significant changes ([Koehler et al., 2011](https://ujies-my.sharepoint.com/personal/onavarre_uji_es/Documents/Doctorado/2.%20Artículos/1.%20Systematic%20review%20ALI/Texto%20completo%20systematic%20review%20ALI%2031-5-2024%20(Óscar)_JMDS.docx#_ENREF_70); [Koehler et al., 2013](https://ujies-my.sharepoint.com/personal/onavarre_uji_es/Documents/Doctorado/2.%20Artículos/1.%20Systematic%20review%20ALI/Texto%20completo%20systematic%20review%20ALI%2031-5-2024%20(Óscar)_JMDS.docx#_ENREF_72)).

In a different study focused on Calu-3 cells, no significant changes in cell viability (MTT assay) or cytotoxicity (LDH assay) were reported after exposure to NO_2_ at concentrations ranging from 200 to 800 µg/m^3^ ([Kastner et al., 2013](https://ujies-my.sharepoint.com/personal/onavarre_uji_es/Documents/Doctorado/2.%20Artículos/1.%20Systematic%20review%20ALI/Texto%20completo%20systematic%20review%20ALI%2031-5-2024%20(Óscar)_JMDS.docx#_ENREF_61)).

- - 1. *Ozone (O_3_)*

A dose-dependent decrease in cell viability of human lung fibroblasts (Lk004) and human bronchial epithelial cells (HFBE-21) was observed in the WST-1 assay upon exposure to 400 and 1000 µg/m^3^ O_3_ ([Ritter et al., 2001](https://ujies-my.sharepoint.com/personal/onavarre_uji_es/Documents/Doctorado/2.%20Artículos/1.%20Systematic%20review%20ALI/Texto%20completo%20systematic%20review%20ALI%2031-5-2024%20(Óscar)_JMDS.docx#_ENREF_117)). Such detrimental effect was also found in A549 cells exposed to 400 µg/m^3^ O_3_ for 2 hours, reporting a significant decrease in cell viability as determined by the resazurin assay ([Guenette et al., 2022](https://ujies-my.sharepoint.com/personal/onavarre_uji_es/Documents/Doctorado/2.%20Artículos/1.%20Systematic%20review%20ALI/Texto%20completo%20systematic%20review%20ALI%2031-5-2024%20(Óscar)_JMDS.docx#_ENREF_44)). Moreover, Zavala *et al.* identified significant cytotoxic effects of ozone in A549 cells, as evidenced by LDH release after a 4-hour exposure to 400 µg/m^3^ O_3_ ([Zavala et al., 2016](https://ujies-my.sharepoint.com/personal/onavarre_uji_es/Documents/Doctorado/2.%20Artículos/1.%20Systematic%20review%20ALI/Texto%20completo%20systematic%20review%20ALI%2031-5-2024%20(Óscar)_JMDS.docx#_ENREF_144)).

In a different study conducted at the University of Mainz, the cytotoxic effects of long-term exposure to ozone on both healthy and chronically inflamed human respiratory mucosa were investigated through various assays, including LDH activity, DNA-staining and the activity of cytosolic esterase ([Gosepath et al., 2000](https://ujies-my.sharepoint.com/personal/onavarre_uji_es/Documents/Doctorado/2.%20Artículos/1.%20Systematic%20review%20ALI/Texto%20completo%20systematic%20review%20ALI%2031-5-2024%20(Óscar)_JMDS.docx#_ENREF_41)). A statistically significant increase in cytotoxicity was observed during the last two weeks of ozone exposure at higher concentrations (i.e., 1000 µg/m^3^). This effect was particularly enhanced in nasal epithelial cells from chronically inflamed mucosa.

- - 1. *Volatile Organic Compounds (VOCs)*

A significant decrease in cell viability of A549 cells (p< 0.05, XTT assay) was reported after exposure to 75 µg/m^3^ formaldehyde (HCHO) for 30 minutes ([Persoz et al., 2010](#_ENREF_107)). However, no significant cytotoxic effects were found in further studies using very similar exposure conditions after a 30-minute exposure to 50 µg/m^3^ HCHO for A549 or BEAS-2B cells ([Persoz et al., 2012](#_ENREF_108)). On the other hand, the exposure of A549 cells to approximately 120 and 610 µg/m^3^ HCHO for 72 hours did not induce statistically significant differences in cell viability, which was evaluated from the reduction of resazurin ([Gostner et al., 2016](#_ENREF_42)).

Health impact assessment of exposure to formaldehyde in terms of cell viability and cytotoxicity showed inconsistent results, since cell viability increased significantly in one study ([Persoz et al., 2010](#_ENREF_107)), but no significant effect was found in three studies ([Gostner et al., 2016](#_ENREF_42); [Kastner et al., 2013](#_ENREF_61)). In fact, cell viability decreased significantly after exposure to 75 µg/m^3^ HCHO for 30 minutes, but it did not change after exposure to 610 µg/m^3^ HCHO for 72 hours.

Human alveolar epithelial cells (A549) were exposed to volatile organic compounds (VOCs) from gasoline using a static exposure system with direct exposure of cell cultures inside a glass exposure chamber ([Sayyed et al., 2022](#_ENREF_123)). After a 1-hour exposure, VOCs significantly reduced cell viability (MTS assay) in a dose-dependent manner, starting from a concentration of 173 ppm. The highest increase in cytotoxicity (LDH assay) was reached immediately after exposure to 337 ppm (64%) and 24 hours after exposure (54%). Moreover, the percentage of apoptotic cells increased dose-dependently, as well as the expression of proteins related with apoptosis assessed by flow cytometry and Western Blot. The same cell line was exposed to ethylbenzene in a different study with very similar results ([Verstraelen et al., 2021](#_ENREF_135)). In this case, cell viability, which was assessed from the MTT assay, decreased dose-dependently to 86% (p = 3.59 x 10^-4^), 77% (p = 9.64 x 10^-9^) and 47% (p = 4.19 x 10^-26^) compared to clean air for the respective increasing exposure concentrations (i.e., 3 x 10^7^, 4 x 10^7^ and 5 x 10^7^ µg/m^3^ EB), and cytotoxicity increased by 9% (p = 7.74 x 10^-4^), 20% (p = 8.34 x 10^-12^) and 36% (p = 1.27 x 10^-24^) in terms of LDH release. On the other hand, a 1-hour exposure to benzene, toluene and xylenes decreased cell viability of A549 cells, but these results were not statistically significant ([Liu et al., 2013](#_ENREF_91)).

- 1. **Aerosols and particulate matter**
     1. *Diesel exhaust particles (DEP)*

In a study conducted at the University of Paris, human nasal epithelial cells (HNE) from patients undergoing turbinectomy were exposed to increasing concentrations of diesel exhaust particle suspensions ranging from 10 to 80 µg/cm^2^ ([Auger et al., 2006](#_ENREF_6)). No cytotoxic effects were reported after measuring the release of lactate dehydrogenase (LDH assay). However, diesel exhaust particles had significant cytotoxic effects on human adenocarcinoma alveolar epithelial cells (A549) both after direct aerosol and liquid suspension exposures. In fact, cytotoxicity increased with the mass of DEP that was deposited in the cells ([Cooney & Hickey, 2011](#_ENREF_29)). Such effects were confirmed by a small but statistically significant decrease in cell viability reported by the MTT assay, which was not observed for human lung adenocarcinoma cells (Calu-3).

In another study, cytotoxic effects of DEP were identified following exposure of A549 cells in a tetra-culture, as a significant time-dependent increase in the release of LDH was observed for both the medium (3.4 µg/cm^2^ DEP) and highest (5 µg/cm^2^ DEP) concentrations ([Fizesan et al., 2018](#_ENREF_36)). However, no decrease in cell viability was reported in a different study following short exposures of A549 tetra-cultures to diesel exhaust particulate matter at concentrations of 0.04, 0.08 and 0.24 µg/cm^2^ ([Klein et al., 2017](#_ENREF_65)).

On the other hand, cell viability of human bronchial epithelial 16HBE14o cells dropped to around 75% of the viability of unexposed cells after exposure to both filtered and whole diesel exhaust at the ALI. Moreover, viability decreased to around 86% of unexposed cells after the same cell line was exposed to particle suspension at concentrations of 2.5 and 12.5 µg/cm^2^ DEP.

Primary bronchial epithelial cells (PBECs) and human monocytes (THP-1) did not experience significant differences in cell viability and cytotoxicity after exposure to 12.7 µg/cm^2^ DEP for 3 minutes ([Ji et al., 2018](#_ENREF_58)).

Human alveolar cells (A549) and bronchial epithelial 16HBE14o cells experienced a decrease in cell viability, but such effect was not observed in Calu-3 cells. Klein *et al.* evaluated the effects of DEP on A549 cells, and found no significant differences in cell viability of A549 cells in a tetra-culture with Ea.hy 926 cells, THP-1 monocytes and human mast cells (HMC-1), while Fizesan *et al.* found a small but statistically significant time-related increased in cytotoxicity using the same cell culture model. On the other hand, Ji *et al.* used PneumaCultex medium, which is special for differentation of PBEC cells at the ALI.

Kunzi *et al.*, reported an increased cytotoxic response in all cell models compared to particle-free air exposure was. The exposures were carried out for 2 hours to the aerosol of GEP at concentrations ranging from 20 to 1000 µg/m^3^, calculated using the Multiple Path Particle Dosimetry Model approximation. Moreover, a significant correlation with particle dose was reported in all cell models, except for cystic fibrosis epithelia.

Only one study from this review examined the effects of atmospherically-aged gasoline exhaust particles (GEP) on human bronchial epithelial cells (Kunzi et al., 2015).

- - 1. *Particulate matter (PM) and secondary aerosol from soot particles (SP)*

In a study focused on human nasal epithelial cells (HNE), no increase in LDH release was reported after 24-hour exposures to PM_2,5_ at doses ranging from 10 to 80 µg/cm^2^, suggesting minimal cytotoxic effects of particle suspensions within that concentration range ([Auger et al., 2006](#_ENREF_6)). In a separate study conducted by Lan *et al.* the effects of particulate matter suspensions were evaluated on submerged and ALI-differentiated cultures of small airway epithelial cells (SAECs). Their findings revealed significant concentration-dependent decreases in cell viability following 24-hour exposures to 45 µg/cm^2^ PM_2,5_ surrogates ([Lan et al., 2021](#_ENREF_78)). Moreover, non-differentiated cells exhibited higher sensitivity to PM_2,5_ surrogates probably due to an easier particle internalization process compared to well differentiated cells. An increase in Caspase-3 activity suggested significant pro-apoptotic effects of particle exposure on cells differentiated at the ALI. In a different study, significant dose-dependent increases in cytotoxicity were reported in human bronchial epithelial cells (HBE) and human bronchial epithelial cells from patients with cystic fibrosis (CF HBE) ([Leni et al., 2020](#_ENREF_83)). These cell cultures were exposed for 4 hours to PM_2,5_ and PM_10_ filter extracts from urban or rural locations in Switzerland, and a positive correlation was found between cytotoxicity and deposited dose in the range between 0.9 and 25.4 μg/cm^2^. In contrast, Vockens *et al.* reported slightly elevated LDH release in normal human bronchial epithelial (NHBE) cells exposed to PMcoarse within ALI cultures. However, the observed results were not significantly different from controls ([Volckens et al., 2009](#_ENREF_136)). The same cell line was studied by Leclercq *et al.* using particle suspensions of PM_4_ as exposure system to carry out 4-hour exposures at doses between 1 and 20 µg/cm^2^. The authors reported statistically significant cytotoxic effects by the reduction of ATP concentration and the increase of glucose-6-phosphate dehydrogenase (G6PD) activity. Interestingly, when comparing NHBE and Diseased Human Bronchial Epithelial cells from Chronic Obstructive Pulmonary Disease (COPD-DHBE), no differences were observed after repeated exposure to PM_4_ ([Leclercq et al., 2016](#_ENREF_80)).

On the other hand, Ghio *et al.* assessed the effects of urban particulate matter (UPM) of different sizes on NHBE and BEAS-2B cells. These cells were exposed to particle suspensions of UPM at concentrations of 2 or 10 µg/µL, and no significant cytotoxic effects were observed based on the LDH assay ([Ghio et al., 2013](#_ENREF_40)).

In a study conducted by He *et al*., Calu-3 cell viability showed no significant differences following the exposure to ultrafine particles (UFP). However, a slight increase in cytotoxicity was reported for the highest dose of around 1.5 µg/cm^2^ after 24-hour incubation ([He et al., 2020](#_ENREF_50)).

Research by Sotty *et al.* revealed that acute and repeated exposure to 5 µg/cm^2^ UFP produced significant reductions in ATP concentrations in COPD-DHBE cell cultures ([Sotty et al., 2019](#_ENREF_127)). When examining A549 cells, Bitterle *et al.* found that exposure to aerosolized ultrafine carbonaceous particles (C-UFP) did not produce any significant change in cell viability ([Bitterle et al., 2006](#_ENREF_20)). However, studies examining the effects of secondary organic aerosols (SOA) from soot particles on A549, Ea.hy926, and NHBE cells identified significantly reduced cell viability and/or increased cytotoxicity as a result of SOA exposure ([Leni et al., 2022](#_ENREF_84); [Offer et al., 2022](#_ENREF_102)).

- 1. **Combined aerosols and gaseous pollutants**
     1. *Diesel and biodiesel exhaust*

Human bronchial epithelial cells (16HBE14o-) experienced a significant decrease in cell viability upon exposure to filtered and whole diesel exhausts of varying compositions and exposure durations ranging from one to six hours. Notably, the impact on cell viability was more pronounced in the whole exhaust exposure group, suggesting that nearly half of the toxicity from the entire mixture could be attributed to gases alone ([Holder et al., 2007](https://ujies-my.sharepoint.com/personal/onavarre_uji_es/Documents/Doctorado/2.%20Artículos/1.%20Systematic%20review%20ALI/Texto%20completo%20systematic%20review%20ALI%209-7-2024%20(Óscar)_JMDS.docx#_ENREF_51)). In a preceding study by Knebel *et al.*, HFBE-21 cell viability decreased substantially after short exposure to diesel exhaust, regardless of the operating conditions or filtration methods, although no statistical analyses were reported. However, they found that cell viability decreased substantially after short exposure to diesel exhaust, and the percentage of living cells increased with further exhaust dilution ([Knebel et al., 2002](https://ujies-my.sharepoint.com/personal/onavarre_uji_es/Documents/Doctorado/2.%20Artículos/1.%20Systematic%20review%20ALI/Texto%20completo%20systematic%20review%20ALI%209-7-2024%20(Óscar)_JMDS.docx#_ENREF_67)). These findings are consistent with the outcomes reported by Holder *et al.* in their study.

Another study focused on human bronchial epithelial cells (HBECs) obtained from patients with lung adenocarcinoma reported decreased cell viability and increased cytotoxicity following exposure to whole diesel, biodiesel and triacetin/biodiesel emissions compared to filtered air. Moreover, this exposure downregulated BCL-2 expression significantly and suppressed CASP3 expression, indicating diesel exhaust-induced cytotoxicity through pro-apoptotic effects ([Vaughan et al., 2019](https://ujies-my.sharepoint.com/personal/onavarre_uji_es/Documents/Doctorado/2.%20Artículos/1.%20Systematic%20review%20ALI/Texto%20completo%20systematic%20review%20ALI%209-7-2024%20(Óscar)_JMDS.docx#_ENREF_134)). Similarly, cytotoxic effects were reported in primary bronchial epithelial cells (PBECs) from tumour-free lung tissue of lung cancer patients, as evidenced by a significant increase in LDH release following high exposure doses to diesel exhaust ([Zarcone et al., 2016](https://ujies-my.sharepoint.com/personal/onavarre_uji_es/Documents/Doctorado/2.%20Artículos/1.%20Systematic%20review%20ALI/Texto%20completo%20systematic%20review%20ALI%209-7-2024%20(Óscar)_JMDS.docx#_ENREF_142)).

- - 1. *Gasoline exhaust*

Yu *et al*. found significant cytotoxic effects on both A549 and BEAS-2B cells following direct aerosol exposure to both filtered and non-filtered gasoline exhaust at the air-liquid interface ([Yu et al., 2017](https://ujies-my.sharepoint.com/personal/onavarre_uji_es/Documents/Doctorado/2.%20Artículos/1.%20Systematic%20review%20ALI/Texto%20completo%20systematic%20review%20ALI%209-7-2024%20(Óscar)_JMDS.docx#_ENREF_141)). Interestingly, their results revealed that A549 cells exposed to filtered gasoline exhaust exhibited significantly higher cell viability compared to the non-filtered exposure group, suggesting a potential reduction in gasoline exhaust toxicity by filtration.

In a study by Rossner *et al.*, a significant and nearly linear increase in cytotoxicity over a 5-day exposure of BEAS-2B and MucilAir™ tissues to complete gasoline-ethanol blend exhaust (E20) was reported ([Rossner et al., 2019](https://ujies-my.sharepoint.com/personal/onavarre_uji_es/Documents/Doctorado/2.%20Artículos/1.%20Systematic%20review%20ALI/Texto%20completo%20systematic%20review%20ALI%209-7-2024%20(Óscar)_JMDS.docx#_ENREF_120)). However, consistent differences in cytotoxicity between control and exposed cells were not observed, and no cytotoxic effects were found either for MucilAir ™ cells. On the other hand, Rossner *et al.* reported statistically significant increases in LDH leakage of MucilAir™ tissues after three and four days of exposure, whereas BEAS-2B cells showed significantly increased cytotoxicity even after a single day of exposure ([Rossner et al., 2021](https://ujies-my.sharepoint.com/personal/onavarre_uji_es/Documents/Doctorado/2.%20Artículos/1.%20Systematic%20review%20ALI/Texto%20completo%20systematic%20review%20ALI%209-7-2024%20(Óscar)_JMDS.docx#_ENREF_119)). Notably, control cells exhibited over 50% cytotoxicity after the third day of exposure, but significant differences were observed between control and exposed groups across all time points.

1. **Cellular inflammation**
   1. **Gaseous pollutants**
      1. *Nitrogen dioxide (NO_2_)*

Kastner *et al.* observed no impact on cytokine secretion in Calu-3 cells upon exposure to nitrogen dioxide alone at concentrations ranging from 200 to 800 µg/m^3^ at the air-liquid interface. However, when exposed to a mixture of NO_2_ and formaldehyde (HCHO) under submerged conditions, a notable increase in IL-8 release was induced ([Kastner et al., 2013](https://ujies-my.sharepoint.com/personal/onavarre_uji_es/Documents/Doctorado/2.%20Artículos/1.%20Systematic%20review%20ALI/Texto%20completo%20systematic%20review%20ALI%209-7-2024%20(Óscar)_JMDS.docx#_ENREF_61)). In another study by Koehler *et al.*, significant concentration-dependent elevation of IL-6 release in human nasal epithelial cells was reported following exposure to NO_2_ concentrations from 200 to 20000 µg/m^3^, whereas IL-8 levels remained unaffected by increasing concentrations ([Koehler et al., 2016](https://ujies-my.sharepoint.com/personal/onavarre_uji_es/Documents/Doctorado/2.%20Artículos/1.%20Systematic%20review%20ALI/Texto%20completo%20systematic%20review%20ALI%209-7-2024%20(Óscar)_JMDS.docx#_ENREF_71)). Interestingly, co-exposures of NO_2_ with Der p1 induced the production of both cytokines at lower NO_2_ concentrations, but not under synthetic air conditions or at the highest NO_2_ concentration.

Mirowsky *et al.* investigated primary human bronchial epithelial cells and revealed that only the highest NO_2_ concentration (10000 µg/m^3^) induced significant increases in IL-8 and PTGS2 mRNA expressions measured by RT-PCR at 4 hours post-exposure (p < 0.01 for IL-8 and p < 0.05 for PTGS2). However, no statistically significant upregulation of IL-6 mRNA expression was observed ([Mirowsky et al., 2016](https://ujies-my.sharepoint.com/personal/onavarre_uji_es/Documents/Doctorado/2.%20Artículos/1.%20Systematic%20review%20ALI/Texto%20completo%20systematic%20review%20ALI%209-7-2024%20(Óscar)_JMDS.docx#_ENREF_99)). Similarly, Verstraelen *et al.* observed that exposure of A549 cells to NO_2_ significantly increased IL-8 secretion but did not induce CCL2 or IL-6 release compared to clean air conditions ([Verstraelen et al., 2021](https://ujies-my.sharepoint.com/personal/onavarre_uji_es/Documents/Doctorado/2.%20Artículos/1.%20Systematic%20review%20ALI/Texto%20completo%20systematic%20review%20ALI%209-7-2024%20(Óscar)_JMDS.docx#_ENREF_135)).

- - 1. *Ozone (O_3_)*

In a study by Gosepath *et al.*, it was observed that the release of IL-8 from both healthy and chronically inflamed human nasal epithelia decreased between the second and fourth week after exposure to ozone. Remarkably, a linear time-dependent reduction of IL-8 levels was observed alongside increased cytotoxicity, indicating a counterbalance between ozone-induced cellular inflammation and decreased cell viability ([Gosepath et al., 2000](https://ujies-my.sharepoint.com/personal/onavarre_uji_es/Documents/Doctorado/2.%20Artículos/1.%20Systematic%20review%20ALI/Texto%20completo%20systematic%20review%20ALI%2011-7-2024%20(Óscar)_JMDS.docx#_ENREF_41)).

McCullough *et al.* found significant inductions of several pro-inflammatory cytokines, including IL-8, IL-6, IL-1α and IL-1β, in primary human bronchial epithelial cells (pHBECs) and BEAS-2B cells following exposures to 1000 µg/m^3^ of ozone for 2 hours ([McCullough et al., 2016](https://ujies-my.sharepoint.com/personal/onavarre_uji_es/Documents/Doctorado/2.%20Artículos/1.%20Systematic%20review%20ALI/Texto%20completo%20systematic%20review%20ALI%2011-7-2024%20(Óscar)_JMDS.docx#_ENREF_96); [McCullough et al., 2014](https://ujies-my.sharepoint.com/personal/onavarre_uji_es/Documents/Doctorado/2.%20Artículos/1.%20Systematic%20review%20ALI/Texto%20completo%20systematic%20review%20ALI%2011-7-2024%20(Óscar)_JMDS.docx#_ENREF_97)). Additionally, Mirowsky *et al.* reported the upregulation of inflammation-related genes such as IL-8 and PTGS2 in PBECs when exposed to ozone concentrations ranging from 1000 to 2000 µg/m^3^ ([Mirowsky et al., 2016](https://ujies-my.sharepoint.com/personal/onavarre_uji_es/Documents/Doctorado/2.%20Artículos/1.%20Systematic%20review%20ALI/Texto%20completo%20systematic%20review%20ALI%2011-7-2024%20(Óscar)_JMDS.docx#_ENREF_99)). Similarly, studies conducted on A549 cells and EpiAirway™ tissues revealed significant increases in IL-8 and IL-6 levels, respectively, following exposure to 1000 µg/m^3^ of ozone for 4 hours ([Zavala et al., 2016](https://ujies-my.sharepoint.com/personal/onavarre_uji_es/Documents/Doctorado/2.%20Artículos/1.%20Systematic%20review%20ALI/Texto%20completo%20systematic%20review%20ALI%2011-7-2024%20(Óscar)_JMDS.docx#_ENREF_144)).

- - 1. *Volatile Organic Compounds (VOCs)*

Kastner *et al.* found that exposing Calu-3 cells to a mixture of NO_2_ and HCHO significantly increased IL-8 release, while single exposure to NO_2_ or formaldehyde at the air-liquid interface did not affect cytokine secretion significantly ([Kastner et al., 2013](https://ujies-my.sharepoint.com/personal/onavarre_uji_es/Documents/Doctorado/2.%20Artículos/1.%20Systematic%20review%20ALI/Texto%20completo%20systematic%20review%20ALI%2011-7-2024%20(Óscar)_JMDS.docx#_ENREF_61)). Conversely, Persoz *et al.* observed an important two-fold increase in IL-8 levels after TNFα pre-sensitization of A549 cells. Moreover, the concentration of IL-8 notably increased following exposure of pre-sensitized cells to formaldehyde at concentrations ranging from 25 to 75 µg/m^3^ ([Persoz et al., 2010](https://ujies-my.sharepoint.com/personal/onavarre_uji_es/Documents/Doctorado/2.%20Artículos/1.%20Systematic%20review%20ALI/Texto%20completo%20systematic%20review%20ALI%2011-7-2024%20(Óscar)_JMDS.docx#_ENREF_107)).

Subsequent studies reported higher increases of IL-8 and MCP-1 for both BEAS-2B and A549 cells after sensitization with macrophage secretion medium compared to non-sensitized cells and TNFα-sensitized cells. Notably, exposure to 50 µg/m^3^ formaldehyde for 30 minutes significantly increased IL-8 release in sensitized A549 cells, while decreasing MCP-1 production in BEAS-2B cells ([Persoz et al., 2012](#_ENREF_108)).

In a different study by Liu *et al.*, A549 cells showed higher sensitivity to cellular inflammation upon acute and chronic exposures to benzene, toluene and xylenes using different exposure systems, although no statistical significance was reported ([Liu et al., 2013](https://ujies-my.sharepoint.com/personal/onavarre_uji_es/Documents/Doctorado/2.%20Artículos/1.%20Systematic%20review%20ALI/Texto%20completo%20systematic%20review%20ALI%2011-7-2024%20(Óscar)_JMDS.docx#_ENREF_91)). On the other hand, acute exposure of the same cell line to gasoline VOCs using a static exposure system did not elicit significant IL-1β or TNFα-related pro-inflammatory responses within 4 or 24 hours after exposure ([Sayyed et al., 2022](https://ujies-my.sharepoint.com/personal/onavarre_uji_es/Documents/Doctorado/2.%20Artículos/1.%20Systematic%20review%20ALI/Texto%20completo%20systematic%20review%20ALI%2011-7-2024%20(Óscar)_JMDS.docx#_ENREF_123)).

However, when using ethylbenzene as exposure agent, significant concentration-dependent increases of all tested pro-inflammatory markers, including CCL2, IL-6 and IL-8, were reported by Vestraelen *et al.* in A549 cells cultured at the ALI ([Verstraelen et al., 2021](https://ujies-my.sharepoint.com/personal/onavarre_uji_es/Documents/Doctorado/2.%20Artículos/1.%20Systematic%20review%20ALI/Texto%20completo%20systematic%20review%20ALI%2011-7-2024%20(Óscar)_JMDS.docx#_ENREF_135)). In fact, statistically significant results were reported for all the concentrations tested, with the highest induction of cytokine expression registered after exposure to 5 x 10^7^ µg/m^3^ ethylbenzene (equivalent to a response observed with 53.6 µg/cm^2^ insert mass particle load). The log2 fold changes (log2 FC) reported for CCL2, IL-6 and IL-8 were of 1.5, 3.3 and 4.5, respectively.

- 1. **Aerosols and particulate matter (PM)**
     1. *Diesel exhaust particles (DEP)*

Auger *et al.* investigated the impact of diesel exhaust particles (DEP) at concentrations of 10-80 µg/cm^2^ on cellular inflammation in human nasal epithelial cells cultured at the air-liquid interface. Their findings revealed a dose-dependent increase in the release of various cytokines such as GM-CSF, IL-6 and IL-8 following 24-hour DEP exposures to ([Auger et al., 2006](https://ujies-my.sharepoint.com/personal/onavarre_uji_es/Documents/Doctorado/2.%20Artículos/1.%20Systematic%20review%20ALI/Texto%20completo%20systematic%20review%20ALI%2011-7-2024%20(Óscar)_JMDS.docx#_ENREF_6)). In a different study, Cooney *et al.* observed dose-dependent elevations in IL-8 release in both Calu-3 cells and notably in A549 cells upon exposure to DEP concentrations ranging from 10 to 100 µg/cm^2^. Moreover, exposure to 100 µg/cm^2^ of DEP induced a fourfold increase in GM-CSF release ([Cooney & Hickey, 2011](https://ujies-my.sharepoint.com/personal/onavarre_uji_es/Documents/Doctorado/2.%20Artículos/1.%20Systematic%20review%20ALI/Texto%20completo%20systematic%20review%20ALI%2011-7-2024%20(Óscar)_JMDS.docx#_ENREF_29)).

Fizesan *et al.* examined the effects of lower doses of DEP on a tetra-culture formed by A549 cells, Ea.hy 926, THP-1 monocytes and HMC-1 mast cells, noting significant concentration-dependent increases in IL-6 and IL-8 release as well as gene expression levels ([Fizesan et al., 2018](https://ujies-my.sharepoint.com/personal/onavarre_uji_es/Documents/Doctorado/2.%20Artículos/1.%20Systematic%20review%20ALI/Texto%20completo%20systematic%20review%20ALI%2011-7-2024%20(Óscar)_JMDS.docx#_ENREF_36)). Klein *et al.* utilized a similar cellular model with even lower exposure concentrations, but did not report statistically significant results for any of the analyzed cytokines (i.e., IL-6, IL-8, TNF-α, GM-CSF and MCP-1) ([Klein et al., 2017](https://ujies-my.sharepoint.com/personal/onavarre_uji_es/Documents/Doctorado/2.%20Artículos/1.%20Systematic%20review%20ALI/Texto%20completo%20systematic%20review%20ALI%2011-7-2024%20(Óscar)_JMDS.docx#_ENREF_65)).

Other studies included in this review consistently showed significant increases in cytokine concentrations (IL-6, IL-8, TNFα) and the upregulation of respective genes responsible for their production following DEP exposure in human bronchial epithelial cells ([Holder et al., 2008](https://ujies-my.sharepoint.com/personal/onavarre_uji_es/Documents/Doctorado/2.%20Artículos/1.%20Systematic%20review%20ALI/Texto%20completo%20systematic%20review%20ALI%2011-7-2024%20(Óscar)_JMDS.docx#_ENREF_52); [Ji et al., 2018](https://ujies-my.sharepoint.com/personal/onavarre_uji_es/Documents/Doctorado/2.%20Artículos/1.%20Systematic%20review%20ALI/Texto%20completo%20systematic%20review%20ALI%2011-7-2024%20(Óscar)_JMDS.docx#_ENREF_58); [Steiner et al., 2013](https://ujies-my.sharepoint.com/personal/onavarre_uji_es/Documents/Doctorado/2.%20Artículos/1.%20Systematic%20review%20ALI/Texto%20completo%20systematic%20review%20ALI%2011-7-2024%20(Óscar)_JMDS.docx#_ENREF_131)). Moreover, two studies assessing the effect of diesel exhaust filtration reported higher increases in cytokine release for cells exposed to whole exhaust ([Holder et al., 2008](https://ujies-my.sharepoint.com/personal/onavarre_uji_es/Documents/Doctorado/2.%20Artículos/1.%20Systematic%20review%20ALI/Texto%20completo%20systematic%20review%20ALI%2011-7-2024%20(Óscar)_JMDS.docx#_ENREF_52); [Steiner et al., 2013](https://ujies-my.sharepoint.com/personal/onavarre_uji_es/Documents/Doctorado/2.%20Artículos/1.%20Systematic%20review%20ALI/Texto%20completo%20systematic%20review%20ALI%2011-7-2024%20(Óscar)_JMDS.docx#_ENREF_131)).

- - 1. *Particulate matter (PM) and secondary aerosol from soot particles (SP)*

For brake wear particulate matter, the release of IL-8 significantly increased in A549 cells when exposed to brake wear debris in a co-culture with human macrophages (MDMs) and dendritic cells (MDDCs) ([Barosova et al., 2018](https://ujies-my.sharepoint.com/personal/onavarre_uji_es/Documents/Doctorado/2.%20Artículos/1.%20Systematic%20review%20ALI/Texto%20completo%20systematic%20review%20ALI%2011-7-2024%20(Óscar)_JMDS.docx#_ENREF_11)). Another study noted a higher release of IL-8 in A549 cell cultures exposed to brake wear PM obtained from a full stop braking behavior, although statistical significance was not reported ([Gasser et al., 2009](https://ujies-my.sharepoint.com/personal/onavarre_uji_es/Documents/Doctorado/2.%20Artículos/1.%20Systematic%20review%20ALI/Texto%20completo%20systematic%20review%20ALI%2011-7-2024%20(Óscar)_JMDS.docx#_ENREF_38)).

Significantly elevated production of key inflammatory mediators, including IL-6 and IL-8, was observed with the highest doses of fine particulate matter (FP) and ultrafine particulate matter (UFP). Specifically, He *et. al.* reported a significant increase in cellular inflammation in Calu-3 cells exposed to UFP derived from airport, non-airport emissions, and turbine engines ([He et al., 2020](https://ujies-my.sharepoint.com/personal/onavarre_uji_es/Documents/Doctorado/2.%20Artículos/1.%20Systematic%20review%20ALI/Texto%20completo%20systematic%20review%20ALI%2011-7-2024%20(Óscar)_JMDS.docx#_ENREF_50)). Similarly, Sotty *et al.* documented comparable effects in diseased COPD-DHBE cells following UFP exposure, while healthy bronchial epithelial cells did not exhibit statistically significant variations ([Sotty et al., 2019](https://ujies-my.sharepoint.com/personal/onavarre_uji_es/Documents/Doctorado/2.%20Artículos/1.%20Systematic%20review%20ALI/Texto%20completo%20systematic%20review%20ALI%2011-7-2024%20(Óscar)_JMDS.docx#_ENREF_127)).

In contrast, A549 cells exposed to aerosols generated from ultrafine carbonaceous particles (C-UFP) in a study by Bitterle *et al.* did not significantly increase mRNA expression of IL-6 and IL-8 ([Bitterle et al., 2006](https://ujies-my.sharepoint.com/personal/onavarre_uji_es/Documents/Doctorado/2.%20Artículos/1.%20Systematic%20review%20ALI/Texto%20completo%20systematic%20review%20ALI%2011-7-2024%20(Óscar)_JMDS.docx#_ENREF_20)).

In a study by Leclercq *et al.*, significantly greater sensitivity to particulate matter (PM_4_) was observed in diseased human bronchial epithelial cells (HBE) compared to NHBE for the increased secretion of TNF-α, IL-1β, IL-8, GM-CSF and TGF-α, although IL-6 levels did not show a significant increase in COPD-DHBE cells ([Leclercq et al., 2016](https://ujies-my.sharepoint.com/personal/onavarre_uji_es/Documents/Doctorado/2.%20Artículos/1.%20Systematic%20review%20ALI/Texto%20completo%20systematic%20review%20ALI%2011-7-2024%20(Óscar)_JMDS.docx#_ENREF_80)). Conversely, Leni *et al.* identified a positive correlation between deposited dose and cytokine release in both NHBE and cystic fibrosis (CF HBE) cells following exposure to PM_2.5_ and PM_10_ ([Leni et al., 2020](https://ujies-my.sharepoint.com/personal/onavarre_uji_es/Documents/Doctorado/2.%20Artículos/1.%20Systematic%20review%20ALI/Texto%20completo%20systematic%20review%20ALI%2011-7-2024%20(Óscar)_JMDS.docx#_ENREF_83)). Moreover, NHBE cells exposed to PMcoarse exhibited significantly increased mRNA levels of IL-8, COX-2, and HOX-1, particularly with direct-air exposure as opposed to liquid exposure ([Volckens et al., 2009](https://ujies-my.sharepoint.com/personal/onavarre_uji_es/Documents/Doctorado/2.%20Artículos/1.%20Systematic%20review%20ALI/Texto%20completo%20systematic%20review%20ALI%2011-7-2024%20(Óscar)_JMDS.docx#_ENREF_136)). Lan *et al.* found significantly overexpressed levels of IL-2, IL-6 and IL-12 after a 24-h exposure of small airway epithelial cells differentiated at the ALI to 22 µg/cm^2^ PM2.5 surrogate suspensions ([Lan et al., 2021](https://ujies-my.sharepoint.com/personal/onavarre_uji_es/Documents/Doctorado/2.%20Artículos/1.%20Systematic%20review%20ALI/Texto%20completo%20systematic%20review%20ALI%2011-7-2024%20(Óscar)_JMDS.docx#_ENREF_78)).

On the other hand, secondary aerosols from soot particles (SPs) were found to induce cytokine release in both human bronchial epithelial cells ([Leni et al., 2022](https://ujies-my.sharepoint.com/personal/onavarre_uji_es/Documents/Doctorado/2.%20Artículos/1.%20Systematic%20review%20ALI/Texto%20completo%20systematic%20review%20ALI%2011-7-2024%20(Óscar)_JMDS.docx#_ENREF_84)) and A549 cells ([Offer et al., 2022](https://ujies-my.sharepoint.com/personal/onavarre_uji_es/Documents/Doctorado/2.%20Artículos/1.%20Systematic%20review%20ALI/Texto%20completo%20systematic%20review%20ALI%2011-7-2024%20(Óscar)_JMDS.docx#_ENREF_102)). Notably, pro-inflammatory effects were more pronounced in HBE cells following exposure to the 30 nm SPs, and statistically significant differences were reported between the different coatings.

- 1. **Combined aerosols and gaseous pollutants**

The pro-inflammatory effects of exposing 16HBE14o- cells to both winter and summer ambient air were investigated in a study conducted by Bisig *et al.* in Fribourg ([Bisig et al., 2018](https://ujies-my.sharepoint.com/personal/onavarre_uji_es/Documents/Doctorado/2.%20Artículos/1.%20Systematic%20review%20ALI/Texto%20completo%20systematic%20review%20ALI%2011-7-2024%20(Óscar)_JMDS.docx#_ENREF_18)). The study revealed that exposure to winter air significantly increased protein levels and gene expression of IL-8, IL-1β, and TNF-α, while no statistically significant results were reported for summer air exposure.

In the context of diesel and biodiesel exposures, no significant alterations in IL-8 levels were observed in human bronchial epithelial (HBE) cells following exposure to diesel exhaust. Conversely, IL-8 release showed a significant increase following exposure to B90T10 and B100 biodiesel-triacetin mixtures ([Vaughan et al., 2019](https://ujies-my.sharepoint.com/personal/onavarre_uji_es/Documents/Doctorado/2.%20Artículos/1.%20Systematic%20review%20ALI/Texto%20completo%20systematic%20review%20ALI%2011-7-2024%20(Óscar)_JMDS.docx#_ENREF_134)). Other studies focused on 16HBE14o- cells ([Holder et al., 2007](https://ujies-my.sharepoint.com/personal/onavarre_uji_es/Documents/Doctorado/2.%20Artículos/1.%20Systematic%20review%20ALI/Texto%20completo%20systematic%20review%20ALI%2011-7-2024%20(Óscar)_JMDS.docx#_ENREF_51)) and primary bronchial epithelial cells ([Zarcone et al., 2016](https://ujies-my.sharepoint.com/personal/onavarre_uji_es/Documents/Doctorado/2.%20Artículos/1.%20Systematic%20review%20ALI/Texto%20completo%20systematic%20review%20ALI%2011-7-2024%20(Óscar)_JMDS.docx#_ENREF_142)) reported notable increases in IL-8 levels following exposure to both whole and filtered diesel exhaust. These increases were more pronounced in the groups exposed to whole diesel exhaust (DE) compared to the filtered exposure groups, underscoring the substantial pro-inflammatory effects of diesel exhaust gases alone. In another study by Upadhyay *et al.*, there was a significant decrease in IL-8 release following repeated exposures to diesel exhaust particles (DEP), whereas high concentrations of NO_2_ and SO_2_ increased IL-8 levels 24 hours after exposure ([Upadhyay et al., 2022](https://ujies-my.sharepoint.com/personal/onavarre_uji_es/Documents/Doctorado/2.%20Artículos/1.%20Systematic%20review%20ALI/Texto%20completo%20systematic%20review%20ALI%2011-7-2024%20(Óscar)_JMDS.docx#_ENREF_133)).

In a different study, BEAS-2B cells and MucilAir™ tissues were exposed to a complete gasoline-ethanol blend exhaust, and distinct pro-inflammatory responses were observed based on the exhaust composition and cell type. Specifically, E5 gasoline exhaust exhibited a significantly greater impact on MucilAir™ tissues in comparison to BEAS-2B cells. In contrast, E20 exhaust elevated cytokine levels in BEAS-2B cells while eliciting a minimal response in MucilAir™ tissues ([Rossner et al., 2021](https://ujies-my.sharepoint.com/personal/onavarre_uji_es/Documents/Doctorado/2.%20Artículos/1.%20Systematic%20review%20ALI/Texto%20completo%20systematic%20review%20ALI%2011-7-2024%20(Óscar)_JMDS.docx#_ENREF_119)).

1. **Genotoxicity**
   1. **Gaseous pollutants**

The genotoxic effects of nitrogen dioxide on human nasal epithelial cells (HNE) were investigated in a series of studies conducted by Koehler *et al.* ([Koehler et al., 2010](#_ENREF_69), [2011](#_ENREF_70); [Koehler et al., 2013](#_ENREF_72)). Exposure to NO_2_ at concentrations ranging from 20 to 20000 µg/m^3^ resulted in a concentration-dependent increase in the Olive Tail Moment (OTM) of DNA, with a significant dose-dependent increase observed at 20 and 200 µg/m^3^ NO_2_. Moreover, a significant change in micronucleus induction was reported when comparing 3-hour exposure to 200 µg/m^3^ NO_2_ with 0.5-hour exposure to the same concentration. In contrast, Mirowsky *et al.* reported the downregulation of DNA damage-related genes (e.g., ATM) in primary bronchial epithelial cells (pHBECs) following NO_2_ exposure at concentrations ranging from 2000 to 10000 µg/m^3^. However, these genes were upregulated after exposure to 500-2000 µg/m^3^ ozone ([Mirowsky et al., 2016](#_ENREF_99)).

In a separate study conducted by Gostner *et al.*, the genotoxicity of formaldehyde was evaluated in A549 cells. Upon exposure to concentrations of 120 µg/m^3^ and 610 µg/m^3^ of HCHO, 312 and 351 differentially expressed transcripts were detected, respectively. Metabolic processes were more significantly affected at lower concentrations, while higher concentrations had a greater impact on cell adhesion, differentiation, and proliferation processes ([Gostner et al., 2016](#_ENREF_42)).

Mascelloni *et al.* observed statistically significant differences in the percentage of DNA in tail (TD%) between A549 cells exposed to 320 and 960 µg/m^3^ benzene and their respective controls. Moreover, there was a concentration-dependent increase in DNA damage following exposure to benzene within the tested concentration range (R^2^= 0.718) ([Mascelloni et al., 2015](#_ENREF_94)). These results are in accordance with those reported by Liu *et al.*, who noted genotoxic effects in A549 cells after 24-hour benzene exposure, as evidenced by a higher percentage of cells with DNA damage correlating with increased benzene concentration, although statistical significance was not reported in this study ([Liu et al., 2013](#_ENREF_91)). Sayyed *et al.* observed a slight induction of DNA damage 4 hours after the exposure of A549 cells to 114 and 229 ppm gasoline VOCs. However, statistically significant genotoxic effects were reported following exposure to 458 ppm of gasoline VOCs, both immediately and 24 hours after exposure ([Sayyed et al., 2022](#_ENREF_123)).

- 1. **Aerosols and particulate matter (PM)**

Iwanaga *et al.* reported a significantly higher increase in the gene expression of MUC5AC in healthy primary airway epithelial cells (AECs) exposed to a suspension of UPM compared to asthmatic cells, whereas IL-8 gene expression followed the opposite trend ([Iwanaga et al., 2013](#_ENREF_55)). However, Leclercq *et al.* observed that epigenetic alterations induced by repeated exposure to PM4 suspension were more pronounced in diseased human bronchial epithelial cells (COPD-DHBE) than in healthy cells (NHBE), based on the significant shortening of telomeres and an increase in telomerase activity, especially in diseased cells ([Leclercq et al., 2016](#_ENREF_80)). These results are consistent with the higher number of differentially expressed genes reported by Sotty *et al.* in asthma- and especially COPD-DHBE cells following acute and repeated exposures to fine (FP) and quasi ultra-fine particles (UFP) ([Sotty et al., 2019](#_ENREF_127)).

In a separate study, Despréaux *et al.* investigated the genotoxic effects of particulate matter exposure in both healthy and asthmatic reconstituted human bronchial epithelia ([Despréaux et al., 2023](#_ENREF_32)) Particles were sampled in two distinct areas using a cascade impactor with five stages plus a back-up, and their chemical composition was analyzed. Significant changes in gene expression were observed in healthy epithelia following exposure to both types of PM at a concentration of 90 µg/cm^2^, with a common upregulation of CYP1B1. Graph analysis identified differentially expressed genes for all exposure conditions, including those associated with inflammatory and epigenetic mechanisms such as CXCL10 and HDAC6. Moreover, a more pronounced modulation of gene expression was observed after exposing asthmatic epithelia to PM compared to healthy epithelia. Classical analysis revealed significant dose-dependent alterations of genes involved in inflammation (CXCL2, CXCL10 and TLR4), epigenetic mechanisms (e.g., HDAC1) and metabolism (e.g., CYP1B1).

Offer *et al.* found a significant increase in DNA breaks following exposure of EA.hy926 cells to secondary organic aerosol from soot particles, and this effect was more pronounced in A549 cells following exposure to undiluted aerosol ([Offer et al., 2022](#_ENREF_102)).

- 1. **Combined aerosols and gaseous pollutants**

Cervena *et al.* observed increased H2AX phosphorylation in MucilAir™ cells after five days, indicating significant DNA damage, while histone levels in BEAS-2B cells notably decreased after the same period. In contrast, Rossner *et al.* noted a decrease in H2AX phosphorylation with prolonged exposure in both cell types, despite detecting increased double-strand DNA breaks in BEAS-2B cells. Moreover, CYP1A1 expression increased in MucilAir™ cultures over both exposure periods, and one-day treatment led to reduced HSPA5 expression ([Rossner et al., 2019](#_ENREF_120)).

1. **Oxidative stress**
   1. **Gaseous pollutants**

Human bronchial epithelial cells consistently showed significant increases in oxidative stress-related biomarkers across various studies upon exposure to ozone at concentrations ranging from 400 to 2000 µg/m^3^. For instance, McCullough *et al.* reported significant fold increases in the activation of protein kinases related to oxidative stress, including MEK1/2, ERK1/2 and p38, following the exposure of primary human bronchial epithelial cells (pHBECs) to 1000 µg/m^3^ O_3_ for 0.5-2 hours ([McCullough et al., 2014](#_ENREF_97)). Moreover, they observed a highly induced expression of HMOX-1 in pHBECs exposed to ozone under the same conditions ([McCullough et al., 2016](#_ENREF_96)). This oxidative stress-induced upregulation of HMOX-1 was also reported by Mirowsky *et al.* in a similar study using the same cell line but with higher exposure doses ([Mirowsky et al., 2016](#_ENREF_99)). In another study, Ritter *et al.* observed a significant increase in oxidized glutathione levels as cell viability decreased following exposure to O_3_, in both human lung fibroblasts (Lk004) and human bronchial epithelial cells (HFBE-21) ([Ritter et al., 2001](#_ENREF_117)).

Exposure of primary human bronchial epithelial cells (pHBECs) to 10000 µg/m³ NO_2_ resulted in the upregulation of several oxidative stress-related genes, such as HMOX1 and TXNRD1, with significantly greater effects compared to O_3_ exposure ([Mirowsky et al., 2016](#_ENREF_99)). In a separate study by Verstraelen *et al.*, a statistically significant increase in HMOX1 expression was observed following the exposure of A549 cells to NO_2_ compared to clean air exposure. Exposure to ethylbenzene increased SOD2 expression in the same cellular model ([Verstraelen et al., 2021](#_ENREF_135)). As reported by Ritter *et al.*, nitrogen dioxide induced a dose-dependent decrease in intracellular glutathione levels in HFBE-21 cells, which suggests a considerable oxidative effect of this gas ([Ritter et al., 2001](#_ENREF_117)).

- 1. **Aerosols and particulate matter (PM)**
     1. *Diesel exhaust particles (DEP)*

Auger *et al.* noted a dose-dependent increase in intracellular ROS of human nasal epithelial cells exposed to DEP and PM2.5, although these results did not reach statistical significance ([Auger et al., 2006](#_ENREF_6)). In contrast, Cooney & Hickey reported statistically significant increases in ROS following exposure to 50 µg/cm^2^ and 100 µg/cm^2^ DEP ([Cooney & Hickey, 2011](#_ENREF_29)). Fizesan *et al.* observed significant induction of genes encoding GST and HMOX-2 in the apical compartment of A549 cells in a tetra-culture 24 hours after DEP exposure, together with a dose-dependent increase in HMOX-1 release ([Fizesan et al., 2018](#_ENREF_36)). In the same way, Klein *et al.* reported significant upregulation of HSP70 expression using a similar cellular model ([Klein et al., 2017](#_ENREF_65)).

In a different study, exposure of primary bronchial epithelial cells (PBECs) to DEP significantly upregulated the expression of NFKB, HMOX1, and GPx. Interestingly, this induction of oxidative stress was mitigated when PBECs were co-cultured with THP-1 derived human macrophages ([Ji et al., 2018](#_ENREF_58)). In the case of 16HBE14o- cells, Steiner *et al.* observed statistically significant increases in total reduced glutathione (GSH) levels and the expression of HMOX1 and SOD1, after exposure to both filtered and unfiltered exhaust. Diesel exhaust filtration significantly affected GSH oxidation and had weak but significant effects for SOD1 expression ([Steiner et al., 2013](#_ENREF_131)).

- - 1. *Particulate matter (PM) and secondary organic aerosol from soot particles (SOA-SP)*

Barosova *et al.* did not observe statistically significant alterations in reduced GSH levels in A549 cells, human monocyte derived macrophages and dendritic cells following exposure to brake wear particulate matter ([Barosova et al., 2018](#_ENREF_11)). Conversely, Gasser *et al.* noted an increased production of reactive oxygen species (ROS) in A549 cells after exposure to brake wear particulate matter compared to controls ([Gasser et al., 2009](#_ENREF_38)). In a separate study, Kaur *et al.* reported elevated CYP1A1 gene expression in A549 cells and human macrophages following exposure to jet-fuel particulate matter ([Kaur et al., 2022](#_ENREF_62)).

On the other hand, A549 cells exposed to carbonaceous ultrafine particles (C-UFP) at the air-liquid interface (ALI) exhibited significantly elevated HMOX1 expression levels, as reported by Bitterle *et al.* ([Bitterle et al., 2006](#_ENREF_20)). Offer *et al.* reported concentration-dependent increases in malondialdehyde (MDA) release in A549 and Ea.hy926 cells after exposure to secondary organic aerosols SOA_NAP_-SP (R^2^= 0.98, p= 0.0002) and SOA_βPIN_-SP (R^2^= 0.8, p = 0.15) ([Offer et al., 2022](#_ENREF_102)).

Leclercq *et al.* observed significantly higher sensitivity of diseased human bronchial epithelial cells (COPD-DHBE) to oxidative stress induction by particle suspension exposure to PM4 compared to normal human bronchial epithelial cells (NHBE). Differences were reflected in both cell types by increased protein-CO concentration, total antioxidant status (TAS), and glutathione levels compared to controls, while MDA, 8-OHdG, and SOD activity showed no significant differences between cell lines ([Leclercq et al., 2016](#_ENREF_80)). Regarding PM2.5 and PM10, Leni *et al.* observed significantly increased expression of SOD1, SOD2, HMOX1, NFL2L, PRDX, and NQO1 in both NHBE and diseased HBE cells from cystic fibrosis (CF HBE) ([Leni et al., 2020](#_ENREF_83)). However, Ghio *et al.* found that differentiation of NHBE and BEAS-2B cells at the ALI was associated with a diminished response to oxidative stress induced by urban particulate matter (UPM), reflected by a significant decrease in the gene expression of HMOX1 ([Ghio et al., 2013](#_ENREF_40)).

- 1. **Combined aerosols and gaseous pollutants**

Bisig *et al.* documented a significant increase in HMOX1 levels in a co-culture of the human bronchial epithelial cell line 16HBE14o-, monocyte-derived macrophages (MDM) and monocyte-derived dendritic cells (MDDC) after exposure to winter air from Fribourg. However, no significant changes in HMOX1 and NQO1 were observed after exposure to summer air ([Bisig et al., 2018](#_ENREF_18)).

Upadhyay *et al.* reported time-dependent increases in the expression of GSTA1, HMOX1 and SOD3 in primary bronchial epithelial cells (PBECs) following 12.5 μg/cm^2^ DEP exposure. Significant inductions of TNF, GSTA1, and SOD3 expression were also observed 24 hours after exposure to DEP combined with NO_2_ and SO_2_ ([Upadhyay et al., 2022](#_ENREF_133)). In a similar study, Zarcone *et al.* reported dose-dependent increases in HMOX1 and NQO1 expression in PBECs exposed to diesel exhaust ([Zarcone et al., 2016](#_ENREF_142)).

Concerning diesel and biodiesel exhausts, Vaughan *et al.* noted disparities in the gene expression changes induced by oxidative stress in human bronchial epithelial cells (HBECs). Specifically, HO-1 expression was significantly down-regulated after exposure to D100 or B96, while being upregulated with B20, B50, and B90 exposures. Moreover, expression of CYP1A1 was suppressed after D100 and B96 exposures but increased significantly with B90 compared to D100 ([Vaughan et al., 2019](#_ENREF_134)). However, exposure to gasoline exhaust increased intracellular ROS production in both A549 and BEAS-2B cells, as described by Yu *et al.* ([Yu et al., 2017](#_ENREF_141)).
