# Supplementary material for: Toxicological Effects of Air Pollutants on Human Airway Cell Models Using Air–liquid Interface Systems: A Systematic Review
Source: Curr Environ Health Rep. 2025 Jul 28;12(1):26. doi: 10.1007/s40572-025-00491-w (PMC12304069; doi:10.1007/s40572-025-00491-w)
Supplement: Supplementary file 5 — Supplementary file5 (DOCX 205 KB) [file 40572_2025_491_MOESM5_ESM.docx]

**Risk of bias assessment using the OHAT Handbook**

**Table S1. Study quality/risk of bias criteria for *in vitro* epidemiology studies evaluating the effect of key ambient air gaseous pollutants on human airway ALI cultures.**

|  | **Key criteria** | | | **Other quality criteria** | | | |
| --- | --- | --- | --- | --- | --- | --- | --- |
|  | **Can we be confident in the exposure characterization?** | **Were experimental conditions identical across study groups?** | **Can we be confident in the outcome assessment?** | **Did the study employ appropriate statistical approaches?** | **Were all measured outcomes reported?** | **Was the administered dose or exposure level adequately randomized?** | **Did the study assess the temporality of exposure and outcome?** |
| **Definitely low RoB (++)** | There is direct evidence that the exposure was characterized using methods that directly measure gas exposure levels, and that exposure was consistently administered with the same method and timeframe across treatment groups. | There is direct evidence that cell culture conditions and other non-treatment-related experimental conditions (e.g., incubator and plate conditions, medium change schedule and washes, solvents used) were identical across study groups, including controls. | There is direct evidence that the outcomes were assessed using well-established methods (the gold standard), and at the same length of time after initial exposure to gases in all study groups. | There is direct evidence that the study employed appropriate statistical methods. | There is direct evidence that all measured outcomes that are relevant for the study (outlined in the protocol, methods, abstract, and/or introduction)  have been reported. | There is direct evidence that the exposure dose was not administered selectively based on the different cell types or tissues, and a homogeneous cell suspension was obtained. Therefore, each cell had an equal chance to be assigned to any study group, including controls. | There is direct evidence that the exposure precedes the outcome, and a  reasonable time  between exposure and outcome measurement was  examined. |
| **Probably low RoB (+)** | There is indirect evidence that the exposure was characterized using methods that directly measure gas exposure levels, and that exposure was consistently administered with the same method and timeframe across treatment groups. | There is indirect evidence that non-treatment-related experimental conditions were identical across study groups, including controls, or it can be assumed if authors did not report any differences. | There is indirect evidence that the outcomes were assessed using valid and reliable methods (but not the gold standard), and at the same length of time after initial exposure in all study groups, or it is considered that the outcome assessment methods used would not appreciably bias results. | There is indirect evidence that the study employed appropriate statistical methods. | There is indirect evidence that all measured outcomes that are relevant for the study have been reported, or unplanned analyses are clearly identified, and selective reporting of the outcomes (i.e., only reporting statistically significant results) would not appreciably bias results. | There is indirect evidence that the exposure dose was not administered selectively based on the different cell types or tissues, and a homogeneous cell suspension was obtained. Therefore, each cell had an equal chance to be assigned to any study group, including controls. | There is direct evidence that the exposure precedes the outcome, but an  unreasonable time  between exposure and outcome measurement was  examined. |
| **Probably high RoB (−)** | There is indirect evidence that gas exposure was characterized using poorly validated methods, or insufficient information is provided about the validity of the assessment method, but no evidence for concern (NR). | There is indirect evidence that non-treatment-related experimental conditions were not comparable between study groups, or the authors did not report experimental conditions in enough detail (NR). | There is indirect evidence that the outcome assessment methods are unreliable, or the length of time after initial exposure to gases differed by study group. | There is indirect evidence that the study did not use appropriate statistical methods. | There is indirect evidence that all measured outcomes that are relevant for the study have not been reported, and/or indirect evidence that unplanned analyses may appreciably bias results, or there is not enough information provided about selective outcome reporting (NR). | There is indirect evidence that exposure dose was administered selectively based on the different cell types, and/or a homogeneous cell suspension was not obtained. Thus, cell number, cell type or administered dose could be different between study groups. | There is indirect evidence that the exposure precedes the outcome, but time between exposure and outcome measurement was unclear and not examined. |
| **Definitely high RoB (−−)** | There is direct evidence that gas exposure was characterized using poorly validated methods. | There is direct evidence that control and test cell culture conditions were not comparable. | There is direct evidence that the outcome assessment methods are unreliable, or the length of time after initial exposure to gases differed by study group. | There is direct evidence that the study did not use appropriate statistical methods, or did not use any statistical  methods to  compare control and treated groups. | There is direct evidence that all measured outcomes that are relevant for the study have not been reported, or outcomes are reported using analysis methods that were not pre-specified, or unplanned analyses would appreciably bias results. | There is direct evidence that exposure dose was administered selectively based on the different cell types, and/or a homogeneous cell suspension was not obtained. Thus, cell number, cell type or administered dose could be different between study groups | There is no evidence that the exposure precedes the outcome,  or there is evidence that the outcome can precede the exposure. |

***Koehler et al., 2010 (Tier 1)***

| **RoB criteria** | **RoB assessment** | **Evidence** |
| --- | --- | --- |
| **Can we be confident in the exposure characterization?** | **++** | *«The dilution mixture was* ***analyzed online every 30 s*** *with respect to its NO_2_ concentration to ensure a stable gas dilution throughout the entire experiment».* |
| **Were experimental conditions identical across study groups?** | **+** | **No evidence of any difference between exposure and control groups regarding non-treatment related experimental conditions.** |
| **Can we be confident in the outcome assessment?** | **++** | *«In all experiments, the trypan blue assay showed a viability of nearly 100%...».* **Gold standard assay for cell viability.** *«The Comet assay did show DNA fragmentations…».* **Gold standard assay for genotoxicity.** **No evidence of different methods for outcome assessment between study groups.** |
| **Did the study employ appropriate statistical approaches?** | **++** | *«The* ***non-parametric Friedman test*** *was used for related samples. In the case of significance with the Friedman test, a more detailed analysis was done using the* ***non-parametric Wilcoxon test*** *for two related samples».* |
| **Were all measured outcomes reported?** | **++** | *«In all experiments, the* ***trypan blue assay*** *showed a viability of nearly 100%...». «In all 10 samples, a concentration-dependent increase in the* ***Olive Tail Moment*** *as an indicator of genotoxic effects could be seen». «In the first analysis of the* ***micronucleus assay****, the double-nucleated cells were counted per 1000 cells…».* |
| **Was the administered dose or exposure level adequately randomized?** | **+** | *«Inside the exposure chamber, the cells on the membranes were humidified and supplied with nutrients by the culture medium. No further humidification or other modifications of the atmosphere were performed».* **No evidence of selective administration of exposure dose between groups.** |
| **Did the study assess the temporality of exposure and outcome?** | **+** | *«Micronucleus assay* ***results*** *of all 10 experiments* ***after 30-min exposure*** *to the different gas mixtures and NO_2_ concentrations».*  *«After staining, these micronuclei can be detected and counted microscopically as an indicator of DNA damage».* **Direct evidence that the exposure precedes the outcome, and indirect evidence of reasonable time passed between exposure and outcome assessment.** |

***Koehler et al., 2011 (Tier 1)***

| **RoB criteria** | **RoB assessment** | **Evidence** |
| --- | --- | --- |
| **Can we be confident in the exposure characterization?** | **++** | *«Afterwards, the concentration of the dilution was analysed by an* ***NO_2_ analysator online*** *during the total exposure duration»* |
| **Were experimental conditions identical across study groups?** | **+** | *«…the cultures reached an air–liquid interface condition, which was maintained from day 7 to 14 to stabilise the culture conditions. Therefore all the cells were in passage 1». «Just before exposure the membranes were checked microscopically. All culture membranes had the same state of confluence».* **No evidence of any difference in experimental conditions between exposure and control cell cultures.** |
| **Can we be confident in the outcome assessment?** | **++** | *«The trypan blue assay showed a viability of 95–100% in all experiments»*. **Gold standard assay for cell viability.** *«In all 10 samples, an exposure duration-dependent increase in the OTM as an indicator of genotoxicity could be seen after exposure to NO_2_».* *«****Comet assay*** *results of all 10 experiments after 0.5 h, 1 h, 2 h and 3 h exposure…»* **Gold standard assay for genotoxicity. No evidence of different length of time for outcome assessment between groups after the exposure.** |
| **Did the study employ appropriate statistical approaches?** | **++** | *«We used the* ***non-parametric Friedman test*** *for related samples.*  *In the case of significance with the Friedman test, a more detailed analysis was done using the* ***non-parametric Wilcoxon test*** *for two related samples».* |
| **Were all measured outcomes reported?** | **++** | *«The* ***trypan blue assay*** *showed a viability of 95–100% in all experiments»*. «*All data including percentage of DNA in the tail (****DT****), the tail length (****TL****) and the olive tail moment (****OTM****) are presented…». «The data show a significant change in* ***micronucleus*** ***induction*** *by a 3-h exposure…»* |
| **Was the administered dose or exposure level adequately randomized?** | **+** | **No evidence of selective administration of exposure doses between groups of cell cultures.** |
| **Did the study assess the temporality of exposure and outcome?** | **+** | *«Micronucleus assay results of all 10 experiments after 0.5 h, 1 h, 2 h and 3 h exposure». «After staining, these micronuclei were detected and counted microscopically as an indicator of DNA damage».* **Direct evidence that the exposure precedes the outcome, and indirect evidence of reasonable time passed between exposure and outcome assessment** |

***Koehler et al., 2013 (Tier 1)***

| **RoB criteria** | **RoB assessment** | **Evidence** |
| --- | --- | --- |
| **Can we be confident in the exposure characterization?** | **++** | *«After the dilution process, the concentration of the dilution was analyzed by an* ***NO_2_ analysator online*** *during the total exposure duration…»* |
| **Were experimental conditions identical across study groups?** | **+** | **No evidence of any difference in experimental conditions between exposure and control cell cultures.** |
| **Can we be confident in the outcome assessment?** | **++** | *«To detect apoptosis, caspase-3 activity membranes were analyzed using the human active* ***caspase-3 ELISA*** *Set».* **Reliable assay for cytotoxicity assessment.** *«The alkali version of the single cell microgel electrophoresis assay for determining SSBs, alkali labile sites and excision repair sites was used…»* **Gold standard assay for genotoxicity.** **No evidence of different length of time for outcome assessment between groups after the exposure.** |
| **Did the study employ appropriate statistical approaches?** | **++** | *«We used the* ***non-parametric Friedman test*** *for related samples at different exposure durations. Thereby, exposures of either NO_2_ or synthetic air were compared. To compare equal exposure durations of NO_2_ or synthetic air, the* ***non-parametric Wilcoxon***  ***test*** *for two related samples was used».* |
| **Were all measured outcomes reported?** | **++** | *«Apoptosis was analyzed by* ***caspase-3 activity****…The data are presented in Supplementary Table S1». «All data including* ***DT****,* ***TL*** *and* ***OTM*** *are presented in Supplementary Table S2». There were no significant changes in the fraction of the* ***binucleated*** ***cells*** *at any exposure duration (p>0.05) (Supplementary Table S3) ».* |
| **Was the administered dose or exposure level adequately randomized?** | **+** | *«Cells were exposed to either nitrogen dioxide 0.01 ppm or synthetic air at exposure durations of 0.5, 1, 2 or 3 h».* **No evidence of any difference in these exposure doses between groups of cell cultures.** |
| **Did the study assess the temporality of exposure and outcome?** | **++** | *«The boxplots represent caspase-3 activity in units/ml 24 h* ***after exposure****…»* **Direct evidence that the exposure precedes the outcome, and a reasonable time passed between exposure and outcome assessment.** |

***Koehler et al., 2016 (Tier 1)***

| **RoB criteria** | **RoB assessment** | **Evidence** |
| --- | --- | --- |
| **Can we be confident in the exposure characterization?** | **++** | *«After the dilution process, the concentration of the dilution was* ***analyzed by an inline NO_2_ analyzer*** *during the entire exposure duration (Ansyco GmbH, Karlsruhe, Germany), verifying that the gas dilution was stable during all experiments».* |
| **Were experimental conditions identical across study groups?** | **+** | *«At this point, the cultures reached an air-liquid interface condition, which was maintained from day 7 to 14 to stabilize the culture conditions. Therefore, all the cells were in passage 1. Subsequently, the membranes with the cells were used for NO2 and control exposure». «Just before exposure, the Transwell membranes with the nasal epithelial cells were checked microscopically».* **No evidence of any difference in experimental conditions between exposure and control cell cultures.** |
| **Can we be confident in the outcome assessment?** | **++** | *«To determine the IL-6 and IL-8 concentrations, the cell medium of the samples was analyzed by human IL-6 and IL-8 ELISA kits». «The effects of gas exposure and Der p 1 exposure on mRNA expression of IL-6 and IL-8 were analyzed using RT-PCR».* **Gold standard assays for the quantification of cytokines and mRNA expression. No evidence of different length of time for assessment between exposure groups.** |
| **Did the study employ appropriate statistical approaches?** | **++** | *«We used the* ***nonparametric Friedman test*** *for related samples at different gas exposure concentrations. To compare equal exposure concentrations with or without Der p 1, the* ***nonparametric Wilcoxon test*** *for 2 related samples was used».* |
| **Were all measured outcomes reported?** | **++** | *«The* ***ELISA*** *data are presented in table 1». «All data, including the* ***relative gene expression*** *of IL-6 and IL-8 mRNAs, are presented in table 2»* |
| **Was the administered dose or exposure level adequately randomized?** | **+** | *«The membranes were exposed to either 0.1 ppm NO_2_, 1 ppm*  *NO_2_, 10 ppm NO_2_ (Linde Gas Germany), or synthetic air free of*  *hydrocarbons (Linde Gas Germany) for 1 h. For each experiment,*  *1 membrane was used as a negative control and was not exposed».* **No evidence of bias in the allocation of exposure groups.** |
| **Did the study assess the temporality of exposure and outcome?** | **+** | *«The boxplots represent IL-6 levels after exposure to synthetic air or NO_2_ at increasing concentrations alone and in coexposure with Der p 1».* **Direct evidence that the exposure precedes the outcome, and indirect evidence of reasonable time passed between exposure and outcome assessment** |

***Persoz et al., 2010 (Tier 1)***

| **RoB criteria** | **RoB assessment** | **Evidence** |
| --- | --- | --- |
| **Can we be confident in the exposure characterization?** | **++** | *«Measurement of FA air level in the atmosphere chamber was carried out using a technique adapted from Dassonville et al. (2009) using a passive sorbent sampler (…) placed in the atmosphere chamber for 3 h».* **Direct measurement of gaseous formaldehyde levels.** |
| **Were experimental conditions identical across study groups?** | **+** | *«Passages 4–20 were used for all experiments». «For all experiments and after exposure, inserts containing cells were replaced in a 12-well culture plate with fresh culture medium…»* **No evidence of any difference in experimental conditions between exposure and control groups.** |
| **Can we be confident in the outcome assessment?** | **++** | *«Colorimetric XTT (…) assay was performed to assess the mitochondrial metabolic activity of the cells and thus cell viability».* **Reliable assay for cytotoxicity assessment.** *«Cellular inflammation response was assessed by quantification of 3 cytokines (…) using ELISA assays…».* **Gold standard assay for cellular inflammation.** *«A549 cells were exposed to synthetic clean air for 30 min or 60 min and were cultured for an additional 24 h period. These two times of exposure were compared to the unexposed cells maintained in culture medium for 24 h, in submerged conditions, in the incubator (Control cells) ».* **Same length of time after initial exposure to gases in all study groups.** |
| **Did the study employ appropriate statistical approaches?** | **++** | *«The optical density and cytokine concentrations of exposed cells were compared to the specific control with* ***Student’s t-test*** *and, if necessary with* ***Dunnett’s test****. Difference was significant if* *p < 0.05. Data represent at least three independent experiments each performed in triplicate».* |
| **Were all measured outcomes reported?** | **++** | *«Fig. 3.* ***Cell viability*** *of A549 cells after 30 min of FA-exposure». «…the cytokine productions in supernatants compared to the Control cells were significantly increased, by factors of 4, 7 and 2.5 respectively for IL-6, IL-8 and MCP-1 (Table 1).* ***Cytokines*** *were then evaluated in supernatants 24 h after air-exposure (Table 2a)».* |
| **Was the administered dose or exposure level adequately randomized?** | **++** | *«A* ***homogenous cellular monolayer*** *was observed at 20,000 cells/insert…».* |
| **Did the study assess the temporality of exposure and outcome?** | **++** | *«After 60 min of air-exposure a drying out of the apical side of the cell monolayer was observed by inversed microscopy. This result was confirmed by the XTT test…». «Cytokines were then evaluated in supernatants 24 h after air-exposure».* **Direct evidence that the exposure precedes the outcome and reasonable time lapse between exposure and outcome assessment.** |

***Persoz et al., 2012 (Tier 1)***

| **RoB criteria** | **RoB assessment** | **Evidence** |
| --- | --- | --- |
| **Can we be confident in the exposure characterization?** | **++** | *«Air and FA (50 µg/m3) atmospheres were generated as previously described (Persoz et al., 2010) and were delivered on the apical side of cells…».* |
| **Were experimental conditions identical across study groups?** | **+** | *«For all gas-exposure experiments cells were grown on inserts…». «Cells were exposed during 30 min to synthetic clean air or to FA at 50 µg/m3».* **No evidence of any difference in experimental conditions between exposure and control groups.** |
| **Can we be confident in the outcome assessment?** | **++** | *«Membrane integrity, evaluated by intracellular lactate dehydrogenase (LDH) release, was used as a viability test».* **Gold standard assay for measuring cytotoxicity.** *«The levels of cytokines produced by the macrophages in CM (IL-1β, TNFα and IL-8) and by the epithelial cells (IL-8 and MCP-1) were assessed by ELISA kits as previously reported».* **Gold standard assay for cellular inflammation. No evidence of different length of time before the outcome assessment between exposure groups.** |
| **Did the study employ appropriate statistical approaches?** | **++** | *«The LDH activity and cytokine concentrations of exposed cells were compared to the specific control with* ***Student’s t-test*** *and confirmed with* ***Mann-Whitney U test****. Difference was significant when p < 0.05».* |
| **Were all measured outcomes reported?** | **+** | *«After air or FA (at 50 µg/m3) exposure for 30 min, no toxicity was observed (data not shown) and the production of the two cytokines was similar for air and FA exposure (Fig. 1) ».* **Data for cytotoxicity assessment is not reported, but this should not appreciably bias results.** |
| **Was the administered dose or exposure level adequately randomized?** | **++** | *«Briefly after seeding at 40,000 cells/insert, and an incubation at 37 ◦C during 72 h, a* ***homogeneous cellular monolayer*** *with a confluence of 80–90% was obtained for BEAS-2B cells, as previously described for A549 cells».* |
| **Did the study assess the temporality of exposure and outcome?** | **++** | *«After toxicity evaluation, local inflammation was assessed by IL-8 and MCP-1 production 24 h after exposure».* **Direct evidence that the exposure precedes the outcome and reasonable time lapse between exposure and outcome assessment.** |

***Ritter et al., 2001 (Tier 2)***

| **RoB criteria** | **RoB assessment** | **Evidence** |
| --- | --- | --- |
| **Can we be confident in the exposure characterization?** | **++** | *«Dilutions of nitrogen dioxide in synthetic air were conducted through the exposure device and the contact with the membrane was* ***quantified*** *using the Saltzman reagent. The results for exposure concentrations in the range of* ***150 to 1200 ppb NO_2_*** *using 6 inserts in two exposure devices in parallel at each concentration are presented in figure 6».* |
| **Were experimental conditions identical across study groups?** | **+** | *«Immediately before exposure, the cell monolayers were washed twice with PBS and the inserts were moved into the exposure device (…). Cells were exposed to synthetic air (...), ozone or nitrogen dioxide using a gas flow rate of 25 ml/min per exposure device (…) or no gas was passed through the exposure unit. These cells were called “air/liquid” control and served as reference cells in the viability experiments».* **No evidence of any difference in experimental conditions between exposure and control groups.** |
| **Can we be confident in the outcome assessment?** | **+** | *«The cleavage of the tetrazolium dye (****WST-1 assay****; Boehringer, Mannheim, Germany) to its formazan was determined». «Intracellular contents of reduced and oxidized glutathione were determined using an extraction method with meta-phosphoric acid (HISSIN et al. 1976) followed by derivatisation with 2,4-dinitrofluorobenzene (REED et al. 1980) and chromatography using* ***HPLC-technique with UV-detection*** *of N-2,4-dinitrophenyl derivatives (MERTENS et al. 1991; YOSHIDA 1996) for analysis of both glutathione species in one chromatographical run».* **Reliable assays for cell viability and oxidative stress.** |
| **Did the study employ appropriate statistical approaches?** | **−−** | **No statistical analysis reported.** |
| **Were all measured outcomes reported?** | **++** | *«The viability of exposed cells increased dramatically by decreasing the gas flow rate (fig. 7) ». «These morphological changes indicating severe cellular damage were confirmed by the analysis of several intracellular parameters (fig. 11) ». «The results of the viability assays and the analysis of the intracellular glutathione and adenosinephosphates using Lk004 cells are presented in figure 12».* |
| **Was the administered dose or exposure level adequately randomized?** | **+** | *«Neither the individual differences of the gas distribution for each insert to the mean of three inserts (fig. 5, upper bars) nor the standard deviation over the three inserts (fig. 5, lower bars) were more than 5%».* **Same gas distribution for each insert and no evidence of selective administration to the different study groups.** |
| **Did the study assess the temporality of exposure and outcome?** | **++** | *«After a* ***postincubation period of 2 hours*** *inside an incubator under submersed conditions, the cells were characterized with regard to morphology, viability and the intracellular contents of glutathione (oxidized and reduced) as well as ATP and ADP».* |

***Liu et al., 2013 (Tier 1)***

| **RoB criteria** | **RoB assessment** | **Evidence** |
| --- | --- | --- |
| **Can we be confident in the exposure characterization?** | **+** | *«Since it was not possible to measure the VOC concentrations in all compartments of the dosing systems, a four compartment mass balance model was developed to estimate the absorbed air toxic concentration in the cells, medium components (mainly FBS) and fractions in water and air».* **The concentration of VOCs was not measured directly, but an alternative mass balance model was used.** |
| **Were experimental conditions identical across study groups?** | **+** | *«The cell cultures were maintained in a humidified incubator at 37 ◦C under 5% CO2 or directly in a 37 ◦C constant temperature room. A549 cells were subcultured according to the supplier’s instruction and were used for up to 20 passages».* **No differences reported between exposure and control groups for non-treatment related experimental conditions.** |
| **Can we be confident in the outcome assessment?** | **++** | *«Cell viability: CellTiter 96® AQueous Non-Radioactive* ***Cell Proliferation Assay*** *(Promega # G5430), CellTiter-Glo®». «DNA damage:* ***comet assay*** *kit (Trevigen, Cat#4250-050-K, Gaithersburg, MD, USA)». «Interleukin 8 expression:* ***AlphaLISA®*** *IL8 kit (PerkinElmer® #AL224 C, Massachusetts, USA)».* **Gold standard assays for all the outcomes.** |
| **Did the study employ appropriate statistical approaches?** | **++** | *«Given that the fitted EC50 values are log-normally distributed, significant differences between treatments were analysed using one-way ANOVA or Student’s t-Test with log-transformed concentration. P values were reported whenever applicable».* |
| **Were all measured outcomes reported?** | **++** | *«Full concentration effect curves were obtained for all endpoints (Fig. 7) and the EC50 values were lower for DNA damage and IL8 expression than for cell viability confirming the higher sensitivity of sub lethal endpoints».* |
| **Was the administered dose or exposure level adequately randomized?** | **+** | **No evidence of selective administration of exposure dose.** |
| **Did the study assess the temporality of exposure and outcome?** | **++** | *«Exposures were carried out for 1 h and 24 h. The cell viability was then assessed by the* ***in situ MTS assay****». «Not only cell viability could be assessed but also sub lethal biological endpoints such as* ***DNA damage*** *and* ***interleukin expressions****».* |

***Mirowsky et al., 2016 (Tier 1)***

| **RoB criteria** | **RoB assessment** | **Evidence** |
| --- | --- | --- |
| **Can we be confident in the exposure characterization?** | **+** | *«The cells were then exposed to four different concentrations of nitrogen dioxide (1 ppm, 2 ppm, 3 ppm, or 5 ppm) or ozone (0.25 ppm, 0.50 ppm, 0.75 ppm, or 1.00 ppm) for 2 h in exposure chambers held at 37.5 ºC, 5% CO2 and 88% relative humidity».* **Indirect evidence of exposure characterization using methods that directly measure gas concentrations.** |
| **Were experimental conditions identical across study groups?** | **++** | *«Prior to each exposure the apical surface of each Transwell was washed with Dulbecco’s phosphate buffered saline (Life Technologies, Grand Island, NY) and fresh media was added into the basolateral compartment».* **No differences reported between exposure and control groups for non-treatment related experimental conditions.** *«Simultaneous to each NO2 and O3 exposure, additional cells were exposed to filtered air at the same temperature, humidity and air flow rate that was used for the pollutant exposures; this served as our negative control».* **Direct evidence of identical experimental conditions.** |
| **Can we be confident in the outcome assessment?** | **+** | *«The Stress and Toxicity PCR Array covered genes from multiple pathways, including DNA damage, hypoxia, inflammation, oxidative stress, and heat shock. This PCR array was selected to give a wide overview of various and unique pathways that may be influenced by O3 or NO2 exposures, which could ultimately be used to narrow down specific pathways and genes of interest».* **RT-PCR is a reliable technique for the assessment of all these outcomes, but it is not the gold standard.** |
| **Did the study employ appropriate statistical approaches?** | **++** | *«All data were tested for normality using the* ***Kolmogorov–Smirnov test*** *and then analyzed using* ***one-factor analysis of variance (ANOVA)****. To test for comparisons between filtered air and the various concentrations of each gas, Tukey’s Multiple Comparison Test was used. Statistical significant was set for a p value <0.05».* |
| **Were all measured outcomes reported?** | **++** | *«Figure 2. Gene expression heat maps generated using a Human Oxidative Stress and Antioxidant Defense RT2 Profiler PCR Array (SABiosciences). A total of 84 genes were assessed (…) ». «Figure 3. mRNA expression of IL-8 following 2 h long exposures of HBECs to various concentrations of O3 and NO2 (…) ». «Figure 4. mRNA expression of PTGS2 following 2 h long exposures (…) ».* |
| **Was the administered dose or exposure level adequately randomized?** | **+** | **No evidence of different administration of exposure dose between groups** |
| **Did the study assess the temporality of exposure and outcome?** | **++** | *«Figure 5. mRNA expression of IL-6 following 2 h long exposures of HBECs to various concentrations of O3 and NO2 at (A) 1 h post exposure and (B) 4 h post exposure».* |

***Gosepath et al., 2000 (Tier 1)***

| **RoB criteria** | **RoB assessment** | **Evidence** |
| --- | --- | --- |
| **Can we be confident in the exposure characterization?** | **+** | *«Cells were then exposed to synthetic air (5% CO2) or with synthetic air enriched with ozone in 3 different concentrations (100, 500, or 1000 µg/m3) for 4 weeks at 37°C».* **Indirect evidence of exposure characterization using methods that directly measure gas concentrations.** |
| **Were experimental conditions identical across study groups?** | **+** | *«Culture medium was exchanged 3 times a week and those of one week were collected and stored at -20°C. Cell cultures from each patient were exposed to synthetic air and all 3 concentrations of ozone to have a maximum of comparability of the data obtained. ».* **No evidence of different experimental conditions between synthetic air group (control) and ozone-enriched group.** |
| **Can we be confident in the outcome assessment?** | **++** | *«CBF was calculated using video interference contrast microscopy at 10 different sites of each specimen. Quantitative assessment of cytokines (IL-8, IL-4, g-INF) was performed using commercially available sandwich-ELISAs (R&D Systems, Wiesbaden, Germany). Cytotoxicity and cellular viability were monitored with a commercially available test for LDH activity».* **Gold standard assays for all the outcomes.** |
| **Did the study employ appropriate statistical approaches?** | **++** | *«For the statistical analysis of the data obtained a Wilcoxon signed-ranked test was used and results were statistically significant if p was less than 0.05 and marked with an asterisk (*) in the diagrams used for illustration».* |
| **Were all measured outcomes reported?** | **++** | *«Figure 1. IL-8 release and CBF after exposure to synthetic air (…) ». «Figure 2. LDH activity. cytotoxicity. and viability after exposure to synthetic air». «Figure 3. CBF in unaffected and chronically inflamed mucosa after ozone exposure». «Figure 4. IL-8 release in unaffected and chronically inflamed mucosa after ozone exposure». «Figure 5, LDH activity, cytotoxicity, and viability after ozone exposure».* |
| **Was the administered dose or exposure level adequately randomized?** | **+** | *«Cells were then exposed to synthetic air (5% CO2) or with synthetic air enriched with ozone in 3 different concentrations (100, 500, or 1000 µg/m3) for 4 weeks at 37°C».* **No evidence of selective administration of the exposure level between groups.** |
| **Did the study assess the temporality of exposure and outcome?** | **++** | *«After the first week of exposure ozone did not have any significant impact on the CBF in healthy or chronically inflamed mucosal tissue (…) There was also no effect on CBF after 2 to 4 weeks of exposure at an ozone concentration of 100 µg/m3 (…) ». «There was no effect on the IL-8 release of healthy mucosa after 1 week of exposure at all 3 concentrations nor of chronically inflamed mucosa at 100 µg/m3 Between the second and the fourth week IL-8 release was significantly reduced at all three concentrations (…) ».* |

***Guenette et al., 2022 (Tier 1)***

| **RoB criteria** | **RoB assessment** | **Evidence** |
| --- | --- | --- |
| **Can we be confident in the exposure characterization?** | **++** | *«We generated a test atmosphere of up to 1 ppm ozone using an ozone feedback control loop described elsewhere (Guenette et al. 1997). The analyzer measured the concentration of ozone exiting the CelTox system and adjusted the flow of ozone introduced before the tee, using the tee to bleed off any excess pressure».* |
| **Were experimental conditions identical across study groups?** | **++** | *«For all experiments, cells were maintained in parallel in the incubator to serve as controls, with and without apical media. An additional control involved inclusion in adjacent wells within the ALI exposure system cells that were fully submerged in culture media (i.e. media present in apical and basolateral compartments) ».* |
| **Can we be confident in the outcome assessment?** | **+** | *«To determine whether the ALI exposure conditions maintain the viability of A549 lung epithelial cells during exposure, we conducted the resazurin reduction assay on cells exposed for 2 h to clean air at the ALI».* **Reliable assay to measure cell viability.** |
| **Did the study employ appropriate statistical approaches?** | **++** | *«Resazurin reduction assay data from the 2 h clean air and ozone exposure experiments were analyzed by two-way ANOVA with Treatment and Time as factors, followed by the Holm-Sidak multiple comparison procedure (…) The data were transformed as required to meet the assumptions of normality and homoscedasticity ».* |
| **Were all measured outcomes reported?** | **++** | *«Figure 3. Viability (metabolic activity) of A549 cells exposed to clean air at the ALI for 2 h. Resazurin reduction by A549 cells was measured over 4 h immediately after exposure».*  *«Viability (metabolic activity) of A549 cells exposed to 200 ppb ozone at the ALI for 2 h, as measured over a 3-hour time period immediately post-exposure».* |
| **Was the administered dose or exposure level adequately randomized?** | **+** | **Indirect evidence that ozone exposure levels were not administered selectively to A549 cells based on the different exposure groups** |
| **Did the study assess the temporality of exposure and outcome?** | **++** | *«Figure 3. Viability (metabolic activity) of A549 cells exposed to clean air at the ALI for 2 h. Resazurin reduction by A549 cells was measured over 4 h immediately after exposure».*  *«Viability (metabolic activity) of A549 cells exposed to 200 ppb ozone at the ALI for 2 h, as measured over a 3-hour time period immediately post-exposure».* |

***Gostner et al., 2016 (Tier 1)***

| **RoB criteria** | **RoB assessment** | **Evidence** |
| --- | --- | --- |
| **Can we be confident in the exposure characterization?** | **++** | *«The exposure platform (Fig. 1A) was designed to enable optimal treatment of cells with a humidified atmosphere containing a volatile analyte, formaldehyde, in a defined concentration».* |
| **Were experimental conditions identical across study groups?** | **++** | *«For the airborne exposure experiments, A549 cells were grown to confluence and lifted to ALI. (…) Treatment conditions were adjusted at the exposure platform at least 4hr before the start of the experiments so that temperature, humidity and analyte concentration were balanced until the cells were placed into the exposure or reference chamber. ALI cultures were exposed to atmospheres containing 0, 0.1, and 0.5ppm formaldehyde for 3 days».* |
| **Can we be confident in the outcome assessment?** | **+** | *«Cell viability was estimated as a primary endpoint after exposure, using a metabolic activity assay based on the reduction of resazurin».* **Reliable assay to measure cell viability.** *«To explore cellular changes of A549 ALI cultures exposed to sublethal concentrations of formaldehyde, we performed a transcriptional analysis».* **Reliable assay to identify genotoxic effects of formaldehyde through differentially expressed transcripts** |
| **Did the study employ appropriate statistical approaches?** | **++** | *«In addition to the eBayes approach, which uses a modified t-statistics that is advantageous for a small number of biological replicates, we applied analysis of variance (ANOVA) followed by Tukey’s HSD post-hoc test to consider the variance of the different controls (0ppm exposure control and reference chamber control) ».* |
| **Were all measured outcomes reported?** | **++** | *«Figure 3. (B) After 3 days, the* ***viability*** *of ALI A549 cultures exposed to gaseous formaldehyde at 0.1ppm (and 0.5ppm) was determined in comparison to cells cultivated in the reference chamber. Mean values±SEM of 3 experiments are presented». «Figure 4. Analysis of* ***differentially expressed transcripts*** *due to formaldehyde exposure».* |
| **Was the administered dose or exposure level adequately randomized?** | **+** | *«ALI cultures were placed in the middle rows of 24-well plates; wells at the edges were filled with medium only. (…) ALI cultures were exposed to atmospheres containing 0, 0.1, and 0.5ppm formaldehyde for 3 days».* **Indirect evidence that the different formaldehyde exposure levels were not administered selectively to A549 cells.** |
| **Did the study assess the temporality of exposure and outcome?** | **++** | *«Figure 3. (B)* ***After 3 days****, the viability of ALI A549 cultures exposed to gaseous formaldehyde at 0.1ppm (and 0.5ppm) was determined in comparison to cells cultivated in the reference chamber. Mean values±SEM of 3 experiments are presented».* |

***Zavala et al., 2016 (Tier 1)***

| **RoB criteria** | **RoB assessment** | **Evidence** |
| --- | --- | --- |
| **Can we be confident in the exposure characterization?** | **++** | *«During all exposures, NO and NO2 levels were measured using a Teledyne model 9841 NOx analyzer (Teledyne Monitor Labs, Englewood, CO) while O3 was measured with a Teledyne model 9811 monitor».* |
| **Were experimental conditions identical across study groups?** | **+** | **It can be assumed that non-treatment-related experimental conditions were identical across study groups** |
| **Can we be confident in the outcome assessment?** | **++** | *«Cytotoxicity was assessed via LDH concentrations measured in the basolateral medium and the apical washes using a cytotoxicity kit». «Interleukin-6 (IL-6) and IL-8 protein release in the basolateral medium and apical surfaces were measured to determine the inflammatory response via enzyme-linked immunosorbent assay kits».* **Gold standard assays for both outcomes.** |
| **Did the study employ appropriate statistical approaches?** | **++** | *«Data from independent sets of exposures were combined and analyzed using ANOVA followed by Tukey post-test where differences were considered significant if p ≤ 0.05».* |
| **Were all measured outcomes reported?** | **++** | *«Figure 5. Cytotoxicity results at 9 and 24 h post-exposure for (A) apical washes and (B) basolateral medium». «Figure 6. IL-8 expression results at 9 and 24 h post-exposure for (A) apical washes and B) basolateral medium».* *«Figure 7. IL-6 expression results at 9 and 24 h post-exposure for (A) apical washes and (B) basolateral medium».* |
| **Was the administered dose or exposure level adequately randomized?** | **+** | *«To evaluate whether the type of atmosphere contributed to differences in biological response in two cell models, we exposed cells to a single pollutant or a complex gaseous mixture».* **Indirect evidence that the exposure dose was not administered selectively based on the different cell types.** |
| **Did the study assess the temporality of exposure and outcome?** | **++** | *«Basolateral supernatants and apical washes with 1 mL of DPBS were collected at 9 and 24 h post-exposure for each exposure condition».* |

***Sayyed et al., 2022 (Tier 1)***

| **RoB criteria** | **RoB assessment** | **Evidence** |
| --- | --- | --- |
| **Can we be confident in the exposure characterization?** | **++** | *«Then the gasoline concentration in ppm was calculated with the following equation (…) ».* |
| **Were experimental conditions identical across study groups?** | **+** | *«The whole detachable snapwell insert seeded with cells overnight was transferred to the glass chamber for static exposure. During exposure experiments, we used the ALI (air-liquid interface) culture system. Cell cultures were exposed to gasoline vapors from their apical side and were nourished from their basolateral side with cell media».* **No evidence of any difference in these experimental conditions between groups.** |
| **Can we be confident in the outcome assessment?** | **++** | *«The tetrazolium compound* ***MTS*** *[3-(4,5-dimethylthiazol-2-yl)-5-(3-carboxymethoxyphenyl)-2-(4-sulfophenyl)-2H-tetrazolium] combined with the electron coupling agent PMS (phenazine methosulfate), was bio-reduced by metabolically active viable cells to soluble formazan». «Lactate dehydrogenase (****LDH****) is a cytosolic enzyme released from cells upon cell membrane damage, a good indicator of cell membrane integrity and cytotoxicity». « (…) human IL-1β and TNF-α release were quantified using* ***ELISA*** *kits». «The* ***comet assay*** *investigated DNA damages». «The assessment of mechanism of cell death whether through apoptosis or necrosis was conducted using the* ***Annexin/PI flow cytometry assay****».* **Gold standard assays for all the outcomes** |
| **Did the study employ appropriate statistical approaches?** | **++** | *«The built-in analysis of variance (ANOVA) was adopted followed by Dunnett’s post-hoc test, Newmen-Keuls or F-test. The criterion of significance was p< 0.05. Half maximal inhibitory concentration (IC50) values were by nonlinear regression based on the four-parameter logistic function and statistical analysis».* |
| **Were all measured outcomes reported?** | **++** | *«Fig. 2. MTS viability assay and cell rounding assay». «Fig. 3. LDH cytotoxicity assay».* *«Fig. 4. The release of pro-inflammatory cytokines Il-1β and TNF-α». «Fig. 5. Evaluation of DNA damage by comet assay». «Fig. 6. Gasoline induced apoptosis by flow cytometry».* |
| **Was the administered dose or exposure level adequately randomized?** | **+** | *«The cells were exposed to various airborne of gasoline-VOCs directly at the ALI for 1 h at 37 ◦C. Once the exposure ended, inserts were returned to their initial plate. Media was added to both sides and cells were incubated until further toxicity assessments».* **No evidence of selective administration of the different exposure levels in the cell cultures.** |
| **Did the study assess the temporality of exposure and outcome?** | **++** | *«The toxicity of emitted gasoline-VOCs in A549 cells was assessed using several in vitro cytotoxicity assays selected to measure critical biological endpoints such as cell mitochondrial activity (cell viability), membrane integrity, DNA damages in addition to inflammatory cytokines release response* ***immediately and 24 h post-exposure****».* |

***Kastner et al., 2013 (Tier 1)***

| **RoB criteria** | **RoB assessment** | **Evidence** |
| --- | --- | --- |
| **Can we be confident in the exposure characterization?** | **++** | *«Fig. 2 shows the record of a real-time measurement of the two pollutants during the generation of a mixture of 200 µg m-3 HCHO and 800 µg m-3 NO2, which are the highest target concentrations of each pollutant in our study».* |
| **Were experimental conditions identical across study groups?** | **+** | *«*The chamber was placed in an incubator, in order to keep the cells at 37 ºC during the course of exposures*».* **No evidence of any difference in experimental conditions between groups.** |
| **Can we be confident in the outcome assessment?** | **++** | *«Viability of cells exposed to pollutants or synthetic air in plates or inserts was assessed by the* ***MTT*** *assay (…) ». «Cell necrosis was assessed by measuring the release of lactate dehydrogenase (****LDH****) (…) ». «The development or the disruption of a tight epithelial cell monolayer was determined by measuring the TEER with an ohmmeter and chopstick electrodes (…) ». «The cytokine IL-6 and the chemokine IL-8 were quantified in the cell culture supernatants using enzyme-linked immunosorbent assay (****ELISA****) kits (…) ».* **Gold standard assays for all the outcomes** |
| **Did the study employ appropriate statistical approaches?** | **++** | *«Statistical differences between the different groups were analyzed by one-way analysis of variance (ANOVA) followed by the Bonferroni’s post-test using the GraphPad Prism 4 software. Data were considered as statistically different when p < 0.05».* |
| **Were all measured outcomes reported?** | **++** | *«Fig. 3. MTT metabolism, LDH release, and IL-6 and IL-8 secretion by Calu-3 cells after single or repeated exposures to synthetic air». «Fig. 4. TEER of Calu-3 cell monolayers after exposure to synthetic air at the ALI». «Fig. 5. MTT metabolism, LDH release, and IL-6 and IL-8 secretion by Calu-3 cells after single or repeated exposures to gaseous pollutants».* |
| **Was the administered dose or exposure level adequately randomized?** | **+** | *«*These concentrations were reached within the 10 min that followed the beginning of the generation and remained stable. The chamber was then opened for 4 s to insert cell plates, inducing a 48% and 23% decrease in the NO2 and HCHO concentrations, respectively*».* **No evidence of selective administration of the different exposure levels in the cell cultures.** |
| **Did the study assess the temporality of exposure and outcome?** | **++** | *«TEER was monitored just before the exposures (t = 0 h), at t = 4 h and at t = 24 h». «MTT metabolism (…), LDH release (…) and IL-6 (…) and IL-8 (...) secretions were assessed 24 h after the beginning of the single or the last repeated exposure, as described in the material and methods section».* |

***Bakand et al., 2006 (Tier 1)***

| **RoB criteria** | **RoB assessment** | **Evidence** |
| --- | --- | --- |
| **Can we be confident in the exposure characterization?** | **++** | *«Accurate flow rates for both NO2 gas and diluent air was monitored using calibrated rotameters (SKC, USA) and directed to the dilution chamber in order to produce the final desired concentrations».* |
| **Were experimental conditions identical across study groups?** | **+** | *«After washing the cell monolayer with HBSS, membranes were detached from their holders and were placed into the horizontal diffusion chambers containing culture media without FCS, supplemented with 1% antibiotics and HEPES buffer (0.01 M). The upper compartment of the diffusion chambers were closed and standard atmospheres of NO2 were delivered (…) ».* **No evidence of any difference in experimental conditions between groups.** |
| **Can we be confident in the outcome assessment?** | **++** | *«The Promega CellTiter 96® AQueous Non-Radioactive Cell Proliferation Assay was used to measure the cytotoxicity of test gas (…) ».* **MTS assay** *«The Neutral red (3-amino-7-dimethyl-amino-2-methylphenazine hydrochloride) uptake (****NRU****; Sigma) assay is a cell survival/viability technique based on the ability of viable cells to incorporate and bind supravital neutral red dye (…). This assay was used to measure the cytotoxicity of NO2». «****ATP*** *content was measured using the CellTiter-Glo® Luminescent* ***Cell Viability Assay*** *(…) ».* **Reliable assays for all the cell viability and cytotoxicity endpoints.** |
| **Did the study employ appropriate statistical approaches?** | **++** | *«After testing the homogeneity of variances using the F-test, the Student’s t-test was used to compare the average cell viability of exposed cells and control cells. Differences were considered as statistically significant at p < 0.05».* |
| **Were all measured outcomes reported?** | **++** | *«Cell viability of human cells exposed to two different flow rates of synthetic air during 1 h exposure time using the MTS assay are presented in Table 2». «Cytotoxic effects of NO2 on human A549 lung derived cell lines and skin fibroblasts are presented in Figs. 4 and 5, respectively».* |
| **Was the administered dose or exposure level adequately randomized?** | **+** | *«The upper compartment of the diffusion chambers were closed and standard atmospheres of NO2 were delivered through the chambers with a low flow rate (25 ml/min) for 1 h using calibrated rotameters and an air sampling pump (SKC, USA) ».* **No evidence of selective administration of the different NO_2_ levels in the cell cultures.** |
| **Did the study assess the temporality of exposure and outcome?** | **++** | *«****After the incubation period****, aliquots of 40 µl from the top of the membranes were transferred (…) and absorbance was recorded against controls at 492 nm (…).* (**MTS assay**)  *«****After exposure****, fresh culture media was added to the bottom (…). The NRU solution was prepared (…). The plate was shaken for 10 min using an orbital mixer (Ratek Instruments, Australia) and aliquots of 100 µl were transferred (…) The absorbance was recorded at 540 nm (…) ».* (**NRU assay**)  *«****After exposure****, fresh culture media was added to the bottom (…) Aliquots of 100 µl from the top part of the membrane were transferred (…) and the luminescence level was recorded using a luminometer (…) ». (****ATP assay****)* |

***McCullough et al., 2014 (Tier 1)***

| **RoB criteria** | **RoB assessment** | **Evidence** |
| --- | --- | --- |
| **Can we be confident in the exposure characterization?** | **++** | *«Ozone was generated with a Measurement Technologies Precision Ozone Generator and the O3 concentration in the exposure system was monitored with a Thermo Environmental 49i analyzer».* (Supplemental Materials and Methods) |
| **Were experimental conditions identical across study groups?** | **++** | *«Two hours before pHBEC exposure, the apical surface of each insert was washed with Dulbecco’s PBS (Life Technologies, Grand Island, NY), and the medium in the basolateral compartment was replaced with fresh ALI medium. Two hours before BEAS-2B exposure experiments, the apical growth medium was removed, the apical surface was washed with Dulbecco’s PBS, and the medium in the basolateral compartment was replaced with fresh keratinocyte growth medium».* **Identical experimental conditions between different groups.** |
| **Can we be confident in the outcome assessment?** | **++** | *«Quantitative* ***PCR*** *was performed using TaqMan primer/probe sets for the indicated genes. Equal amounts of total protein were loaded on SDS-PAGE gels, electrophoresed, and electroblotted onto nitrocellulose membranes».* (**Western Blot**) **Gold standard assays for cellular inflammation and oxidative stress.** |
| **Did the study employ appropriate statistical approaches?** | **++** | *«Statistical analysis with a one-way ANOVA with repeated measures with a 95% confidence interval. Post testing was conducted with Tukey’s multiple comparison test. Comparisons were considered to be statistically significant at P < 0.05».* |
| **Were all measured outcomes reported?** | **++** | *«Figure 1.* ***Induction of proinflammatory cytokines*** *in primary human bronchial epithelial cells (pHBECs) in response to 0.5 ppm O3 exposure». «Figure 2. Indicators of canonical* ***NF-kB pathway activation*** *are present in BEAS-2B cells exposed to 0.5 ppm O3 but not in pHBECs». «Figure 3. Epidermal growth factor receptor (****EGFR****),* ***MEK1/2****, and* ***ERK1/2*** *are activated in cells exposed to 0.5 ppm O3». «Figure 4.* ***MKK4*** *and* ***p38*** *are activated in pHBECs exposed to 0.5 ppm O3». «Figure 5.* ***Phosphorylation*** *of the mitogen-activated protein kinases* ***ERK1/2*** *and* ***p38*** *in pHBECs exposed to 0.5 ppm O3 in the presence of small molecule kinase inhibitors».* |
| **Was the administered dose or exposure level adequately randomized?** | **+** | *«The cells were maintained in ALI culture for 4 to 5 days before use in exposure experiments, which allowed the epithelial cells to become confluent and polarized». «O3 exposure time course and small molecule inhibitor treatment experiments were conducted in pHBECs from seven and six individual donors, respectively».*  **No evidence of selective administration in ozone exposures.** |
| **Did the study assess the temporality of exposure and outcome?** | **++** | *«****At the completion of the 2-hour exposure****, the mean (6 SEM) induction of IL-8, IL-6, IL-1a, and IL-1β was (…) ». «Phosphorylation of the IKKa and -β (serine residues 176 and 180) increased (…)* ***after 30, 60, and 120 minutes*** *of O3 exposure, respectively».* |

***McCullough et al., 2016 (Tier 1)***

| **RoB criteria** | **RoB assessment** | **Evidence** |
| --- | --- | --- |
| **Can we be confident in the exposure characterization?** | **++** | *«Cells were then placed in the US EPA Environmental Public Health Division’s in vitro exposure chambers (****McCullough et al., 2014****) and exposed to either clean air (control) or 0.5 ppm ozone for 2 h as described previously (McCullough et al., 2014; Ross et al., 2007; Wu et al., 2011) ».* |
| **Were experimental conditions identical across study groups?** | **+** | *«After collection, cells were expanded by passage in culture and plated at air–liquid interface (ALI) on 24 mm uncoated transwell inserts with 0.4 mm pores (Corning) as previously described (Ross et al., 2007). Prior to exposure, cells were maintained in ALI culture for 4 days, which allowed them to become confluent and polarized».* **No evidence of different experimental conditions between groups.** |
| **Can we be confident in the outcome assessment?** | **++** | *«Immediately after exposure cells were removed from the chambers and total RNA was harvested with PureLink RNA Mini Kit (Life Technologies) according to the manufacturer’s protocol. In parallel, unexposed chromatin immunoprecipitation (ChIP) samples were collected at the same time as exposed cells that were harvested for RNA».* |
| **Did the study employ appropriate statistical approaches?** | **++** | *«The normality of the distribution of both basal and induced gene expression values was determined with the D’Agostino-Pearson omnibus normality test (…). The relationship between baseline abundance of the indicated chromatin modifications and either basal or induced expression of the indicated genes was determined by simple linear regression. Sensitivity analysis was conducted on all comparisons following the exclusion of any donors whose gene expression values were determined to be outliers according to the Grubb’s test».* |
| **Were all measured outcomes reported?** | **++** | *«FIG. 2. Correlations between specific baseline chromatin modification levels and basal gene expression. The abundance of IL-8 (A), IL-6 (B), HMOX1(C) in unstimulated pHBEC was determined as a percentage of ACTB expression and compared to baseline levels of H3K4me3, H3K27ac, and H4ac». «FIG. 3. Correlations between specific baseline chromatin modification levels and O3-induced gene expression. (A) Induction of the pro-inflammatory genes COX-2, IL-8, and IL-6 and the oxidative stress gene HMOX1 were measured in pHBEC immediately following a 2-h exposure to 0.5 ppm O3. Baseline levels of H3K4me3, H3K27me2/3, and 5-hmC were compared to the postexposure induction of HMOX1 (B) and COX2 (C), in pHBEC».* |
| **Was the administered dose or exposure level adequately randomized?** | **+** | *«Cells were then placed in the US EPA Environmental Public Health Division’s in vitro exposure chambers (McCullough et al., 2014) and exposed to either clean air (control) or 0.5 ppm ozone for 2 h as described previously (McCullough et al., 2014; Ross et al., 2007; Wu et al., 2011».* **Indirect evidence that each cell had an equal chance to be assigned to any group.** |
| **Did the study assess the temporality of exposure and outcome?** | **++** | *«Induction of the pro-inflammatory genes COX-2, IL-8, and IL-6 and the oxidative stress gene HMOX1 were measured in pHBEC* ***immediately following a 2-h exposure*** *to 0.5 ppm O3».* |

***Verstraelen et al., 2021 (Tier 1)***

| **RoB criteria** | **RoB assessment** | **Evidence** |
| --- | --- | --- |
| **Can we be confident in the exposure characterization?** | **++** | *«Generated concentrations were determined by combining microbalance consumption and used airflows on the one hand and a flame ionization detector (FID) analyzer (J.U.M. Engineering 3-300A, Karlsfeld, Germany) on the other hand».* |
| **Were experimental conditions identical across study groups?** | **++** | *«Before cells were exposed to EB vapors, the ALI system was thoroughly tested for uniformity with clean air (CA) and nitrogen dioxide (NO2) exposure. At least 42 positions with A549 inserts in the plate were exposed to either CA (negative control) or NO2 (in-house positive control, about 12 ppm) at a flow of 500 mL/min in the main distribution line. Experiments were repeated three times using cells of different passages».* **Direct evidence that experimental conditions were identical for all study groups.** |
| **Can we be confident in the outcome assessment?** | **++** | *«We performed the MTT (measurement of mitochondrial activity) and lactate dehydrogenase (LDH) assay (measurement of membrane integrity) ». «Here, C-C motif chemokine ligand 2 (CCL2), interleukin (IL)6, IL8, superoxide dismutase (SOD)2, and heme oxygenase (HMOX)1 were analyzed by qRT-PCR and/or enzyme-linked immunosorbent assay (ELISA) ».* **Gold standard assays for all the outcomes.** |
| **Did the study employ appropriate statistical approaches?** | **++** | *«Fold changes (FC) relative to CA were calculated and logarithmically transformed (log2 scale) prior to statistical analysis. Significant changes relative to CA were assessed by mixed models while considering experiment ID (biological replicate) as random factor». «P-value smaller than 0.05 was used as cut-off for statistical significance, and abs(mean(log2 FC)) above log2(1.5) was used to focus on biologically relevant expression changes». «Significant changes in cytokine concentration were analyzed relative to CA using ANOVA».* |
| **Were all measured outcomes reported?** | **++** | *«Fig. 3: Change in cell viability (as % compared to clean air (CA)) of A549 cells after 4 h exposure to ethylbenzene (EB, nominal concentration 30,000, 40,000, and 50,000 mg/m3), NO2 (nominal concentration 20 ppm), and incubator controls (IC) based on 5 independent biological experiments».*  *«Fig. 4: Change in cytotoxicity (as % compared to clean air (CA)) of A549 cells after 4 h exposure to ethylbenzene (EB, nominal concentration 30,000, 40,000, and 50,000 mg/m3), NO2 (nominal concentration 20 ppm) and incubator controls (IC) based on 5 independent biological experiments».*  *«Fig. 5: Interleukin (IL)6 (A), IL8 (B), and C-C motif chemokine ligand 2 (CCL2) (C) gene expression, and IL-8 secretion normalized according to cell viability (MTT) (D) and cytotoxicity (LDH) (E) as compared to clean air (CA) in A549 cells».* |
| **Was the administered dose or exposure level adequately randomized?** | **+** | *«The measured NO2 and EB exposure concentrations for the 5 independent cell culture exposure runs can be found in Table 3. The data show a reproducible generation method with a coefficient of variation <5% for all tested concentrations».* **Indirect evidence of adequate randomization of exposure concentrations using independent biological replicates.** |
| **Did the study assess the temporality of exposure and outcome?** | **++** | *«A possible reason for a lesser effect on cells compared to MTT might be that we measured LDH release too late (after 20 h post-incubation), since LDH has a half-life of approximately 9 h in CCM, and NO2 might induce cell death faster than EB».* |

***Mascelloni et al., 2015 (Tier 1)***

| **RoB criteria** | **RoB assessment** | **Evidence** |
| --- | --- | --- |
| **Can we be confident in the exposure characterization?** | **++** | *«The gas delivery to the cells was regulated by mass flow controllers (MFCs) (Brooks Instrument, NL). One 500 sccm (standard cubic centimetres/ minute) MFC was used to regulate the synthetic air (BOC gases, UK) flow, and a 100 sccm MFC was used to regulate the benzene flow (1 ppm balanced in nitrogen, BOC gases, UK). A total flow rate of 30 ml/min was used, split between the three wells, with a theoretical flow rate of 10 ml/min in each well».* |
| **Were experimental conditions identical across study groups?** | **+** | *«Each exposure experiment was accompanied by a set of incubator controls, which consisted in a 6-well plate left for 2 h in the incubator, where 3 wells contained cells with media (Incubator Media), and 3 wells contained cells with the media removed (Incubator Dry). The removal of the media from the 3 wells was considered the start point of the 2 h incubation of the incubator controls».* **Indirect evidence of identical experimental conditions across study groups.** |
| **Can we be confident in the outcome assessment?** | **++** | *«Reactive oxygen species were assessed by measuring the oxidation of the redox sensitive dye 20,70-dichlorodihydrofluorescein diacetate (****H2DCF-DA****, Sigma–Aldrich, UK) ». «DNA strand breaks were assessed by the alkaline* ***comet assay*** *(Singh et al., 1988) ».* |
| **Did the study employ appropriate statistical approaches?** | **++** | *«The means were compared using* ***ANOVA*** *with* ***Bonferroni post-hoc*** *comparisons».*  *«The ratios were compared by a* ***Kruskal–Wallis*** *independent samples test, which retained the null hypothesis of the samples having the same distribution (p = 0.102). An independent samples median test also retained the null hypothesis, suggesting that the medians of the ratios are similar (p = 0.354) ».*  *«Tail intensities of each exposed sample were compared with its relative air exposed control using Friedman’s 2-way* ***ANOVA*** *by ranks».* |
| **Were all measured outcomes reported?** | **++** | *«Fig. 3. Summary of the DCF fluorescence measurements before and after exposure».*  *«Fig. 4. Mean tail intensity (%) of the control and exposed cells».*  *«Fig. 5. Graphical representation of the % tail intensities measured in the Comet assay as a function of benzene concentration».* |
| **Was the administered dose or exposure level adequately randomized?** | **+** | *«For each exposure experiment, 3 Transwell inserts cells from the same 6-well plate were exposed for 2 h to synthetic air (controls). Subsequently, the remaining 3 Transwell inserts were exposed to one of three different concentrations of benzene (0.03 ppm; 0.1 ppm; 0.3 ppm), which were reported to have cytotoxic effects».* **No evidence of selective administration of the different benzene concentrations to any specific group.** |
| **Did the study assess the temporality of exposure and outcome?** | **++** | *«Cells were analysed for levels of ROS* ***before and after the exposure*** *together with a control that was exposed only to synthetic air and a set of incubator controls both with and without apical medium».*  *«****Following exposure of cells to air*** *(controls), low (0.03 ppm) and medium (0.1 ppm) benzene concentrations, the incidence of highly damaged hedgehog cells was below 15% of the total number of cells counted».* |

**Table S2. Study quality/risk of bias criteria for *in vitro* epidemiology studies evaluating the effect of urban aerosols and PM on human airway ALI cultures.**

|  | **Key criteria** | | | **Other quality criteria** | | | |
| --- | --- | --- | --- | --- | --- | --- | --- |
|  | **Can we be confident in the exposure characterization?** | **Were experimental conditions identical across study groups?** | **Can we be confident in the outcome assessment?** | **Did the study employ appropriate statistical approaches?** | **Were all measured outcomes reported?** | **Was the administered dose or exposure level adequately randomized?** | **Did the study assess the temporality of exposure and outcome?** |
| **Definitely low RoB (++)** | There is direct evidence that the exposure was characterized using methods that directly measure aerosol or PM exposure levels, and that exposure was consistently administered with the same method and timeframe across treatment groups. | There is direct evidence that cell culture conditions and other non-treatment-related experimental conditions (e.g., incubator and plate conditions, medium change schedule and washes, solvents used) were identical across study groups, including controls. | There is direct evidence that the outcomes were assessed using well-established methods (the gold standard), and at the same length of time after initial exposure to aerosols or PM in all study groups. | There is direct evidence that the study employed appropriate statistical methods. | There is direct evidence that all measured outcomes that are relevant for the study (outlined in the protocol, methods, abstract, and/or introduction)  have been reported. | There is direct evidence that the exposure dose was not administered selectively based on the different cell types or tissues, and a homogeneous cell suspension was obtained. Therefore, each cell had an equal chance to be assigned to any study group, including controls. | There is direct evidence that the exposure precedes the outcome, and a  reasonable time  between exposure and outcome measurement was  examined. |
| **Probably low RoB (+)** | There is indirect evidence that the exposure was characterized using methods that directly measure aerosol or PM exposure levels, and that exposure was consistently administered with the same method and timeframe across treatment groups. | There is indirect evidence that non-treatment-related experimental conditions were identical across study groups, including controls, or it can be assumed if authors did not report any differences. | There is indirect evidence that the outcomes were assessed using valid and reliable methods (but not the gold standard), and at the same length of time after initial exposure in all study groups, or it is considered that the outcome assessment methods used would not appreciably bias results. | There is indirect evidence that the study employed appropriate statistical methods. | There is indirect evidence that all measured outcomes that are relevant for the study have been reported, or unplanned analyses are clearly identified, and selective reporting of the outcomes (i.e., only reporting statistically significant results) would not appreciably bias results. | There is indirect evidence that the exposure dose was not administered selectively based on the different cell types or tissues, and a homogeneous cell suspension was obtained. Therefore, each cell had an equal chance to be assigned to any study group, including controls. | There is direct evidence that the exposure precedes the outcome, but an  unreasonable time  between exposure and outcome measurement was  examined. |
| **Probably high RoB (−)** | There is indirect evidence that aerosol or PM exposure was characterized using poorly validated methods, or insufficient information is provided about the validity of the assessment method, but no evidence for concern (NR). | There is indirect evidence that non-treatment-related experimental conditions were not comparable between study groups, or the authors did not report experimental conditions in enough detail (NR). | There is indirect evidence that the outcome assessment methods are unreliable, or the length of time after initial exposure to aerosols or PM differed by study group. | There is indirect evidence that the study did not use appropriate statistical methods. | There is indirect evidence that all measured outcomes that are relevant for the study have not been reported, and/or indirect evidence that unplanned analyses may appreciably bias results, or there is not enough information provided about selective outcome reporting (NR). | There is indirect evidence that exposure dose was administered selectively based on the different cell types, and/or a homogeneous cell suspension was not obtained. Thus, cell number, cell type or administered dose could be different between study groups. | There is indirect evidence that the exposure precedes the outcome, but time between exposure and outcome measurement was unclear and not examined. |
| **Definitely high RoB (−−)** | There is direct evidence that aerosol or PM exposure was characterized using poorly validated methods. | There is direct evidence that control and test cell culture conditions were not comparable. | There is direct evidence that the outcome assessment methods are unreliable, or the length of time after initial exposure to aerosols or PM differed by study group. | There is direct evidence that the study did not use appropriate statistical methods, or did  not use any statistical  methods to  compare control and treated groups. | There is direct evidence that all measured outcomes that are relevant for the study have not been reported, or outcomes are reported using analysis methods that were not pre-specified, or unplanned analyses would appreciably bias results. | There is direct evidence that exposure dose was administered selectively based on the different cell types, and/or a homogeneous cell suspension was not obtained. Thus, cell number, cell type or administered dose could be different between study groups | There is no evidence that the exposure precedes the outcome,  or there is evidence that the outcome can precede the exposure. |

***(Auger et al., 2006) (Tier 1)***

| **RoB criteria** | **RoB assessment** | **Evidence** |
| --- | --- | --- |
| **Can we be confident in the exposure characterization?** | **++** | *«DEP were standard reference materials (SRM) 1650 purchased from the National Institute of Standards and Technology (NIST; Gaitherburg MD, USA) ». «The PM2.5 had been previously physico-chemically characterized (Baulig, 2004) ».* |
| **Were experimental conditions identical across study groups?** | **+** | *«Cells were treated for 24 h on their apical side with 500 μl of medium 1:1 BEBM:DMEM/F12 containing particles in dipalmitoyl lecithin (DPL) (…) DPL at the desired concentration was added to the apical medium of the control cultures. Six Transwell-Clear inserts were used for each type of treatment».* **No evidence of different experimental conditions between groups.** |
| **Can we be confident in the outcome assessment?** | **++** | *«Release of lactate dehydrogenase (LDH), a stable cytosolic enzyme, was performed using the CytoTox 96 kit (Promega, Madison, WI, USA) ».* **Gold standard for cytotoxicity.** *«Concentrations of TNF-α, GM-CSF, IL-6, IL-8 and AR released into the culture medium were measured using enzyme-linked immunosorbent assay (ELISA) kits…».* **Gold standard for cellular inflammation.** *«Intracellular ROS levels were assessed with H2DCF–DA, an oxidation-sensitive fluorescent probe».* **Reliable method for oxidative stress assessment.** |
| **Did the study employ appropriate statistical approaches?** | **++** | *«Means were compared by analysis of variance. The equal variance test is significant with alpha = 0.05 (P < 0.001). All pairwise multiple comparisons were made with the Student–Newman–Keuls method».* |
| **Were all measured outcomes reported?** | **++** | *«Table 1 Effect of DEP on* ***LDH*** *release from ALI cultures of HNE cells». «Table 2 Effect of PM2.5 on LDH release from ALI cultures of HNE cells». «Fig. 2. Effects of PM2.5 on* ***GM-CSF and IL-6*** *secretions». «Fig. 3. Effects of PM2.5 and DEP on* ***amphiregulin and IL-8*** *secretions». «Fig. 5. Effects of particles on* ***intracellular ROS*** *levels».* |
| **Was the administered dose or exposure level adequately randomized?** | **+** | **No evidence of selective administration of PM exposure doses.** |
| **Did the study assess the temporality of exposure and outcome?** | **+** | **Direct evidence that exposure precedes the outcome.** |

***(Barosova et al., 2018) (Tier 1)***

| **RoB criteria** | **RoB assessment** | **Evidence** |
| --- | --- | --- |
| **Can we be confident in the exposure characterization?** | **+** | *«All samples were analysed using SEM (MIRA 3, TESCAN, Czech Republic) with EDS». «Phase analysis of all powder samples was performed by Raman microspectroscopy…». «The brake wear particles’ morphology was investigated via transmission Electron microscopy…». «The concentrations of non-airborne brake wear particles (i.e., samples nLM and NAO) were 2, 1 and 0.5 mg/ mL, considering that majority of the particles sediments on the cells within 24 h and taking into account the effective growth area of the insert (4.2 cm2), the particle deposition corresponds to ~ 48, 24 and 12 µg/cm2, respectively».* **Evidence of different PM characterization methods, but indirect methods for measurement of particle deposition.** |
| **Were experimental conditions identical across study groups?** | **+** | *«After 24 h under submerged conditions the co-cultures were transferred to the ALI conditions, by removing the medium in the upper chamber and replacing the medium in the lower chamber with 1.2 mL of fresh culture medium in 6-well plate or 0.6 mL in 12-well plate, respectively. The cells were then exposed to air for an additional 24 h prior the exposures being performed».* **No evidence of differences between groups for these experimental conditions.** |
| **Can we be confident in the outcome assessment?** | **++** | *«Exposed cells were incubated with propidium iodide (****PI****) (****Annexin-V****-FLUOS staining kit, Roche Diagnostics, Switzerland) to stain necrotic cells for 15 min at RT and then* ***immediately*** ***analysed*** *by flow cytometry».* **Reliable assay for cell viability assessment.** *«The concentration of total glutathione (****GSH****) was determined with a diagnostic glutathione assay kit (…) GSH values are reported relative to the total amount of protein of each sample determined by the Pierce bicinchoninic acid (****BCA****) protein assay kit…».* **Reliable assays for oxidative stress.** *The (pro-)inflammatory response was measured by quantifying the amount of the (pro-)inflammatory mediators (…) via enzyme-linked immunosorbent assay (ELISA)…».* **Gold standard assay for cellular inflammation.** |
| **Did the study employ appropriate statistical approaches?** | **++** | *«Assuming normal distribution of the data sets, a parametric one-way analysis of variance (ANOVA) followed by Dunnett’s multiple comparison test was performed. Results were considered significant if p<0.05».* |
| **Were all measured outcomes reported?** | **++** | *«Fig. 3* ***Cell viability*** *(a) and cellular morphology (b) assessment in lung cell cultures exposed to brake wear particles». «Fig. 4* ***Oxidative stress*** *and* ***(pro-)inflammatory response*** *in lung cells exposed to brake wear particles».* |
| **Was the administered dose or exposure level adequately randomized?** | **+** | *«Co-cultures grown in 6-well plate inserts at the ALI were then exposed to 100 µL suspended non-airborne brake wear particles on the apical side…».* **No evidence of selective administration of particle dose to any specific group.** |
| **Did the study assess the temporality of exposure and outcome?** | **++** | *«However, an increase for PI positive cells for the* ***NAO exposed*** *samples was seen». «A statistically significant increase (p< 0.05) was observed in regards to the release of IL-8* ***following NAO exposure*** *at all concentrations and the positive control». «Exposed cells were incubated with propidium iodide (…) and then* ***immediately analysed*** *by flow cytometry».* **Direct evidence that the exposure precedes the outcome and reasonable time passed between exposure and outcome measurement.** |

***(Bitterle et al., 2006) (Tier 1)***

| **RoB criteria** | **RoB assessment** | **Evidence** |
| --- | --- | --- |
| **Can we be confident in the exposure characterization?** | **++** | *«Special care was taken to provide identical aerosol concentrations to each of these units using a flow distributor that allowed for symmetric flow splitting». «The mass specific surface area of these particles was 750 m^2^g^-1^ as determined by the standard method described by Brunauer, Emmett, and Teller (BET) (Brunauer et al., 1938; Roth et al., 2004) ».* |
| **Were experimental conditions identical across study groups?** | **+** | *«Cells in one chamber were exposed to the aerosol under controlled conditions for 6 h, respectively, while cells in a second chamber were exposed to a control atmosphere».* |
| **Can we be confident in the outcome assessment?** | **++** | *«To test viability of cultured cells the proliferation assay WST-1 (1644807, Roche Diagnostics, Mannheim) was applied».* **Reliable assay for cell viability assessment.** *«To study the impact of changed culture conditions or the effect of ufp on gene expression during cell exposure, transcript levels of IL-6, IL-8 and HO-1 were measured by real-time PCR».* **Reliable assay for gene expression of cellular inflammation and oxidative stress markers.** |
| **Did the study employ appropriate statistical approaches?** | **+** | *«For statistical analysis of the data, we used the Student’s t-test. Results are given as mean values ± SD».* |
| **Were all measured outcomes reported?** | **++** | *«Compared to submersed cultured cell monolayers as control, we found 93.7 ± 9.1%* ***viability*** *after 6 h exposure to clean air and 94.9 ± 9.5% viability after 6 h low, mid and high dose aerosol exposure (n = 13) ». «There was a 2- to 3-fold increase of* ***IL-8*** *and* ***IL-6*** *transcription after 6 h exposure at the ALI with clean air compared to the submersed control». «Furthermore, we found an increased transcription of* ***HO-1*** *in the ALI culture after exposure with clean air compared with submersed cultures».* |
| **Was the administered dose or exposure level adequately randomized?** | **++** | *«Before each exposure experiment all membranes were examined microscopically for confluency of the cell layer, and membranes with inhomogenous cell growth were sorted out».* **Direct evidence that homogeneous cell suspensions were obtained for all study groups.** |
| **Did the study assess the temporality of exposure and outcome?** | **++** | **Direct evidence that the exposure precedes the outcome and reasonable time passed between exposure and outcome measurement.** |

***(Cooney & Hickey, 2011) (Tier 1)***

| **RoB criteria** | **RoB assessment** | **Evidence** |
| --- | --- | --- |
| **Can we be confident in the exposure characterization?** | **++** | *«DEP was quantified by reading the light extinction at 700 nm of DEP suspended in ethanol (…) This method was shown to produce an accurate measure of DEP mass (Cooney and Hickey, 2008) ». «Mass of DEP emitted in each run was determined by multiplying the mass of the suspension lost during actuation by the concentration of DEP in the suspension».* |
| **Were experimental conditions identical across study groups?** | **+** | *«All cells were cultured in an incubator at 37 ^o^C, 85% relative humidity, and 5% CO2. Media was changed every other day and the cells were used in experiments 7–9 days after seeding after they had reached confluence».* **No evidence of different conditions for any study group.** |
| **Can we be confident in the outcome assessment?** | **++** | *«The supernatant was removed and the cells were re-suspended in 100 µl of* ***dichlorofluorescin diacetate****...».* **Reliable assay for oxidative stress assessment.** *«After incubation, cell viability was monitored by* ***MTT*** *assay…». «The amount of* ***LDH*** *was in each of the supernatants, relative to control, was determined by following the instructions of the assay kit».* **Gold standard assays for cell viability, cytotoxicity and cellular inflammation assessments.** |
| **Did the study employ appropriate statistical approaches?** | **++** | *«The statistical significance of the differences in means between groups was determined using the one-way ANOVA when there was a single variable and using two-way ANOVA when there were two variables. Values of p< 0.05 were considered to be statistically significant».* |
| **Were all measured outcomes reported?** | **++** | *«Fig. 6. Fluorescence of Calu-3 cells following 4 h of DEP exposure and 30 min of incubation with* ***dichlorofluorescin*** ***diacetate****…». «Fig. 7. Concentration of interleukin-8 (****IL-8****) in the basolateral media of (a) Calu-3 and (b) A549 cells following 24 h of DEP exposure (mean ± SD, n = 3) ». «Fig. 8. Concentration of granulocyte macrophage-colony stimulating factor (****GM-CSF****) in the basolateral media of Calu-3 cells following 24 h of DEP exposure (mean ± SD, n = 3) ». «Fig. 10. Viability of A549 cells exposed to DEP via the custom deposition device following 24 h, as assessed by* ***MTT*** *assay». «Fig. 11. Lactate dehydrogenase (****LDH****) release to the apical surface of A549 cells…».* |
| **Was the administered dose or exposure level adequately randomized?** | **+** | *«Propellant suspension aerosols were generated with the aim of depositing 10, 50, or 100 µg/cm2 of DEP onto the surface of inserts (groups labelled low, medium, and high exposure, respectively). (…) Untreated control inserts and inserts placed in the CDC without aerosol deposition were also tested».* **No evidence of selective administration of DEP suspensions to any group or cell culture selection bias.** |
| **Did the study assess the temporality of exposure and outcome?** | **++** | *«Cells were* ***incubated for 24 h following treatment****. After incubation, cell viability was monitored by MTT assay (TOX-1 Kit, Sigma) as per the manufacturers’ protocol». «Cells were* ***incubated for 4 h following treatment*** *(…). The supernatant was removed and the cells were re-suspended in 100 µl of dichlorofluorescin diacetate...».* **Direct evidence that exposure precedes the outcomes, and reasonable time between exposure and outcome examined.** |

***(Fizesan et al., 2018) (Tier 1)***

| **RoB criteria** | **RoB assessment** | **Evidence** |
| --- | --- | --- |
| **Can we be confident in the exposure characterization?** | **++** | *«Since DEPM is incompatible with the use of the quartz microbalances due to their light-weighted and electrostatically charged nature, an alternative method based on the approach published by Rudd and Strom (Rudd and Strom, 1981) was used to quantify the deposited particles. In short, the method reported by Rudd and Strom measures the unspecific absorption of DEPM at 750 nm to determine their concentration».* |
| **Were experimental conditions identical across study groups?** | **+** | *«Cells were maintained in a humidified atmosphere with 5% CO2 at 37 °C and tested regularly for Mycoplasma contamination. BD Falcon cell culture inserts (surface area 4.2 cm2; 1 μm pore size; high pore density) were used to obtain the tetra-culture system…». «The seeded cells were left for another 3 days to reach confluence on both side of the semi-permeable membrane. On day 3, the medium was replaced in both compartments».* **No evidence of different conditions for any study group.** |
| **Can we be confident in the outcome assessment?** | **++** | *«Cytotoxicity was assessed using the Lactate Dehydrogenase (****LDH****) detection kit CytoTox 96® Non-Radioactive…». «The concentrations (pg/mL) of IL-6 and IL-8 were determined using Quantikine* ***ELISA*** *assay kits…». «The anti-oxidant response of the cellular model after DEPM exposure was measured based on the release of extracellular heme oxygenase-1 (HMOX-1) using an* ***ELISA*** *kit…». «Real-time quantitative reverse transcription polymerase chain reaction (real-time* ***qRT-PCR****) of pro-inflammatory and stress response markers».* **Gold standard assays for outcome assessment.** |
| **Did the study employ appropriate statistical approaches?** | **++** | *«Statistical comparison was performed by ANOVA, followed by the Tukey-posthoc test on normally distributed data. Significant differences are indicated by asterisks (*) (P < .05) ».* |
| **Were all measured outcomes reported?** | **++** | *«Fig. 4. Extracellular* ***LDH*** *activity in cell-free undernatants (…) after exposure to 1.75, 3.4 and 5 μg/cm2 DEPM». «Fig. 5. The extracellular release of* ***pro-inflammatory cytokines*** ***IL-6*** *(A) and* ***IL-8*** *(B) was analyzed in cell-free undernatants by ELISA at 12 h (black bars) and 24 h (grey bars) post-exposure». «Fig. 6. The extracellular release of the anti-oxidant enzyme* ***HMOX-1*** *at 12 h (black bars) and 24 h (grey bars) after exposure to increasing doses of DEPM». «Table 5* ***Differential gene expression*** *of relevant stress markers in the basolateral compartment of the alveolar model at 12 h and 24 h post-exposure to DEPM…».* |
| **Was the administered dose or exposure level adequately randomized?** | **++** | *«The deposition efficiencies from the three inserts exposed during the same experiment were used to evaluate the intra-exposure variability, while data from three independent experiments were used to evaluate the inter-experiment variability».* **Direct evidence that variability in DEPM deposition between different cell cultures was considered to avoid administration bias.** |
| **Did the study assess the temporality of exposure and outcome?** | **++** | *«Fig. 4. Extracellular LDH activity in cell-free undernatants. The viability was measured* ***at 12 h (black bars) and 24 h (grey bars) after exposure*** *to 1.75, 3.4 and 5 μg/cm2 DEPM». «Fig. 5. The extracellular release of pro-inflammatory cytokines IL-6 (A) and IL-8 (B) was analyzed in cell-free undernatants by ELISA* ***at 12 h (black bars) and 24 h (grey bars) post-exposure****». «Fig. 6. The extracellular release of the anti-oxidant enzyme HMOX-1* ***at 12 h (black bars) and 24 h (grey bars) after exposure*** *to increasing doses of DEPM».* |

***(Gasser et al., 2009) (Tier 1)***

| **RoB criteria** | **RoB assessment** | **Evidence** |
| --- | --- | --- |
| **Can we be confident in the exposure characterization?** | **++** | *«The total number of particles, particle mass and particle surface was measured in realtime during the exposures in the exposure box». «Parallel to the exposure of cell cultures, copper TEM grids were placed in the 6-well plates inside the exposure chamber and exposed to the brake wear particles in 5 additional runs. This allowed to determine the number of particles which were deposited on the cell surface».* |
| **Were experimental conditions identical across study groups?** | **+** | *«…Medium was changed twice weekly. Cells were grown on inserts submersed in medium for 7 d to grow to confluence. Then the medium was removed from the upper chamber while the medium in the lower chamber remained to supply the cells through the membrane…».* **No evidence of differences between study groups regarding these experimental conditions.** |
| **Can we be confident in the outcome assessment?** | **++** | *«In order to determine cell death the release of lactate dehydrogenase (****LDH****) from necrotic cells was measured». «After centrifugation, Interleukin-8 (****IL-8****) was quantified by a commercially available DuoSet* ***ELISA*** *Development kit». «A* ***ROS detection kit*** *was used to detect oxidative stress (Image-iT™ LIVE Green Reactive Oxygen Species Detection Kit, Molecular Probes, Invitrogen AG, Basel, Switzerland). Briefly, intracellular nonspecific ROS were marked with a carboxy-****H2DCFDA*** *solution and the cell nuclei were stained with Hoechst 33342».* **All outcomes measured using reliable assays.** |
| **Did the study employ appropriate statistical approaches?** | **++** | *«To test the influence of the fixed factors exposition and type of braking on the measured variables, a Kruskal-Wallis H-Test was conducted. In case of a significant influence of a fixed factor, a Tamhane 2 Post-Hoc-Test or a Bonferroni t-test (for equal variances) was carried out to compare variables among each other. A Spearman's rank correlation coefficient was calculated to test for correlations between the different factors».* |
| **Were all measured outcomes reported?** | **++** | *«Figure 3* ***Cytotoxicity*** *in exposed A549 cells compared with control cultures». «Figure 4 Production of* ***reactive oxygen species*** *in exposed and nonexposed cells». «Figure 6 Concentrations of* ***IL-8*** *released from A549 cells exposed to different braking behaviours».* |
| **Was the administered dose or exposure level adequately randomized?** | **+** | *«In the present study we have used this system to expose A549 epithelial cells, cultured at the air-liquid interface, to brake wear particles produced by different brake conditions, i.e. "normal deceleration" and "full stop"».* **No evidence of selective administration of exposure dose to any of the groups.** |
| **Did the study assess the temporality of exposure and outcome?** | **+** | *«****Post exposure analysis*** *of the cells included the cell viability and the tight junction (TJ) arrangement, since we have recently shown».* **Direct evidence that exposure precedes the outcome, and all the endpoints were probably measured immediately after exposure.** |

***(Ghio et al., 2013) (Tier 1)***

| **RoB criteria** | **RoB assessment** | **Evidence** |
| --- | --- | --- |
| **Can we be confident in the exposure characterization?** | **++** | *«Coarse PM (PM2.5–10 μm) and fine PM (PM0.1–2.5 μm) were collected onto polyurethane foam (…). Ultrafine particles (PM < 0.1 μm) were collected onto G5300 filters…».* **Reliable particle sampling methods that allow direct characterization of PM.** |
| **Were experimental conditions identical across study groups?** | **+** | **Different exposure agents for each cell type, but no evidence of differences in non-treatment related experimental conditions between study groups.** |
| **Can we be confident in the outcome assessment?** | **++** | *«****Cytotoxicity****, assessed using release of* ***LDH*** *after exposure to particles, was demonstrated to be insignificant». «Relative* ***gene expression*** *in NHBE and BEAS-2B cells was quantified using* ***real-time quantitative PCR****». «****IL-8 and IL-6 concentrations*** *in the cell media were measured using commercially available* ***ELISA*** *kits».* |
| **Did the study employ appropriate statistical approaches?** | **++** | *«Differences between multiple groups were compared using one-way analysis of variance. The post-hoc test employed was Scheffe’s test. Differences between two groups were compared using T tests of independent means. Two-tailed tests of significance were employed. Significance was assumed at P < 0.05».* |
| **Were all measured outcomes reported?** | **++** | *«****Cytotoxicity****, assessed using release of* ***LDH*** *after exposure to particles, was demonstrated to be insignificant». «****IL-8 protein release*** *by cells in hypoxia and normoxia after exposure to the NIST 1648 particle corresponded to RNA changes with cell supernatant concentrations of 654 +/− 79 and 235 +/− 38 pg/mL respectively». «Figure 1* ***Fold change RNA*** *of NHBE cells for IL-8 (A), IL-6 (B), HOX1 (C), and COX2 (D) following exposure to fractions of Chapel Hill ambient air pollution particle».* |
| **Was the administered dose or exposure level adequately randomized?** | **+** | **No evidence of selective administration of exposure dose to any of the groups.** |
| **Did the study assess the temporality of exposure and outcome?** | **++** | *«Particle exposure continued until collection of the specific endpoint. For* ***RT-PCR****, this was* ***4 hours*** *while for* ***cytokines*** *this was* ***24 hours****».* |

***(He et al., 2020) (Tier 1)***

| **RoB criteria** | **RoB assessment** | **Evidence** |
| --- | --- | --- |
| **Can we be confident in the exposure characterization?** | **+** | *«The mass of deposited particles via a microbalance was recorded after exposure to the particle aerosol. The actual applied doses in this study vary between the different samples due to the instrumental uncertainty. Overall, most UFPs samples reach the aimed dose range with the exception of Airport 1 sample, in which the highest dose (0.96 μg /cm2) is much lower compared to the highest dose of the other samples».* |
| **Were experimental conditions identical across study groups?** | **++** | *«…Finally,* ***4 inserts that were not exposed*** *in the cloud system* ***but, with the exception of exposure, treated in the same way*** *including 3 for incubator controls and 1 for measuring the total amount of LDH in cells…».* |
| **Can we be confident in the outcome assessment?** | **++** | *«As an important indicator of barrier function, transepithelial electrical resistance (TEER) of Calu-3 cells was measured by the Evom2 Voltohmmeter with a 4 mm chopstick electrodes». «Cell viability was measured by the* ***MTS*** *assay». «The leakage of total lactate dehydrogenase (LDH) in culture medium was measured with the* ***LDH*** *cytotoxicity detection kit». «To investigate effects of particles on pro-inflammatory responses, the production of pro-inflammatory mediators (IL-6 and IL-8) in basolateral medium was measured using the enzyme-linked immunosorbent assay (****ELISA****) kit».* |
| **Did the study employ appropriate statistical approaches?** | **++** | *«Applying the adverse responses at 5 dose groups, the benchmark dose (BMD) analysis (EFSA, 2009) was performed with PROAST (…) to create a dose–response relationship for each UFPs sample. This analysis has the advantage that it estimates the BMD with a confidence interval, increasing the reliability of outcomes and allowing to compare the toxicity of UFPs samples».* |
| **Were all measured outcomes reported?** | **++** | *«Fig. 3B.* ***TEER*** *changes of Calu-3 cells after 24 h exposure to Airport and Non-Airport UFPs at ALI». «Fig. 4.* ***Cell viability*** *tested by MTS after exposing to UFPs samples for 24 h». «Fig. 5. Relative* ***LDH release*** *of Calu-3 cells after exposing to UFPs samples for 24 h». «Table 2 The* ***concentration of pro-inflammatory mediators*** *(IL-6 and IL-8) in basolateral side medium after exposing to UFPs samples at 5 doses for 24 h».* |
| **Was the administered dose or exposure level adequately randomized?** | **+** | *«The experiment of each sample includes the following 3 steps: First, 3 inserts for exposure controls were exposed with the suspension of a blank filter extraction or re-suspension solution without particles; Second, 4 exposed doses tested in succession for each UFPs sample, with 2 inserts for each dose (duplicate measurement); Finally, 4 inserts that were not exposed in the cloud system but, with the exception of exposure, treated in the same way including 3 for incubator controls and 1 for measuring the total amount of LDH in cells (see 2.6). The mass of deposited particles via a microbalance was recorded after exposure to the particle aerosol».* **Indirect evidence that UFP dose was not administered selectively between the different groups of cell simples.** |
| **Did the study assess the temporality of exposure and outcome?** | **++** | *«Cell viability tested by MTS* ***after exposing to UFPs samples for 24 h****.». «Relative LDH release of Calu-3 cells after exposing to UFPs samples for* ***24 h****». «The concentration of pro-inflammatory mediators (IL-6 and IL-8) in basolateral side medium after exposing to UFPs samples at 5 doses for* ***24 h****».* |

***(Holder et al., 2008) (Tier 1)***

| **RoB criteria** | **RoB assessment** | **Evidence** |
| --- | --- | --- |
| **Can we be confident in the exposure characterization?** | **++** | *«During the ALI exposure, the average chamber temperature and relative humidity were 20ºC and 45%, and the* ***average pollutant concentrations*** *were 7 ppm for NOx, and 0.1% for CO2». «The deposition efficiency and the diesel exhaust size distribution measured during the exposure at the ALI were used to calculate the* ***dose*** *that the cells received».* |
| **Were experimental conditions identical across study groups?** | **++** | *«The immortalized human bronchial epithelial cell line, 16HBE14o (…)* ***was used for all exposures*** *(Cozens et al., 1994). Cells were maintained in the logarithmic phase of growth in collagen-coated flasks in minimum essential medium (MEM) (…) For exposure to the particle suspension, cells were seeded onto 35-mm collagen-coated cell culture dishes at 1.0 x 10^5^ cm2. For ALI exposure, 6- and 24-well plate collagen-coated Transwells…».* **Cells were cultured following the same experimental conditions for both ALI and particle suspension exposure groups.** |
| **Can we be confident in the outcome assessment?** | **++** | *«****Cell viability*** *was assessed using an 3-[4,5-dimethylthiazol-2-yl]-2,5- diphenyl* ***tetrazolium bromide based assay*** *kit, and reported as the optical density (OD) of the solution at 570 nm. The* ***concentration of IL-8*** *released was measured by commercially available* ***ELISA*** *and the measured values are corrected for viability».* |
| **Did the study employ appropriate statistical approaches?** | **+** | *«Values reported as mean and standard error of the mean, n = 3. *Statistically significant compared with control at p < 0.05». «Error bars represent the standard error of the mean, n = 3 for all groups except ALI viability which has n = 4. *Statistically significant compared with baseline at p < 0.05».* Indirect evidence of appropriate statistical methods, but not reported |
| **Were all measured outcomes reported?** | **++** | *«TABLE 2* ***Viability*** *and* ***IL-8 Release*** *for In Vitro Exposure to Suspensions of Diesel Exhaust Particles».*  *«FIG. 5. Comparison of the (a)* ***viability*** *and (b)* ***IL-8 release*** *for ALI and suspension exposure to diesel exhaust particles».* |
| **Was the administered dose or exposure level adequately randomized?** | **+** | *«The dose deposited during the diesel exhaust ALI exposure is estimated from the deposition efficiency calculated from measurements with the test aerosol».* **No evidence of selective administration of the exposure dose, since deposited dose is calculated for each cell insert** |
| **Did the study assess the temporality of exposure and outcome?** | **++** | *«****After ALI and suspension exposures****, cell viability was measured with an 3-[4,5- dimethylthiazol-2-yl]-2,5-diphenyl tetrazolium bromide kit (Sigma, St. Louis MO) and interleukin-8 (IL-8) secretion was measured with an enzyme linked immunosorbent assay (ELISA) kit…».* |

***(Iwanaga et al., 2013) (Tier 1)***

| **RoB criteria** | **RoB assessment** | **Evidence** |
| --- | --- | --- |
| **Can we be confident in the exposure characterization?** | **++** | *«A commercially available UPM preparation (SRM 1648a, National Institute of Standards & Technology, Gaithersburg, MD) was used. (…) UPM was suspended in phosphate-buffered saline (PBS) at a concentration of 1 mg/mL and vortexed for 30 s prior to application».* |
| **Were experimental conditions identical across study groups?** | **+** | **No evidence of different cell culture conditions between exposure groups.** |
| **Can we be confident in the outcome assessment?** | **++** | *«VEGF and TGF-β_2_ protein concentrations in conditioned cell media from the basolateral chamber were measured in duplicate with commercially available* ***ELISA*** *kits». «****Real-time qPCR*** *reactions (…) were run in triplicate using in-house validated primers for MUC5AC (…) and commercially available primers for IL-8».* |
| **Did the study employ appropriate statistical approaches?** | **++** | *«The* ***1-sample t test*** *was used to test the null hypothesis of no change in VEGF or TGF-β_2_ concentrations with UPM exposure (Δ = 0 pg/mL). The* ***2-sample t test*** *with unequal variances was used for group comparisons (asthmatic versus healthy). (…) Statistical significance was set at p< 0.05».* |
| **Were all measured outcomes reported?** | **++** | *«Figure 1. Asthmatic airway epithelial cells have significantly higher baseline* ***concentrations of VEGF*** *(A) but not* ***TGF-β_2_*** *(B) in ALI cultures». «Figure 4.* ***MUC5AC*** *was* ***not differentially expressed*** *in asthmatic AECs compared to healthy cells under PBS only exposure conditions». «Figure 5.* ***IL-8*** *was* ***not differentially expressed*** *in asthmatic AECs compared to healthy cells under unstimulated (PBS only) exposure conditions».* |
| **Was the administered dose or exposure level adequately randomized?** | **+** | *«One hundred microliters of UPM suspension or PBS vehicle control was applied to the apical cell surface and incubated for 96 h in triplicate transwells».* **Indirect evidence of adequate randomization of administered dose to the cell culture replicates.** |
| **Did the study assess the temporality of exposure and outcome?** | **++** | *«Conditioned cell media in the basolateral chamber was sampled immediately* ***prior to exposure and*** ***at 48 and 96 h****».* |

***(Ji et al., 2018) (Tier 1)***

| **RoB criteria** | **RoB assessment** | **Evidence** |
| --- | --- | --- |
| **Can we be confident in the exposure characterization?** | **++** | *«To characterize the DEP, the particles were deposited on glass cover slips using PreciseInhale exposure system (…) The specimens were mounted on an aluminum stub and sputter coated with 10 nm Platinum (Q150T ES, West Sussex, UK) and analyzed in an Ultra 55 field emission scanning electron microscope (SEM) (Zeiss, Oberkochen, Germany) at 5 kV using the secondary electron detector».* |
| **Were experimental conditions identical across study groups?** | **++** | *«The models including only PBEC (PBEC-ALI), models co-cultured with MQ (PBEC-ALI/MQ) and mono-cultures of MQ were then placed inside the exposure modules. Compressed air of 100 bars was used to aerosolize the DEP into the 300 ml holding chamber. The DEP aerosol was then pulled from holding chamber at a main flow rate of 120 ml/min and diverted into triplicate exposure branches at a flow rate of 10 ml/ min. DEP exposures were carried out for 3 mins (…) In the corresponding* ***controls****, sham exposures were performed with* ***identical flow rate settings- and exposure duration*** *using clean air and a clean system.».* |
| **Can we be confident in the outcome assessment?** | **++** | *«The* ***cell viability*** *of both PBEC-ALI and PBEC-ALI/MQ were determined after 24 h (DEP versus sham) using three different methods:* ***Trypan blue assay*** *(…) Lactate dehydrogenase release (****LDH****) assay (…)* ***Annexin V Assay*** *via fluorescence automated cell sorting (FACS) ». «Transcript* ***expression of genes*** *involved in* ***oxidative stress*** *(NFKB, HMOX1, GPx),* ***pro-inflammation*** *(CXCL8, IL6, TNF),* ***tissue injury/repair*** *(MMP9 and TIMP1) (…) were analyzed using the* ***qRT-PCR*** *technique». «Concentrations of* ***IL-6 and CXCL-8*** *in basal medium were measured using the in-house* ***ELISA*** *method described previously».* |
| **Did the study employ appropriate statistical approaches?** | **++** | *«The results are expressed as medians and interquartile ranges (25th-75th percentiles). All the comparisons between groups were performed by Wilcoxon signed rank t test. A p-value< 0.05 was considered as significant».* |
| **Were all measured outcomes reported?** | **++** | *«Fig. 2 Release and mRNA expression of inflammatory biomarkers in models after exposure to diesel exhaust particles (DEP) ». «Fig. 3 mRNA expression of oxidative stress related markers in models after exposure to diesel exhaust particles (DEP) ». «Fig. 4 Release and mRNA expression of extra-cellular markers in models after exposure to diesel exhaust particles (DEP) ».* |
| **Was the administered dose or exposure level adequately randomized?** | **+** | *«After each exposure, DEP were collected from all 3 inserts separately by rinsing them with 200 μL 99% ethanol. The deposited DEP dose in each insert was quantified by measuring the absorbance using spectrophotometric technique».* **Indirect evidence of adequate randomization of administered exposure dose.** |
| **Did the study assess the temporality of exposure and outcome?** | **++** | *«Levels of CXCL-8 (a) and IL-6 (c) secretion in basal medium in PBEC-ALI and PBEC-AL/MQ (N = 9)* ***after exposure to DEP and incubated for 24 h****…». «Fold change of NFKB (a), HMOX1 (b) and GPx (c) expression in PBEC-ALI and PBEC-AL/MQ (N = 6) after exposure to DEP and incubated for 24 h…». «Levels of MMP-9 (a) and TIMP-1 (b) secretion in basal medium in PBEC-ALI and PBEC-AL/MQ (N = 9) after exposure to DEP and incubated for 24 h…».* |

***(Kaur et al., 2022) (Tier 1)***

| **RoB criteria** | **RoB assessment** | **Evidence** |
| --- | --- | --- |
| **Can we be confident in the exposure characterization?** | **++** | *«For* ***pseudo-ALI*** *exposures, (…) A total of 100 μL of the combustion particle suspension (…) with a mass concentration of 252 μg/mL and 84 μg/mL was added to the apical of the inserts to expose the cells to 6 and 2 μg/cm2 of particles, respectively». «For* ***submerged exposures****, the apical media was removed, (…). A total of 500 μL of suspension with mass concentrations 50.4 and 16.8 μg/mL were added to the apical side of the inserts to expose the cells to 6 and 2 μg/cm2 of particles, respectively». «For* ***ALI exposure****, the mass was measured for each deposition run (…) The absorbance value was compared to the calibration curve (…) to obtain the concentration of each suspension (μg/mL). The total mass per area was estimated…».* |
| **Were experimental conditions identical across study groups?** | **+** | *«Particles were generated by combustion of a jet-fuel surrogate at constant combustion conditions. Particles were either deposited directly onto the co-cultures for ALI exposure or collected onto filters for submerged and pseudo-ALI exposures. The co-cultures were grown on standing inserts and evaluated for differences in viability, pro-inflammatory markers, and xenobiotic metabolism».* **No evidence of different non-exposure related experimental conditions between study groups.** |
| **Can we be confident in the outcome assessment?** | **++** | *«The presence of the pro-inflammatory markers (TNFα) was measured using* ***ELISA****…». «The quantitative polymerase chain reaction (qPCR, Applied Biosystems 7900 Real-Time PCR, Thermo Fischer Scientific) was run using RT2 SYBR® Green* ***qPCR*** *master mix (Qiagen, MD, USA) and primers for CYP1A1…». «Cell viability was determined using Cell Counting Kit-8 (****CCK-8****) assay kits (Dojindo Molecular Technologies, Inc., Rockville, MD) according to the manufacturer’s instructions (Fig. A6) ».* |
| **Did the study employ appropriate statistical approaches?** | **++** | *«One-way analysis of variance (ANOVA) with astatsa.com online software and Tukey’s honest significant difference (HSD) post-hoc test was used to compare the difference between the samples mean at significant levels of p < 0.01 and p < 0.05».* |
| **Were all measured outcomes reported?** | **++** | *«For the maximum dose tested, i.e., 6 μg/cm2 for submerged and pseudo-ALI, and 4 μg/cm2 for ALI, the cells exhibited* ***viability*** *of more than 95% (Fig. A6) ». «Exposure to combustion particles caused an increase in the secretion of the pro-inflammatory marker* ***TNFα*** *(Fig. 4) and in* ***CYP1A1*** *gene expression (Fig. 5), with respect to the control, irrespective of the exposure method».* |
| **Was the administered dose or exposure level adequately randomized?** | **+** | *«All experiments were performed thrice, except the ALI exposure for 4 μg/cm2, which was performed twice (due to technical difficulties in ALI chamber). For submerged and pseudo-ALI, 2–3 inserts were exposed for each dose and control. For ALI exposure, 4 inserts were exposed to each dose or control».* **Indirect evidence of adequate randomization of administered dose in all experiments.** |
| **Did the study assess the temporality of exposure and outcome?** | **++** | *«****At 24 hrs post-exposure****, the cells were evaluated for cell viability, secretion of the pro-inflammatory marker (TNFα), and AhR-dependent induction of xenobiotic metabolism (CYP1A1 mRNA gene expression».* |

***(Klein et al., 2017) (Tier 1)***

| **RoB criteria** | **RoB assessment** | **Evidence** |
| --- | --- | --- |
| **Can we be confident in the exposure characterization?** | **++** | *«SEM results confirmed the heterogeneous size distribution with some bigger particles (Fig. 3) and a large proportion of small particles. With increased exposure time, also the likelihood to deposit larger particles was increased. Overall, the distribution of DEPM was uniform for all evaluated doses».* |
| **Were experimental conditions identical across study groups?** | **+** | **No evidence of different experimental conditions between study groups was reported in the article.** |
| **Can we be confident in the outcome assessment?** | **++** | *«The* ***viability*** *of the tetracultures was evaluated at 6, 24 and 48 h after exposure by measuring the* ***conversion of resazurin into resorufin****…». «Second messengers in cell culture undernatants of the tetraculture were analyzed by using the “V-PLEX* ***Human*** ***Cytokine 30-Plex Kit*** *“…». «The methods used in these experiments with EA.hy 926 cells (cell labeling and fixation for confocal microscopy,* ***qRT-PCR****) are the same as the ones described for the tetraculture system».* |
| **Did the study employ appropriate statistical approaches?** | **++** | *«Statistical comparison of the means was performed by ANOVA, followed by the Tukey posthoc test. In figures, significant differences are indicated by asterisks (*) (P < 0.05). In tables, significant differences are indicated by bold letters (P < 0.05) ».* |
| **Were all measured outcomes reported?** | **++** | *«Fig. 5 Impact of the exposure to different amounts of DEPM on the cellular viability of the tetraculture at different time-points after exposure». «After exposure of tetracultures to realistic doses of DEPM, mRNA levels of markers representative for the 3 tiers of the hierarchical oxidative stress response were quantified in endothelial cells (Table 1) ».* |
| **Was the administered dose or exposure level adequately randomized?** | **+** | *«In order to assure a uniform exposure of the cells at the air-liquid-interface (ALI), empty Transwell™ inserts were exposed to DEPM under the same conditions as used for the tetracultures».* **Indirect evidence of adequate randomization of exposure.** |
| **Did the study assess the temporality of exposure and outcome?** | **++** | *«The viability of the tetracultures was evaluated at* ***6, 24 and 48 h after exposure****…». «The endothelial cells of the tetraculture showed a significant increase in the expression of HSP70 mRNA* ***at 24 and 48 h after the indirect exposur****e to 80 ng/cm2 of DEPM (1.36 ± 0.05; 1.27 ± 0.09 fold), compared to the cells analyzed at 6 h after the exposure, which were at the level of the control cells (0.99 ± 0.11 fold) (P < 0.05) (Table 1) ».* |

***(Kunzi et al., 2015) (Tier 1)***

| **RoB criteria** | **RoB assessment** | **Evidence** |
| --- | --- | --- |
| **Can we be confident in the exposure characterization?** | **++** | *«A high-resolution time-of-flight aerosol mass spectrometer (…) delivered highly time- and size-resolved online measurements of particle composition». «To determine mass concentrations from the SMPS measurements, particle density was estimated from the combination of mobility (SMPS) and aerosol mass collected and weighed on Teflon filters».* |
| **Were experimental conditions identical across study groups?** | **+** | **No evidence of different experimental conditions between study groups was reported in the article.** |
| **Can we be confident in the outcome assessment?** | **++** | *«****Cytotoxicity*** *was assessed by apical lactate dehydrogenase (****LDH****) release from the cytosol of damaged cells. Inflammatory responses were assessed by quantifying basolateral release of interleukin (IL)-6, IL-8, monocyte chemotactic protein (MCP)-1 and tumour necrosis factor (TNF)-α». «****Cytokines*** *were analysed using a* ***Bio-Plex bead-based suspension array system*** *and appropriate detection kits…».* |
| **Did the study employ appropriate statistical approaches?** | **++** | *«Results were considered as statistically significant for p< 0.05. Raw data Yraw were replaced with their log-transformed values Y= log10(Yraw) for statistical analyses». «To test whether there is a significant difference between two different cell models across all particle doses Mann-Whitney statistics was performed with stratification for different days of the experiment and different treatments to avoid so-called block effects».* |
| **Were all measured outcomes reported?** | **++** | *«Figure 4. Cellular responses to increasing particle dose. (a)* ***Cytotoxicity*** *measured as fraction of total lactate dehydrogenase (LDH) released from damaged cells into the apical compartment. The* ***inflammatory response*** *was assessed by release of the cytokines (b) interleukin (IL)-6, (c) IL-8 and (d) monocyte chemotactic protein (MCP-1) ».* |
| **Was the administered dose or exposure level adequately randomized?** | **+** | *«We studied the dose-response relationship when gasoline SOA is deposited on normal or diseased airway epithelia as well as BEAS-2B cells». «By varying the enrichment by up to a factor of 30, the dose of deposited particles was varied accordingly allowing study of the dose-response relationship».* **Indirect evidence that the exposure dose was not administered selectively.** |
| **Did the study assess the temporality of exposure and outcome?** | **++** | *«Bio-markers for pulmonary toxicity were assessed* ***24h after aerosol exposure*** *to capture acute cellular responses. Tumour necrosis factor (TNF)-α, a known regulator of local and systemic inflammation, measured* ***6h after aerosol exposure****…».* |

***(Lan et al., 2021) (Tier 1)***

| **RoB criteria** | **RoB assessment** | **Evidence** |
| --- | --- | --- |
| **Can we be confident in the exposure characterization?** | **++** | *«In this study, the growth area-normalized in vitro doses of PM2.5 surrogates tested were 11–45 µg/cm2. We estimated the corresponding concentrations of ambient PM2.5, based on the scenario of a patient with chronic obstructive pulmonary disease (COPD) to conservatively associate exposure of PM2.5 with its toxicity (Table S1) ».* |
| **Were experimental conditions identical across study groups?** | **+** | **No evidence of different experimental conditions between study groups was reported in the article.** |
| **Can we be confident in the outcome assessment?** | **++** | *«****Viability*** *of the cells was assessed using the* ***Trypan Blue Exclusion Assay****». «****Cytokine levels*** *in the supernatants of control and PM2.5-treated S-ALI cells, were quantified using the Multi-Analyte* ***ELISA****rray Kit (Qiagen, Hilden, Germany) ». «The* ***Caspase 3*** ***Colorimetric Assay Kit*** *(Sigma Aldrich) was utilized for determining caspase-3 enzyme activity in S-ALI cells».* |
| **Did the study employ appropriate statistical approaches?** | **++** | *«Cell viability (comprising 4 different groups of data) was analyzed using the One-way ANOVA with post-hoc test (Tukey’s Multiple Comparison Test) in GraphPad Prism 5.0 software. Cytokine array and caspase-3 assay data (comprising two groups per assay) were analyzed with the unpaired t-test. A threshold of statistical significance was set as p < 0.05».* |
| **Were all measured outcomes reported?** | **++** | *«Fig. 2. (D)* ***Cell viability*** *of S-ALI after 24 h treatment at various growth area-normalized doses of PM2.5 surrogates». «Fig. 4. Expression of* ***cytokines*** *in control and S-ALI cells treated with 22 μg/cm2 of PM2.5 surrogates for 24 h». «Fig. 5.* ***Caspase-3*** *enzyme activity in control and S-ALI cells treated with 22 μg/ cm2 of PM2.5 surrogates for 24 h».* |
| **Was the administered dose or exposure level adequately randomized?** | **++** | *«For the same administered dose of PM2.5 surrogates, S-ALI cells exhibited an appreciably less reduction in viability compared to undifferentiated SAEC, which is in accord with the lower particle uptake in S-ALI cells».* **Direct evidence that the exposure dose was not administered selectively based on the different cell types.** |
| **Did the study assess the temporality of exposure and outcome?** | **++** | *«****After 24-hour treatment*** *of the SAECs and S-ALI cells with 0, 11, 22 or 45 μg/cm2 of PM2.5 surrogates in the apical chamber…*  *«Total RNA was isolated from biological triplicates of control S-ALI cells and S-ALI cells treated with 22 µg/cm2 of PM2.5 surrogates* ***after 24 h****, for quantitative transcriptomic analysis».* |

***(Leclercq et al., 2016) (Tier 1)***

| **RoB criteria** | **RoB assessment** | **Evidence** |
| --- | --- | --- |
| **Can we be confident in the exposure characterization?** | **++** | *«Just before cell exposure, air pollution-derived PM4 (i.e., SRM2786) were suspended at concentrations ranging from 2 to 40 mg/mL in sterile HBSS supplemented with amphotericin B (20 mg/mL) at 2% (v/v), and thereafter sonicated for 2 x 1 min».* |
| **Were experimental conditions identical across study groups?** | **+** | *«****Healthy and diseased cells*** *were seeded at 30,000 cells/cm2 on Transwell® polyester permeable membrane cell culture 24 mm inserts in 6-well Transwell® microplate supports (Corning)…». «Thereafter, NHBE and COPD-DHBE cells were exposed at ALI one or three times, for 4 h, at 24 h intervals, as negative* ***controls*** *(i.e., sterile HBSS, 500 mL) or PM4-exposed cells».* **Indirect evidence of identical experimental conditions between study groups.** |
| **Can we be confident in the outcome assessment?** | **++** | *«Twenty-four hours after the last exposure,* ***cytotoxicity*** *in NHBE and COPD-DHBE cells was evaluated through the determination of* ***ATP*** *concentration (CellTiter-Glo® Luminescent Cell Viability, Promega) in cells and extracellular glucose-6-phosphate dehydrogenase (****G6PD****)* ***activity*** *(Vybrant Cytotoxicity Assay Kit, Thermo Fisher Scientific) in cell-free culture media». «****MDA*** *concentration and glutathione status (i.e., GSSG/GSH) were studied in cell lysates using high-performance* ***liquid*** ***chromatography*** *with fluorescence detection…». «OxiSelect™ protein carbonyl* ***ELISA*** *Kit (Cellbiolabs) was used to study* ***protein carbonyl derivatives*** *of Pro, Arg, Lys, Thr after the derivatization of the carbonyl group with dinitrophenylhydrazine (Devos et al., 2013). Oxidative DNA adduct* ***8-OHdG*** *concentration was studied using a commercially available* ***enzyme immunoassay*** *(…) ». «****Cytokine*** *concentrations (i.e., TNF-a, IL-1b, IL-6, IL-8, GM-CSF, and TGF-a) in cell-free culture media were determined using a MILLIPLEX® MAP Human Cytokine/ Chemokine Magnetic Bead Panel-Immunology* ***Multiplex Assay*** *(Merck-Millipore) (Blasco et al., 2016).* ***XME*** ***gene*** ***expression*** *was studied in cell lysates as published elsewhere (Courcot et al., 2012) (…) gene expression relative quantitation was carried out using custom TaqMan TLDA, a 7900HT* ***Real-Time PCR System****».* |
| **Did the study employ appropriate statistical approaches?** | **++** | *«Other toxicological endpoints from NHBE and COPD-DHBE cells exposed to air pollution-derived PM4 were compared with those from negative controls (i.e., sterile HBSS) using the Mann-Whitney U test, with p value correction for multiple comparisons. Statistical analyses were realized with the IBM SPSS Software (v20) (IBM SPSS Software, Inc., USA). Statistically significant differences were reported with p values < 0.05».* |
| **Were all measured outcomes reported?** | **++** | *«Air pollution derived-PM4 exposure induced dose- and time-dependent decreases of* ***ATP concentration*** *in NHBE and COPD-DHBE cells, as shown in Fig. 2». «Moreover, statistically significant releases of* ***G6PD activity*** *were reported only after three exposures to PM4 at its highest concentration (i.e., 20 mg/ cm2; p < 0.01) (Fig. 2) ». «Fig. 3 shows the* ***oxidative endpoints*** *studied in NHBE and COPD-DHBE cells repeatedly exposed to air pollution-derived PM4». «The inflammatory status in NHBE and COPD-DHBE cells repeatedly exposed to air pollution-derived PM4 was evaluated by studying* ***cytokine secretion*** *as shown in Fig. 4». «Table 1 shows the relative* ***XME gene expression*** *in NHBE and COPD-DHBE cells repeatedly exposed to air pollution-derived PM4».* |
| **Was the administered dose or exposure level adequately randomized?** | **+** | *«Thereafter, NHBE and COPD-DHBE cells were exposed at ALI one or three times, for 4 h, at 24 h intervals, as negative controls (i.e., sterile HBSS, 500 mL) or PM4-exposed cells (i.e., concentrations ranging from 1 to 20 mg PM4/cm2, suspended in sterile HBSS, 500 mL) ».* **No evidence of selective administration of PM exposure concentrations.** |
| **Did the study assess the temporality of exposure and outcome?** | **++** | *«****Twenty-four hours after the last exposure****, cytotoxicity in NHBE and COPD-DHBE cells was evaluated through the determination of ATP concentration…». «Fig. 3. Malondialdehyde (MDA), carbonylated protein (protein-CO), 8-hydroxy-20-deoxyguanosine (8-OHdG), total antioxidant status (TAS), superoxide dismutase (SOD), and glutathione status (i.e. glutathione disulfide/reduced glutathione, GSSG/GSH) were evaluated (…)* ***24 h after*** *one or three exposures for 4 h at 24 h intervals to air-pollution-derived PM4 at 5 mg/cm2».* |

***(Leni et al., 2020) (Tier 1)***

| **RoB criteria** | **RoB assessment** | **Evidence** |
| --- | --- | --- |
| **Can we be confident in the exposure characterization?** | **++** | *«PM was determined gravimetrically by weighing the filters before and after exposure at a relative humidity (RH) of 50% and a temperature of 20˚C after conditioning for 48 h». «Filter punches (diameter 10 mm) were taken and weighted to determine the PM concentration before each cell exposure».* |
| **Were experimental conditions identical across study groups?** | **++** | *«Fully differentiated HBE were exposed at the apical cell surface to the water-soluble* ***PM*** *filter extracts* ***for 4 h****. (…) Control cell cultures were either exposed to extracts from field blanks (filters treated the same way apart from exposure to ambient air), or were left untreated in the incubator. As a* ***positive control*** *and to check for the (pro-)inflammatory response capacity, additional cell cultures were exposed to the bacterial endotoxin lipopolysaccharide (****LPS****) from Escherichia coli (Sigma Aldrich, Buchs, Switzerland) at 10 μg/mL in PBS* ***for 4 h****.* **Direct evidence of identical experimental conditions between study groups, including controls.** |
| **Can we be confident in the outcome assessment?** | **++** | *«Induction of* ***cell death*** *was evaluated by measuring the release of cytosolic lactate dehydrogenase (****LDH****) from damaged cells into the apical compartment». «The release of the (pro-)inflammatory mediators interleukin (IL)-6 and IL-8 was assessed in the basolateral compartment collected at 24 h after exposure to PM filter extracts or to the positive control compound LPS, using the* ***Bio-Plex multiplex bead-based suspension array system*** *and the appropriate detection kit…». «We screened 20 genes to evaluate alterations in signaling* ***pathways related to oxidative stress*** *using Gene globe arrays. Gene expression in HBE was examined by isolation of total RNA followed by quantitative real-time polymerase chain reaction (****RT-qPCR****) ». «For* ***OP*** *evaluation, PM was extracted in simulated lung fluid (SLF) at iso-concentration (25 μg/mL) and subsequently analyzed by three different chemical acellular assays (dithiothreitol:* ***DTT****, 2’7’-dichlorofluorescin:* ***DCFH****, ascorbic acid:* ***AA****) ».* |
| **Did the study employ appropriate statistical approaches?** | **++** | *«For cytotoxicity and release of inflammatory mediators, the arithmetic mean values from the triplicate cell cultures of each experiment were compared to the mean values of the untreated control cultures by one-way analysis of variance (ANOVA) followed by Dunnett’s t-test to compare the treated to the control group, or the Bonferroni test for multiple comparisons. To assess the correlation between IL release and OP, metal or inorganic salts composition, non-parametric Spearman correlation (rs) and statistical significance were calculated using the Student’s t-test. A value of p < 0.05 was considered statistically significant».* |
| **Were all measured outcomes reported?** | **++** | *«Fig 1.* ***Cytotoxicity*** *in normal and cystic fibrosis (CF) human bronchial epithelia (HBE) 24 h after exposure to seasonally sampled PM…». «Fig 2.* ***Inflammatory response*** *of normal and cystic fibrosis (CF) human bronchial epithelia (HBE) 24 h after exposure to seasonally sampled PM…». «Fig 3. Screening of* ***gene expression profiles*** *and pathways associated with PM exposure in normal and CF HBE». «The assessment of the* ***OP*** *of PM by the acellular assays dithiothreitol (OPDTT), ascorbic acid (OPAA) and 2’7’-dichlorofluorescin (OPDCFH), shown in Fig 4A and 4C, revealed exposure to PM with a high OP to enhance the release of IL-6…».* |
| **Was the administered dose or exposure level adequately randomized?** | **+** | *«The particle dose deposited on cells was 0.9–2.5 and 8.8–25.4 μg per cm2 of cell culture area for low and high PM doses, respectively (Table 1). The particles were deposited as bolus and left to interact for 4 h with the respiratory epithelium, where antioxidant defense and mucociliary transport replicate the defense mechanisms. In addition, the removal of the particles after 4 h by washing the apical surface mimics the average residence time of particles deposited in this lung compartment».* **No evidence of selective administration of PM doses, since deposited particle dose was estimated for each culture and translated to real exposure conditions by the Multiple Path Particle Dosimetry model** |
| **Did the study assess the temporality of exposure and outcome?** | **++** | *«Induction of cell death was evaluated by measuring the release of cytosolic lactate dehydrogenase (LDH) from damaged cells into the apical compartment. Apical washes were collected* ***4 h and 24 h post exposure*** *and stored at 4˚C until analysis using the colorimetric cytotoxicity detection kitPLUS…». «The release of the (pro-)inflammatory mediators interleukin (IL)-6 and IL-8 was assessed in the basolateral compartment collected* ***at 24 h after exposure*** *to PM filter extracts».* |

***(Leni et al., 2022) (Tier 1)***

| **RoB criteria** | **RoB assessment** | **Evidence** |
| --- | --- | --- |
| **Can we be confident in the exposure characterization?** | **++** | *«For aerosol characterization before cell exposure, the aerosol was split in four parts and delivered to (i) a scanning mobility particle sizer (…), (ii) an aethalometer (...), (iii) a photoacoustic extinctiometer (…), and (iv) a tapered element oscillating microbalance…».* |
| **Were experimental conditions identical across study groups?** | **+** | **No evidence of different experimental conditions between study groups.** |
| **Can we be confident in the outcome assessment?** | **++** | *«****Cytotoxicity*** *was assessed by measuring lactate dehydrogenase (****LDH****) in apical washes, as previously described». «The* ***inflammatory response*** *was evaluated by measuring the release of 102 cytokines and chemokines from cells by high throughput screening using the Proteome profiler* ***Human XL Cytokine Array****…».* |
| **Did the study employ appropriate statistical approaches?** | **++** | *«Mean values were compared using Multiple comparison one-way ANOVA, Bonferroni’s multiple comparison test with **p < 0.005, ***p < 0.0005, and ****p < 0.0001. The data passed the Brown− Forsythe normality test (alpha = 0.05) ».* |
| **Were all measured outcomes reported?** | **++** | *«Figure 2. Cell membrane damage in normal human bronchial epithelia (HBE) at 24 h after exposure to soot particles, either uncoated or coated with SOM from the ozonolysis of α-pinene and oxidation of mesitylene. The release of lactate dehydrogenase (****LDH****) is presented as the percentage of maximal releasable LDH into the apical compartment…». «Figure 4. Screening of* ***cytokines and chemokines*** *involved in cell interaction and adhesion (A, C) and inflammation and immunity (B, D) …».* |
| **Was the administered dose or exposure level adequately randomized?** | **+** | *«The doses used in our study correspond to 1 week of exposure to the following ambient concentrations: (i) 17 μg/m3 of uncoated soot to 114 μg/m3 for c_135 nm, the maximum coating of series no. 1; (ii) 18 μg/m3 of uncoated soot to 61 μg/m3 for c_120 nm, the maximum coating of series no. 2; (iii) 2 μg/m3 of uncoated soot to 16 μg/m3 for c_50 nm, the maximum coating of series no. 3; (iv) 5 μg/m3 of uncoated soot up to 9 μg/m3 for c_50 nm, the maximum coating of series no. 4; and (v) for series no. 5, 106 μg/m3 for SOM_120 and 93 μg/m3 for SOM_116».* **Differences in administered dose of soot particles between groups is due to the different characteristics, but should not bias results.** |
| **Did the study assess the temporality of exposure and outcome?** | **++** | *«****At 24 h after exposure****, the release of lactate dehydrogenase (LDH), indicating cell membrane damage, was measured and proteome analysis, i.e. the release of 102 cytokines and chemokines to assess the inflammatory response, was performed».* |

***(Offer et al., 2022) (Tier 1)***

| **RoB criteria** | **RoB assessment** | **Evidence** |
| --- | --- | --- |
| **Can we be confident in the exposure characterization?** | **++** | *«Four sampling points were used for the online aerosol measurements». «A scanning mobility particle sizer (SMPS) comprised of an electrostatic classifier (TSI; model 3082) connected to a condensation particle counter (TSI; model 3750) was installed…». «In addition, a tapered element oscillating microbalance (TEOM 1400a; Rupprecht & Patashnick Co., Inc.) was used to determine the SP mass concentration at line A».* |
| **Were experimental conditions identical across study groups?** | **++** | *«In addition to the aerosol exposures, each system had a separate clean air (CA; purified compressed laboratory air) exposure sector serving as* ***controls*** *(…) The* ***temperature of each module*** *containing the cells was* ***continuously measured and controlled*** *via external water baths…». «For the mono- and coculture exposure experiments, A549 cells were* ***seeded on the same day*** *on transferrable 24-mm Transwell inserts with a polyester membrane». «For the monoculture, the* ***medium was renewed to ensure equal treatment*** *of both cell culture models».* **Direct evidence of identical experimental conditions between study groups.** |
| **Can we be confident in the outcome assessment?** | **++** | *«For assessing aerosol-induced membrane integrity as a measure of* ***cytotoxicity****, the release of lactate dehydrogenase (****LDH****) was immediately determined from the medium that had been directly collected after exposures by the Cytotoxicity Detection KitPlus». «Malondialdehyde (****MDA****), an indicator of cellular* ***oxidative*** ***stress****, was measured from the frozen collected sample media using the liquid chromatographic (LC) MS/MS method…». «Aliquots of the collected and −80º C-stored exposure media were thawed and analyzed in a 96-well plate, using the enzyme-based immunosorbent assay (****ELISA****; R&D Systems; DY208) for the* ***pro-inflammatory cytokine IL-8****…».* |
| **Did the study employ appropriate statistical approaches?** | **++** | *«Differences between the groups were analyzed using one-way analysis of variance (ANOVA) with the Welch-Satterthwaite approximation for unequal variances (Welch 1947). Multiple comparisons using the Bonferroni correction to control the inflation of type I errors were conducted for all ANOVA results with a p< 0:05 as post hoc analysis for sample comparison».* |
| **Were all measured outcomes reported?** | **++** | *«Figure 4.* ***Cell viability*** *and* ***LDH*** *release after 4-h exposure to SOANAP-SP and SOAβPIN-SP». «Figure 5. Induced* ***oxidative stress and genotoxicity*** *after 4-h exposure to SOANAP-SP and SOAβPIN-SP». «Figure 6.* ***Inflammatory and angiogenic response*** *triggered by SOANAP-SP and SOAβPIN-SP».* |
| **Was the administered dose or exposure level adequately randomized?** | **+** | *«In the present study, the deposition ranged from 0.9 ng/cm2 (1:30 dilution) to 28 ng/cm2 (undiluted) for SOANAP-SP and from 0.6 ng/cm2 (1:30 dilution) to 17 ng/cm2 (undiluted) for SOAβPIN-SP, covering realistic ambient exposure to mild occupational exposure conditions».* **No evidence of selective administration of these aerosol dilutions to any specific cell culture.** |
| **Did the study assess the temporality of exposure and outcome?** | **++** | *«For assessing aerosol-induced membrane integrity as a measure of cytotoxicity,* ***the release of lactate dehydrogenase (LDH) was immediately determined*** *from the medium that had been directly collected after exposures». «****Immediately after the exposure****, inserts were washed twice with PBS and cells were harvested (…) The alkaline version of the comet assay was performed according to the mini-gel comet assay method previously described…».* |

***(Sotty et al., 2019) (Tier 1)***

| **RoB criteria** | **RoB assessment** | **Evidence** |
| --- | --- | --- |
| **Can we be confident in the exposure characterization?** | **++** | *«A high-volume impactor sampler has been set up to collect fine fraction (i.e., PM2.5-0.18, with AED between 2.5 μm and 0.18 μm) by impaction, and quasi-ultrafine fraction (i.e., PM0.18, with AED < 0.18 μm) by filtration on A4 sized polycarbonate filter». «FP and UFP samples were suspended at 46.7 μg/mL in HBSS…». «The rationale for selection of the FP and UFP dose of 5 μg/cm2 in the exposure strategy applied in this work was based on the literature…».* |
| **Were experimental conditions identical across study groups?** | **+** | *«Each cell lot was assigned into 5 groups: control group (i.e., exposed to vehicle solution: HBSS with 1% (v/v) amphotericin-B (250 μg/ mL), FP acute exposure group (i.e., exposed to FP once for 6 h), FP repeated exposure group (i.e., exposed to FP three times for 6 h with 18 h intervals), and similarly, UFP acute exposure group (i.e., exposed to UFP once for 6 h) and UFP repeated exposure group (i.e., exposed to UFP three times for 6 h with 18 h intervals) ».* **Differences in treatment-related experimental conditions, but no differences were reported in cell culture conditions and other non-treatment-related experimental conditions.** |
| **Can we be confident in the outcome assessment?** | **++** | *«****Viability*** *of FP and UFP-exposed NHBE, and asthma- and COPD-DHBE cells was evaluated through the determination of* ***intracellular ATP*** *concentrations (CellTiter-Glo® Luminescent Cell Viability, Promega) and* ***G6PD*** *activities (Vybrant Cytotoxicity Assay Kit, ThermoFisher Scientific) ». «****Concentrations of TNFα, IL-1β IL-6, IL-8, GM-CSF, MCP-1, RANTES, and TGFα*** *have been investigated in cell-free culture media using MILLIPLEX® MAP* ***Human Cytokine/Chemokine*** *Magnetic Bead Panel-Immunology* ***Multiplex Assay****…». «****Gene expression analysis*** *was carried out by using* ***microarrays*** *(…) After the reverse transcription, one color whole Human (039494 slides) 60-mer oligonucleotides 8x60k microarrays (Agilent Technologies) were used to analyze gene expression».* |
| **Did the study employ appropriate statistical approaches?** | **++** | *«A non-parametric test of Kruskal Wallis with a post hoc test of Dunn's for multiple comparison correction has been used to compare the different conditions (i.e., exposed versus control cells, asthma- or COPD-DHBE group versus NHBE group). Results were considered significant when adjusted p value < 0.05».* |
| **Were all measured outcomes reported?** | **++** | *«Fig. 2A and 2B respectively show the* ***intracellular ATP*** *concentrations of NHBE, and asthma- and COPD-DHBE cells after acute or repeated exposures to FP or UFP at 5 μg/cm2». «Fig. 2C and 2D respectively show the lack of any change of* ***G6PD activity*** *in cell-free culture media of NHBE, and asthma- and COPD-DHBE cells acutely or repeatedly exposed to FP or UFP at 5 μg/cm2». «No significant difference between the levels of the* ***inflammatory*** ***mediators*** *secreted in cell-free culture media by the different cell models were reported at their basal states (Fig. 3) ». «Table 3 Top 30 list of the* ***differentially expressed genes*** *in* ***NHBE*** *cells acutely or repeatedly exposed to UFP». «Table 4 Top 30 list of the* ***differentially expressed genes*** *in* ***asthma-DHBE*** *cells acutely or repeatedly exposed to UFP». «Table 5 Top 30 list of the* ***differentially expressed genes*** *in* ***COPD-DHBE cells*** *acutely or repeatedly exposed to UFP».* |
| **Was the administered dose or exposure level adequately randomized?** | **+** | *«The rationale for selection of the FP and UFP* ***dose of 5 μg/cm2*** *in the exposure strategy applied in this work was based on the literature reporting the harmful occurrence of oxidative stress, pro-inflammatory responses, genetic and epigenetic modifications, and/or mitochondrial function alteration in NHBE and/ or COPD-DHBE cells…».* **Same administered dose of FP and UFP for both cell types.** |
| **Did the study assess the temporality of exposure and outcome?** | **++** | *«****After the last exposure****, cell-free culture media were collected and quickly-frozen at −80 °C for the future study of extracellular glucose-6-phosphate dehydrogenase (G6PD) activity and inflammatory mediator secretion…».* |

***(Steiner et al., 2013) (Tier 2)***

| **RoB criteria** | **RoB assessment** | **Evidence** |
| --- | --- | --- |
| **Can we be confident in the exposure characterization?** | **++** | *«Deposited particles were counted by transmission electron microscopy as previously described…». «Furthermore, the concentrations of carbon monoxide (CO), total gaseous hydrocarbons (HC), nitrogen oxides (NOx) and nitrogen monoxide (NO) were measured in the ten-fold diluted exhaust using the Horiba MEXA-9400H exhaust gas measuring system. Concentrations of nitrogen dioxide (NO2) were estimated based on the assumption that NOx is entirely made up of NO and NO2».* |
| **Were experimental conditions identical across study groups?** | **++** | *«The cell cultures were exposed at the air-liquid interface for 2 or 6 h in order to reflect a low and a high dose situation. All cultures were then post-incubated for 6 h at 37 ºC, 5% CO2 and 80% relative humidity. In each experimental repetition, identical cell cultures (produced with immune cells from the same blood donor) were exposed to diluted diesel exhaust or filtered ambient air».* |
| **Can we be confident in the outcome assessment?** | **++** | *«The release of the cytosolic protein lactate dehydrogenase (****LDH****) into the culture medium was used to estimate cell membrane integrity and hence* ***cytotoxicity****». «The total amount of reduced glutathione (****GSH****) in the cell cultures was quantified with a glutathione assay kit (Cayman Chemical) ». «The detected concentrations of reduced GSH are reported relative to the concentrations of total protein in the samples, which was quantified using the Pierce* ***BCA*** *Protein Assay kit (Pierce) ». «****Real-time PCR*** *was performed in the 7500 fast real-time PCR system, (Applied Biosystems) using Fast SYBR Green master mix (Applied Biosystems) as reporter dye. Relative* ***expression levels of*** *hemeoxygenase (****HMOX1****), superoxide-dismutase (****SOD1****), tumor necrosis factor (****TNF****), interleukin-8 (****IL-8****), caspase7 (****CASP7****) and* ***FAS*** *were calculated». «Tumor necrosis factor (TNF)-a and interleukin (IL)-8 in the culture medium were quantified by enzyme-linked immunosorbent assay (****ELISA****) using the human* ***TNF-a DuoSet*** *and the human* ***CXCL8/IL-8 DuoSet****».* |
| **Did the study employ appropriate statistical approaches?** | **−** | *«Statistical evaluation of the results was performed using the procedure described by Cumming and Finch (Cumming and Finch, 2005) ».* **This statistical evaluation procedure is based in the inference by eye confidence intervals, which can be a useful exploratory step in data analysis, but not a substitute for formal statistical methods when precise estimates and confidence intervals are required.** |
| **Were all measured outcomes reported?** | **++** | *«Fig. 1.* ***Cytotoxicity****, pro-apoptotic response and cellular morphology». «Fig. 2.* ***Oxidative stress****. A) levels of total reduced* ***GSH****, B) and C) cellular response to oxidative stress (real-time RT-PCR on* ***HMOX1*** *and* ***SOD1*** *gene expression) ». «Fig. 3.* ***Inflammatory response****. A) And B)* ***gene expression*** *levels of* ***TNF*** *and* ***IL-8*** *(real-time* ***RT-PCR****), C) and D)* ***extracellular*** ***TNF-a*** *and* ***IL-8*** *concentrations (****ELISA****) ».* |
| **Was the administered dose or exposure level adequately randomized?** | **+** | *«The cell cultures were exposed at the air-liquid interface for 2 or 6 h in order to reflect a low and a high dose situation. All cultures were then post-incubated for 6 h at 37 ºC, 5% CO2 and 80% relative humidity».* **No evidence of selective administration of exposure dose to any specific group.** |
| **Did the study assess the temporality of exposure and outcome?** | **++** | *«Upon unfiltered exhaust exposure, we measured the 0.08 ± 0.1-fold (****2 h****) and 0.07 ± 0.09-fold (****6 h****) amount of reduced GSH compared to cell cultures that had been exposed to filtered air. For filtered exhaust exposure, the according values were 0.2 ± 0.07 (****2 h****) and 0.2 ± 0.08 (****6 h****) ».* |

***(Volckens et al., 2009) (Tier 1)***

| **RoB criteria** | **RoB assessment** | **Evidence** |
| --- | --- | --- |
| **Can we be confident in the exposure characterization?** | **++** | *«Upon exiting the concentrator, aerosol was sampled isokinetically onto preweighed, 47 mm Teflon (Teflo 47 mm, Pall Corp., Ann Arbor, MI) filters for gravimetric analysis. Filters were maintained in a temperature and humidity controlled environment for at least 12 h prior to weighing on an analytical microbalance (XP2U, Mettler Toledo, Columbus, OH) ».* |
| **Were experimental conditions identical across study groups?** | **++** | *«Cells from three different donors were exposed to PM (three wells per experiment) with one repetition per donor for a total of six experimental runs. Control cells were exposed to filtered, ambient air with the EAVES chamber running at the* ***same operating conditions as during the exposure tests****».* |
| **Can we be confident in the outcome assessment?** | **++** | *«To monitor* ***cell viability and cytotoxicity****, we measured the release of lactate dehydrogenase (****LDH****) from the cells into the apical compartment». «Levels of mRNA were measured 1 h following the exposure for markers of* ***cellular inflammation*** *(…) Total RNA was isolated from cells using a standard protocol (RNeasy Mini Kit, Qiagen) and quantified by spectrophotometry at 260/280 nm. Transcripts were stored at -80 °C and subsequently converted to cDNA prior to quantification with an Applied Biosystems 7500* ***Real-Time PCR*** *System (TaQMan Cleavage Assay) ».* |
| **Did the study employ appropriate statistical approaches?** | **++** | *«The 3-day cultures show slightly higher levels of LDH release at 30 and 180 min; however, these levels are not substantially higher than basal levels (i.e., incubator controls), nor are they statistically different from the control cultures when* ***compared by t test (p > 0.2)*** *». «These ratios increased to 3.3, 1.8, and 2.4 at 65 µg/cm2, with all exposures being* ***significantly higher*** *than those of the controls* ***(p < 0.01)*** *».* |
| **Were all measured outcomes reported?** | **++** | *«Apical release of* ***LDH*** *is shown in Figure 4 for air-interface cultures placed in the modified EAVES sampling particle-free air for 30 to 180 min». «****Levels of mRNAs*** *coding for inflammatory and oxidatant stress proteins are shown in Figure 5».* |
| **Was the administered dose or exposure level adequately randomized?** | **+** | *«The exposure duration was constant at 3 h per test; however, the deposited mass varied with ambient conditions each day, producing some variation in the dose delivered to cells». «The coefficient of variation in deposited mass between wells was approximately 30%, with slightly more mass collecting down the flow centerline. These differences, however, were not statistically significant among replicate tests».* **Dose delivered to cells differed between cell cultures due to ambient conditions, but such differences are not statistically significant.** |
| **Did the study assess the temporality of exposure and outcome?** | **++** | *«Levels of mRNA were measured* ***1 h following the exposure*** *for markers of cellular inflammation expressed by three genes of interest: heme-oxygenase-1 (HOX-1), cyclooxygenase-2 (COX-2), and interlukin-8 (IL-8) ».* |

***(Despréaux et al., 2023) (Tier 1)***

| **RoB criteria** | **RoB assessment** | **Evidence** |
| --- | --- | --- |
| **Can we be confident in the exposure characterization?** | **+** | *«Epithelia were exposed to PM0.3–2.5 at the ALI by depositing a 10 μL drop of PMInd or PMTraf at concentrations of 45 or 90 μg/cm2 on the apical side, as described by Achard et al. (2019) ».* *«The physicochemical characterization was carried out by studying their size distributions, specific surface areas, organic and inorganic chemicals and ionic species as described by Cazier (Cazier et al., 2016) ».* ***(Suppl. info)*** |
| **Were experimental conditions identical across study groups?** | **++** | *«Exposures were performed* ***twice a week for three consecutive weeks****. Duplicates were considered for each experimental condition and exposure time. Two control groups were considered: Incubator Control, epithelia maintained without exposure in the incubator during one, two, or* ***three*** ***weeks****; Medium Control, epithelia repeatedly exposed to nutrient medium without PM2.5–0.3 during one, two, or* ***three*** ***weeks****».* |
| **Can we be confident in the outcome assessment?** | **++** | *«Once a week, the apical side was washed with nutrient medium to remove mucus and surface desquamating cells, and the tissue integrity was assessed by measuring the transepithelial electrical resistance (****TEER****) using a* ***Voltohmmeter*** *(EVOM2, World Precision Instruments, USA) ». «****Quantitative real-time PCR*** *was used to determine the* ***mRNA transcript profiles*** *of four housekeeping genes - GAPDH, HPRT, ARN18S and RPL13 - to select the most suitable for our experimental conditions (unexposed and exposed epithelia) ».* |
| **Did the study employ appropriate statistical approaches?** | **++** | *«Statistical analyses were performed using R (version 4.1.3, R Core Team (2022)). A Student test under Gaussian assumption was performed to evaluate whether the expressions between two genes differ in exposed and unexposed conditions». «P. adjusted was considered statistically significant when <0.05».* |
| **Were all measured outcomes reported?** | **++** | *«Figure S2* ***TEER*** *values of reconstructed epithelia before exposure (Week 0) and after 1, 2 or 3 weeks (week1, week 2, week 3) of exposure for Medium Control group (10µL drop) and Incubator Control group (no exposure)* *». «Fig. 2. Determination of* ***gene expression modulation*** *after exposure of EpiH to: A. PMInd at 45 μg/cm2 ; B. PMInd at 90 μg/cm2 ; C. PMTraf at 45 μg/cm2 ; D. PMTraf at 90 μg/cm2…».* |
| **Was the administered dose or exposure level adequately randomized?** | **+** | *«Epithelia were exposed to PM0.3–2.5 at the ALI by depositing a 10 μL drop of PMInd or PMTraf at concentrations of 45 or 90 μg/cm2».* **No evidence of selective administration of exposure dose between healthy and asthma epithelia.** |
| **Did the study assess the temporality of exposure and outcome?** | **++** | *«****After epithelia exposures*** *to PMInd and PMTraf, a graph analysis on pairwise expression ratio was considered to assess gene expression modulation for EpiH (Fig. 2) and EpiA (Fig. 3) ». «However, following repeated exposures to PMInd, Ct values were around 25, indicating an increase in expression of this gene following exposure».* |

**Table S3. Study quality/risk of bias criteria for *in vitro* epidemiology studies evaluating the combined effect of urban aerosols and gas pollutants on human airway ALI cultures.**

|  | **Key criteria** | | | **Other quality criteria** | | | |
| --- | --- | --- | --- | --- | --- | --- | --- |
|  | **Can we be confident in the exposure characterization?** | **Were experimental conditions identical across study groups?** | **Can we be confident in the outcome assessment?** | **Did the study employ appropriate statistical approaches?** | **Were all measured outcomes reported?** | **Was the administered dose or exposure level adequately randomized?** | **Did the study assess the temporality of exposure and outcome?** |
| **Definitely low RoB (++)** | There is direct evidence that the exposure was characterized using methods that directly measure aerosol, PM and gas exposure levels, and that exposure was consistently administered with the same method and timeframe across treatment groups. | There is direct evidence that cell culture conditions and other non-treatment-related experimental conditions (e.g., incubator and plate conditions, medium change schedule and washes, solvents used) were identical across study groups, including controls. | There is direct evidence that the outcomes were assessed using well-established methods (the gold standard), and at the same length of time after initial exposure to aerosols, PM and gases in all study groups. | There is direct evidence that the study employed appropriate statistical methods. | There is direct evidence that all measured outcomes that are relevant for the study (outlined in the protocol, methods, abstract, and/or introduction)  have been reported. | There is direct evidence that the exposure dose was not administered selectively based on the different cell types or tissues, and a homogeneous cell suspension was obtained. Therefore, each cell had an equal chance to be assigned to any study group, including controls. | There is direct evidence that the exposure precedes the outcome, and a  reasonable time  between exposure and outcome measurement was  examined. |
| **Probably low RoB (+)** | There is indirect evidence that the exposure was characterized using methods that directly measure aerosol, PM and gas exposure levels, and that exposure was consistently administered with the same method and timeframe across treatment groups. | There is indirect evidence that non-treatment-related experimental conditions were identical across study groups, including controls, or it can be assumed if authors did not report any differences. | There is indirect evidence that the outcomes were assessed using valid and reliable methods (but not the gold standard), and at the same length of time after initial exposure in all study groups, or it is considered that the outcome assessment methods used would not appreciably bias results. | There is indirect evidence that the study employed appropriate statistical methods. | There is indirect evidence that all measured outcomes that are relevant for the study have been reported, or unplanned analyses are clearly identified, and selective reporting of the outcomes (i.e., only reporting statistically significant results) would not appreciably bias results. | There is indirect evidence that the exposure dose was not administered selectively based on the different cell types or tissues, and a homogeneous cell suspension was obtained. Therefore, each cell had an equal chance to be assigned to any study group, including controls. | There is direct evidence that the exposure precedes the outcome, but an  unreasonable time  between exposure and outcome measurement was  examined. |
| **Probably high RoB (−)** | There is indirect evidence that aerosol, PM and/or gas exposure was characterized using poorly validated methods, or insufficient information is provided about the validity of the assessment method, but no evidence for concern (NR). | There is indirect evidence that non-treatment-related experimental conditions were not comparable between study groups, or the authors did not report experimental conditions in enough detail (NR). | There is indirect evidence that the outcome assessment methods are unreliable, or the length of time after initial exposure to aerosols, PM and/or gases differed by study group. | There is indirect evidence that the study did not use appropriate statistical methods. | There is indirect evidence that all measured outcomes that are relevant for the study have not been reported, and/or indirect evidence that unplanned analyses may appreciably bias results, or there is not enough information provided about selective outcome reporting (NR). | There is indirect evidence that exposure dose was administered selectively based on the different cell types, and/or a homogeneous cell suspension was not obtained. Thus, cell number, cell type or administered dose could be different between study groups. | There is indirect evidence that the exposure precedes the outcome, but time between exposure and outcome measurement was unclear and not examined. |
| **Definitely high RoB (−−)** | There is direct evidence that aerosol, PM and/or exposure was characterized using poorly validated methods. | There is direct evidence that control and test cell culture conditions were not comparable. | There is direct evidence that the outcome assessment methods are unreliable, or the length of time after initial exposure to aerosols, PM and/or gases differed by study group. | There is direct evidence that the study did not use appropriate statistical methods, or did  not use any statistical  methods to  compare control and treated groups. | There is direct evidence that all measured outcomes that are relevant for the study have not been reported, or outcomes are reported using analysis methods that were not pre-specified, or unplanned analyses would appreciably bias results. | There is direct evidence that exposure dose was administered selectively based on the different cell types, and/or a homogeneous cell suspension was not obtained. Thus, cell number, cell type or administered dose could be different between study groups | There is no evidence that the exposure precedes the outcome,  or there is evidence that the outcome can precede the exposure. |

***Holder et al., 2007 (Tier 1)***

| **RoB criteria** | **RoB assessment** | **Evidence** |
| --- | --- | --- |
| **Can we be confident in the exposure characterization?** | **++** | *«A TSI scanning mobility particle sizer (SMPS) Model 3071 and TSI condensation particle counter (CPC) Model 3025 were used to measure the particle size distribution within the chamber. A Horiba portable gas analyzer (Model PG-250) was used to measure the concentration of NOx, CO, and SO2 inside the chamber. At the start of an exposure the recirculation pump and diesel generator were turned on and operated for at least 1 h to stabilize the particle concentration within the chamber».* |
| **Were experimental conditions identical across study groups?** | **+** | *«The cells were contained within a sealed enclosure inside an incubator held at 37 ºC. A sample from the environmental chamber was drawn through the enclosure at a constant flow rate, exposing the cells to the diluted exhaust. A control group of transwells was placed in another incubator during the exposure».* **Indirect evidence of identical experimental conditions between study groups.** |
| **Can we be confident in the outcome assessment?** | **++** | *«The response of the cells to the exhaust was quantified by measuring* ***IL-8 secretion*** *into the medium with an enzyme-linked immunosorbent assay (****ELISA****) kit (Biosource).* ***Cell viability*** *was measured with an* ***MTT*** *assay (Sigma) ».* |
| **Did the study employ appropriate statistical approaches?** | **++** | *«Values are reported as mean ± standard error of mean, *P < 0.01 compared to unexposed using* ***student’s t-test****».* |
| **Were all measured outcomes reported?** | **++** | *«Fig. 3.* ***IL-8 secretion*** *for 0, 1, 2, and 4 h exposure to whole diesel exhaust: (a) incubated for 24 h after exposure and (b) incubated from 24 to 48 h after exposure». «Fig. 4. Effect of filtration and denuding of whole diesel exhaust on* ***viability and IL-8 release****».* |
| **Was the administered dose or exposure level adequately randomized?** | **+** | *«As with all types of particle exposure experiments, it is difficult to quantify the dose of particles. In our system, the dose is governed by the particle deposition to the cell surface, which is currently unknown. Quantitative measurements of dose are vital to determine an accurate representation of the particle interaction with the cell».* **Indirect evidence that exposure dose was not administered selectively.** |
| **Did the study assess the temporality of exposure and outcome?** | **++** | *«****Viabilities*** *were measured from a limited sample* ***immediately*** ***after*** *the exposure and after a period of incubation. Measurements of* ***IL-8 secretion*** *were made either* ***immediately*** ***after*** *the exposure to assess IL-8 release during the exposure period* ***or after the indicated incubation time****».* |

***Vaughan et al., 2019 (Tier 1)***

| **RoB criteria** | **RoB assessment** | **Evidence** |
| --- | --- | --- |
| **Can we be confident in the exposure characterization?** | **++** | *«A CAI (California Analytical Instruments) gas analyser was connected before the dilution tunnel to* ***measure the gases*** *in raw exhaust. We used a NDIR (non-dispersive infra-red) CAI 600 CO2 and CO analyser and a CAI 600 CLD (chemiluminescence detector) NOx detector to* ***measure concentrations of CO2, CO and NOx****». «TSI Dustrak (model 8530)* ***measured the mass of particles*** *with a PM2.5 impactor installed. Measured PM2.5 masses were converted into gravimetric mass using the correction factor…».* |
| **Were experimental conditions identical across study groups?** | **++** | *«pHBECs were exposed to diesel, biodiesel or triacetin/biodiesel emissions for* ***30 min*** *through the CULTEX radial flow system (RFS®), which contains three cell exposure chambers allowing simultaneous exposure in triplicate». «Negative control samples were exposed for* ***30 min*** *to ambient HEPA-filtered laboratory air through the CULTEX RFS®».* **Direct evidence of identical experimental conditions between exposure and control groups.** |
| **Can we be confident in the outcome assessment?** | **++** | *«Percentage cell death was measured using* ***cytotoxicity*** *detection kit (Roche, Penzberg) measuring lactate dehydrogenase (****LDH****) activity released from damaged cells into the apical supernatant». «A Water-Soluble Tetrazolium −1 (****WST-1****) cell proliferation assay (Roche Applied Sciences, Penzberg, Germany) was used to measure* ***cellular metabolism****». «Enzyme-linked immunosorbent assays (****ELISA****) were used to measure the* ***IL-8 and IL-6 secretion*** *of HBECs in response to diesel exposure at ALI in the CULTEX RFS® (Hannover, Germany) ». «****qRT-PCR*** *was used to assess the changes in* ***gene expression*** *after diesel exposure.* |
| **Did the study employ appropriate statistical approaches?** | **++** | *«A one-way ANOVA with a Tukey's multiple comparisons post-test was used to assess the effects of the diesel and biodiesel emission exposure on the measured HBEC responses (cell viability, inflammation, oxidative stress and xenobiotic metabolism). Statistical analysis was performed using Prism v6.0c. P values < .05 were considered statistically significant».* |
| **Were all measured outcomes reported?** | **++** | *«Fig. 5. Measurement of* ***cell viability*** *markers after exposure to diesel and triacetin/biodiesel emissions: (a) cellular metabolism, (b) cell death, (c) CASP3 mRNA expression and (d) BCL2 mRNA expression». «Fig. 6.* ***Gene expression*** *of antioxidant,* ***HO-1****, after exposure to diesel, biodiesel and triacetin/biodiesel emissions». «Fig. 7. Measurement of* ***inflammation****:* ***IL-8 and IL-6*** *secretion after exposure to diesel and biodiesel blends». «Fig. 8.* ***Gene expression*** *of xenobiotic metabolism biomarker,* ***CYP1a1****, after exposures to diesel, biodiesel and triacetin/biodiesel emissions».* |
| **Was the administered dose or exposure level adequately randomized?** | **+** | *«In order to maintain relevance to a real-life scenario, the diesel emission dose in this study was 0.79 mg/m3. A higher dose may have increased cellular responses to the diesel emissions, but would have been irrelevant to ambient conditions. The engine parameters used to generate diesel emissions remained consistent when testing the other fuel types».* **Indirect evidence that the exposure concentrations were not administered selectively to any specific exposure group.** |
| **Did the study assess the temporality of exposure and outcome?** | **++** | *«****After completion of exposure****, 250 μL of MEMα with PSG and Fungizone was added to the apical surface of the cell layer and incubated at 37 °C in 5% CO2 for 5 h. This media was then collected and stored at −80 °C as supernatant to test for cell death (LDH assay) and ELISA analysis of IL-8 and IL-6 secretion». «Fig. 6. Gene expression of antioxidant, HO-1, after exposure to diesel, biodiesel and triacetin/biodiesel emissions.* ***Measurements were taken after 30-min exposure*** *to diesel and biodiesel emissions».* |

***Bisig et al., 2018 (Tier 2)***

| **RoB criteria** | **RoB assessment** | **Evidence** |
| --- | --- | --- |
| **Can we be confident in the exposure characterization?** | **+** | *«Data from the air quality measure station during the exposure days were kindly provided by Bernard Sturny, City of Fribourg (…) Data was provided as 30 min averages of PM10 (…), O3 (…), NO2, NO, and NOx (…). Averages over the 12 h where exposures occurred were calculated with Microsoft Excel 2010». «The characterization of the ambient air could include a more detailed analysis (e.g. polycyclic aromatic hydrocarbons, and bioaerosols».* **Reliable data of ambient air quality levels, but exposure dose could not be determined and detailed chemical characterization was not reported.** |
| **Were experimental conditions identical across study groups?** | **+** | *«The exposure system is composed of two exposure chambers (Papaioannou et al., 2006), one to test the aerosol and one as a* ***reference control****, i.e. clean air, to measure possible stress through continuous airstream». «After* ***12 h exposure*** *to ambient or filtered air, the cell cultures were transported back to the laboratory…». «Hydroquinone (Sigma) was used for two genes related to oxidative stress: 100 mM hydroquinone (…) was applied to the basolateral side (1.2 mL) for* ***12 h****». «Phorbol myristate acetate (PMA, Sigma) induced the protein level of IL-1β, a stock solution of 200 mg/mL was diluted in supplemented medium to 40 ng/mL and applied to the basolateral side (1.2 mL) for* ***12 h****».* **Indirect evidence of identical experimental conditions between exposure groups and controls.** |
| **Can we be confident in the outcome assessment?** | **++** | *«The multi-cellular lung model was analyzed for cell morphology and* ***viability*** *using microscopy (cLSM) and* ***LDH-assay****, additionally* ***oxidative stress and pro-inflammation*** *were assessed using* ***gene expression****». «Results of the* ***pro-inflammatory*** *gene expression analysis was confirmed by* ***ELISA****».* |
| **Did the study employ appropriate statistical approaches?** | **++** | *«Significant changes of ambient air LDH levels versus the corresponding control are displayed with #, two-way ANOVA, p < 0.05». «The normalized data are presented as single data points with mean, the Y-axis is plotted in Log2-scale (as data represent fold changes) ». «Statistics was performed with GraphPad Prism using two-way and one-way ANOVAs».* |
| **Were all measured outcomes reported?** | **++** | *«Figure 3. Cell morphology and* ***cytotoxicity*** *assessment». «Figure 4.* ***Gene expression*** *analysis of summer and winter ambient air exposed to the multi-cellular lung model at ALI». «Figure 5. Normalized* ***pro-inflammatory protein levels*** *in the multi-cellular lung model at ALI after winter ambient air exposure».* |
| **Was the administered dose or exposure level adequately randomized?** | **−** | *«Due to the generally very low PN-concentrations in the air, it was not possible to determine the exact dose that reaches the surface of the lung cells».* **Direct evidence that administered dose could be different between study groups.** |
| **Did the study assess the temporality of exposure and outcome?** | **++** | *«****After each day****, cytotoxicity, oxidative stress and pro-inflammatory endpoints were assessed». «****After 12 h exposure*** *to ambient or filtered air, the cell cultures were transported back to the laboratory and cells were* ***either put directly in the incubator*** *for the next day’s exposure* ***or sampled****, meaning that cells and supernatants were collected for further analysis».* |

***Knebel et al., 2002 (Tier 2)***

| **RoB criteria** | **RoB assessment** | **Evidence** |
| --- | --- | --- |
| **Can we be confident in the exposure characterization?** | **++** | *«The analysis of several atmospheric compounds as well as particle concentrations was performed by* ***online monitoring in parallel to the cell exposure****». «For each atmospheric compound, as well as for the particle concentration, mean values over a 5-min measurement period were monitored. Analysis was performed by calculating the mean of 12 measurements per hour and compound».* |
| **Were experimental conditions identical across study groups?** | **++** | *«Incubation intervals and cell preparation for measurement procedures were the same for cells exposed and the air/liquid controls».* |
| **Can we be confident in the outcome assessment?** | **++** | *«****Cell viability*** *measurements were carried out using two different methods. The cleavage of a tetrazolium dye (****WST-1 assay****; Boehringer, Mannheim, Germany) … Aliquots were analysed with an* ***electronic cell counter*** *and analyser system (Schärfe System, Reutlingen, Germany) (Winkelmeier et al., 1993) ».* |
| **Did the study employ appropriate statistical approaches?** | **−−** | **No statistical analysis reported.** |
| **Were all measured outcomes reported?** | **++** | *«The corresponding data of the cell exposure experiments (Fig. 4) showed that undiluted emissions led to similar toxic effects. Independent of the engine operating condition or filtering, a* ***dramatic decrease of cell viability*** *between 44 and 50% of living cells, respectively 22–30% tetrazolium cleavage could be observed». «The particle concentration increased three times when operating the engine under the condition ‘‘higher load’’ (Fig. 5). This exhaust led to a* ***decrease in viability*** *of about 20% for a number of cells, respectively 40% tetrazolium cleavage compared to the control level».* |
| **Was the administered dose or exposure level adequately randomized?** | **+** | **No evidence of selective administration of exposure levels to any exposure group.** |
| **Did the study assess the temporality of exposure and outcome?** | **++** | *«After exposure, the membranes were transferred in conventional companion plates prepared with preconditioned medium (1 ml medium beneath, 0.5 ml medium on top of the membrane) and* ***post-incubated for 2 h prior to further measurements****».* |

***Zarcone et al., 2016 (Tier 1)***

| **RoB criteria** | **RoB assessment** | **Evidence** |
| --- | --- | --- |
| **Can we be confident in the exposure characterization?** | **++** | *«To obtain an extensive characterization of the DE mixture, whole DE was produced twice at 4.5 kW load in a steady-state cycle and collected for analysis. The physical-chemical composition of the mixtures is summarized in Table 2».* |
| **Were experimental conditions identical across study groups?** | **+** | *«The modules were maintained at 37°C with water from a water bath during exposure. As controls, cells in a fourth exposure module were exposed to humidified clean air. As an additional, untreated control, inserts were kept outside the exposure modules and incubated for the same indicated time periods».* **Indirect evidence of identical experimental conditions for all study groups.** |
| **Can we be confident in the outcome assessment?** | **++** | *«Transepithelial Electrical Resistance (TEER) was measured with an electrometer EVOM2…». «****Cytotoxicity*** *(LDH detection Kit, Roche, ver. 10) was assessed by measurement of* ***LDH****». «****qPCRs*** *were performed with a CFX-384 real-time PCR detection system (Bio-Rad) using iQSybr green Supermix (…)* ***Gene expression*** *was expressed as fold from untreated, using the data from cells not included in the Vitrocell unit (incubator controls) ». «****Concentrations of CXCL8*** *in the basal media collected following DE exposure were assessed by an enzyme-linked immunosorbent assay (****ELISA****)… ».* |
| **Did the study employ appropriate statistical approaches?** | **++** | *«The statistical significance of the cellular response cultures from one donor to DE was determined by comparing the means of the triplicate exposures for each condition by one-way ANOVA and Bonferroni posttest to adjust for multiple error testing. In the multiple donor exposures, differences were compared as means of the triplicates of each donor, also by one-way ANOVA-Bonferroni. All statistical analyses were performed with IBM SPSS Statistics Data Editor Version 20. Differences were considered statistically significant at P < 0.05».* |
| **Were all measured outcomes reported?** | **++** | *«Fig. 3. Effect of different exposure durations and dilutions on* ***barrier function****,* ***cytotoxicity****, and* ***CXCL8*** ***protein*** ***release****». «Fig. 4. Effect of different exposure durations on* ***gene expression****».* |
| **Was the administered dose or exposure level adequately randomized?** | **+** | *«For the exposure setup used, deposition efficiencies were calculated for all individual particle size groups. For each particle size group, the calculated deposition efficiency was multiplied with the relative mass concentration of that group, resulting in the fractional efficiency for this particle size group».* **Indirect evidence that dose was not administered selectively to any exposure group.** |
| **Did the study assess the temporality of exposure and outcome?** | **++** | *«Following exposure, cells were removed from the exposure modules, the basal medium was replaced by fresh medium, and incubated for various time periods». «Samples were collected* ***at 6*** *(A, C, and E)* ***and 24 h*** *(B, D, and F) after 60, 150, or 375 min of exposure to air or DE (low, mid, or high) ». «Analysis of mRNA expression was performed* ***at 6 h*** *after 60, 150, or 375 min exposure to air…».* |

***Rossner et al., 2019 (Tier 1)***

| **RoB criteria** | **RoB assessment** | **Evidence** |
| --- | --- | --- |
| **Can we be confident in the exposure characterization?** | **++** | *«The mean concentrations of particulate matter in the diluted exhaust, as determined by* ***gravimetric analysis****, were 0.05 mg/m3. The mean concentration of black soot, as measured by a* ***photo-acoustic analyzer*** *(AVL Microsoot Sensor, AVL List GmbH, Graz, Austria), was 0.02 mg/m3». «A detailed quantitative chemical analysis of PAHs and their derivatives was performed by HPLC with fluorimetric detection».* |
| **Were experimental conditions identical across study groups?** | **++** | *«For the five-day exposure, the* ***cells were first treated identically*** *to the one-day treatment apart from time point T1 in which the cells were not collected».* **Direct evidence of identical experimental conditions between exposure groups.** |
| **Can we be confident in the outcome assessment?** | **++** | *«For* ***TEER*** *measurements, an* ***EVOM2 ohm meter*** *(World Precision Instruments, Sarasota, FL, USA) in combination with an STX2 electrode was used». «****Mucin production*** *is used as a general marker of airway damage. For its analysis, a sandwich enzyme-linked lectin assay (****ELLA****) developed by Epithelix Sàrl (Geneva, Switzerland) was used». «****Lactate dehydrogenase*** *was assessed using the* ***Cytotoxicity*** *Detection Kit (Roche, Basel, Switzerland) and adenylate kinase, using the Adenylate Kinase Cytotoxicity Assay Kit (Abcam, Cambridge, UK) ». «The induction of double-strand DNA breaks was assessed by the detection of serine 139* ***phosphorylation of histone H2AX****. The levels of phosphorylated H2AX (γ-H2AX) were measured (…) using the commercial* ***ELISA*** *kit…». «****mRNA expression*** *was assessed after exposure to complete emissions (…) cDNA was purified again, and a universal* ***PCR*** *was set up with RS-D and FS-D index primers for differentiation of each sample…».* |
| **Did the study employ appropriate statistical approaches?** | **++** | *«The parameters were compared using two-way ANOVA with Sidak’s (post hoc) multiple comparison test and using Student’s t-test (GraphPad Prism version 8 (GraphPad Software Inc., San Diego, CA, USA)). Data were expressed as mean ± standard deviation (SD). Significance values ≤ 0.05 were considered significant».* |
| **Were all measured outcomes reported?** | **++** | *«Figure 1. Transepithelial electrical resistance (****TEER****) measurement in the MucilAir^TM^ tissues». «Figure 2.* ***Mucin production*** *by BEAS-2B cells and the MucilAir^TM^ tissues». «Figure 3. The* ***activity of adenylate kinase*** *after exposure to complete emissions». «Figure 4. The* ***activity of lactate dehydrogenase*** *after exposure to complete emissions». «Figure 5.* ***Histone H2AX phosphorylation*** *after exposure to complete emissions». «Table 1. The* ***expression of genes*** *induced by exposure to complete emissions in the MucilAir^TM^ tissues». «Table 2. The* ***expression of genes*** *induced at time point T5 when compared with T1 in the exposed MucilAir^TM^ tissues».* |
| **Was the administered dose or exposure level adequately randomized?** | **+** | **No evidence of selective administration of exposure dose.** |
| **Did the study assess the temporality of exposure and outcome?** | **++** | *«Once the exposure unit had completed, the cells were transported back to the laboratory, TEER was measured, and the culture medium and cells were collected and stored at −80 ◦C for further analyses (time point T1)* *(Figure 7A). For the five-day exposure, the cells were first treated identically to the one-day treatment apart from time point T1 in which the cells were not collected».* |

***Rossner et al., 2021 (Tier 1)***

| **RoB criteria** | **RoB assessment** | **Evidence** |
| --- | --- | --- |
| **Can we be confident in the exposure characterization?** | **++** | *«The dose of particles deposited on the inserts was estimated based on the gravimetric analysis of PM, the mean mass concentration of particles and particle concentrations, considering particle losses and a deposition rate of 2% as reported in our previous study (Cervena et al., 2020) ».* **Full characterization of PM was reported in the Supplementary data.** |
| **Were experimental conditions identical across study groups?** | **+** | *«In this study, a comparison of 22 lipid peroxidation products and 45 immune response-related molecules assessed at different experimental conditions (two cell models, two exposure approaches) was carried out».* **Different experimental conditions between groups due to the study approach, but this should not bias results.** |
| **Can we be confident in the outcome assessment?** | **++** | *«The analysis was performed by liquid chromatography-tandem mass spectrometry (****LC-MS/MS****)* **(Lipid oxidation)** *». «The* ***production of selected cytokines, chemokines and growth factors*** *into basal tissue culture media was assessed using the* ***Human Cytokine/ Chemokine/Growth Factor 45-Plex ProcartaPlex Panel****».* |
| **Did the study employ appropriate statistical approaches?** | **++** | *«For the multiplex immunoassay, the differences between technical replicates of the exposed and control samples were further evaluated using Student’s t-test; p-values ≤ 0.05 were considered significant. The results identified as biologically significant agreed with those detected as statistically significant by the t-test».* |
| **Were all measured outcomes reported?** | **++** | *«Fig. 1. 1A.* ***Lipid oxidation products*** *detected in culture media collected from MucilAir™ and BEAS-2B cells exposed to complete emissions from E5 and E20 fuels at time points T1 and T5». «Fig. 2. 2A.* ***Production of immune response-relevant molecules*** *by MucilAir™ and BEAS-2B cells exposed to complete emissions from E5 and E20 fuels at time points T1 and T5».* |
| **Was the administered dose or exposure level adequately randomized?** | **+** | **Full characterization of exposure dose was reported in the Supplementary data, and no selective administration was observed for any cell type.** |
| **Did the study assess the temporality of exposure and outcome?** | **++** | *«For the analysis of the parameters further described, a tissue culture medium collected* ***after 1 day (T1) and 5 days (T5)*** *of exposure was used». «****Production of immune response-relevant molecules*** *by MucilAir™ and BEAS-2B cells exposed to EOMs from complete emissions from E5 and E20 fuels* ***at time points T1 and T5****».* |

***Upadhyay et al., 2022 (Tier 1)***

| **RoB criteria** | **RoB assessment** | **Evidence** |
| --- | --- | --- |
| **Can we be confident in the exposure characterization?** | **++** | *«These DEP are well characterized and have been used earlier with bro-ALI models and combined with an aerosolized particle exposure system (XposeALI, 22) ». «The concentrations of the gases were determined based on the air quality index reports from the most polluted cities in Europe, India and China in recent years [2,28]. Based on the literature data on exposure levels, the bro-ALI models were exposed to two different combinations (low and high) of NO_2_ and SO_2_ levels; NO_2_ 0.1 ppm/SO_2_ 0.2 ppm (low doses) or NO_2_ 0.2 ppm/SO_2_ 0.4 ppm (high doses) ».* |
| **Were experimental conditions identical across study groups?** | **++** | *«Sham exposures (exposures with identical flow rate settings using only air and a clean exposure system) were carried out to control for potential viability effects on the model induced by the exposure system».* **Evidence of identical experimental conditions between study groups.** |
| **Can we be confident in the outcome assessment?** | **++** | *«The* ***LDH*** *measurement was analyzed using the Pierce™ LDH* ***Cytotoxicity*** *Assay Kit and performed in accordance with the manufacturer’s instructions». «The* ***IL8 protein levels*** *were determined using the Duoset IL8* ***ELISA*** *KIT (R & D Systems, Minneapolis, MN, US, Catalog # DY208) and the* ***MMP9 protein levels*** *were measured using the MMP9 Duoset* ***ELISA*** *KIT…». «Total RNA extractions and* ***RT-qPCR analysis*** *following the repeated exposures (…) The* ***expression of pro-inflammatory*** *(interleukin: (IL8, IL6) and tumor necrosis factor alpha (TNFα)),* ***oxidative stress*** *(Glutathione S-Transferase Alpha 1 (GSTA1), heme oxygenase 1 (HMOX1) and superoxide dismutase 3 (SOD3))* ***and tissue injury/repair genes*** *(matrix metallopeptidase 9 (MMP9) and tissue inhibitor matrix metalloproteinase 1 (TIMP1)) were quantified».* |
| **Did the study employ appropriate statistical approaches?** | **++** | *«Within each group (sham versus exposure), the comparisons between different exposed groups were assessed by the Friedman test and followed by the Wilcoxon signed rank t test as a post hoc test. In all the tests, p values below 0.05 were considered significant».* |
| **Were all measured outcomes reported?** | **++** | *«Figure 2.****Cytotoxicity*** *following repeated exposure to diesel exhaust particles…». «Figure 3.****Release of IL8*** *in basal medium following repeated exposure to diesel exhaust particles…». «Figure 4.****mRNA expression*** *of inflammatory and oxidative stress markers following repeated exposure to diesel exhaust particles». «Figure 5.****Release of MMP9*** *in basal medium following repeated exposure to diesel exhaust particles».* |
| **Was the administered dose or exposure level adequately randomized?** | **+** | *«Based on data from the study by Ji et al. in 2018 where we tested different doses, we decided to continue with an exposure for 3 minutes, corresponding to an exposure dose of 12.5 µg/cm^2^. The exposure of DEP was carried out for both single and repeated exposures».* **No evidence of selective dose administration to any specific group.** |
| **Did the study assess the temporality of exposure and outcome?** | **++** | *«Both basal medium and cell samples were collected at three different time points as shown in Figure 1, following repeated exposure to DEP, which included the following: exposure 1 (****24 h****), N = 3 donors and n = 2 per donors and, exposure 2 (****48 h****), N = 3 donors and n = 2 per donor’s samples collected, and Exposure 3 (****72 h****), N = 3 donors and n = 2 per donors». «Hence, the protein concentrations measured in this study represent the total secretion of protein in the basal medium* ***24 h after*** *each exposure».* |

***Cervena et al., 2021 (Tier 1)***

| **RoB criteria** | **RoB assessment** | **Evidence** |
| --- | --- | --- |
| **Can we be confident in the exposure characterization?** | **++** | *«Details on particle concentrations and filter loading for diluted and undiluted exhaust are provided in Table 4. The mean concentration of particulate matter in the diluted exhaust, as determined by gravimetric analysis, was 0.175 mg/m3 (0.201 ± 0.089 mg/m3 for cold start tests and 0.148 ± 0.050 mg/m3 for warm start tests). The mean concentration of black soot in undiluted exhaust, as measured by a photo-acoustic analyzer (AVL Microsoot Sensor, AVL List GmbH, Graz, Austria), was 0.3 mg/m3».* |
| **Were experimental conditions identical across study groups?** | **++** | *«Both cell models were cultivated at air-liquid interface at standard conditions (37 ºC, 5% CO2, relative humidity > 90%), and we aimed to keep the* ***same parameters during exposure*** *to emissions to avoid any discrepancy».* |
| **Can we be confident in the outcome assessment?** | **++** | *«****TEER*** *was used as a non-destructive quantitative method for measuring the cell culture integrity and ability to form tight junctions. The measurement was conducted using* ***EVOM2 ohm meter****…». «****Mucin production*** *was quantified in a cell insert apical wash at each timepoint, using sandwich enzyme-linked lectin assay (****ELLA****) developed by Epithelix Sàrl». «For* ***lactate dehydrogenase*** *measurement, the* ***Cytotoxicity*** *Detection Kit (Roche, Basel, Switzerland) was used;* ***adenylate kinase*** *activity was detected using the Adenylate Kinase Cytotoxicity Assay Kit (Abcam, Cambridge, UK) ». «The detection of serine 139* ***phosphorylation of histone H2AX*** *(G-H2AX) was assessed in cell lysates using the* ***ELISA*** *kit…». «The targeted* ***mRNA expression*** *analysis was performed using* ***Human Molecular Toxicology Transcriptome panel*** *(QIAseq Targeted RNA Panel, Qiagen, Hilden, Germany) ». «Selected mRNA expression data from RNA sequencing were verified by quantitative real-time PCR (****qRT-PCR****) ».* |
| **Did the study employ appropriate statistical approaches?** | **++** | *«The parameters were compared using two-way ANOVA with Sidak’s (post hoc) multiple comparison test and using Student’s t-test (GraphPad Prism version 8 (GraphPad Software Inc., San Diego, CA, USA)). Data were expressed as mean ± standard deviation (SD). Significance values ≤ 0.05 were considered significant».* |
| **Were all measured outcomes reported?** | **++** | *«Figure 1. Transepithelial electrical resistance (****TEER****) measurement in the MucilAir™ samples after exposure to complete emissions and control air». «Figure 2. The* ***activity of lactate dehydrogenase*** *after exposure to complete emissions and control air». «Figure 3. The* ***activity of adenylate kinase*** *after exposure to complete emissions or control air». «Figure 4.* ***Mucin production*** *by the MucilAir™ samples and BEAS-2B cells». «Figure 5.* ***Histone H2AX phosphorylation*** *after exposure to complete emissions and control air». «Table 2.* ***The expression of genes*** *induced by exposure to complete emissions in BEAS-2B cells».* |
| **Was the administered dose or exposure level adequately randomized?** | **+** | *«At an assumed 2% deposition rate, about 52.5 ng, or about 50 ng, of particulate matter was deposited in each insert after five-day exposure and about 10 ng after one-day exposure (Table 5) ».* **No evidence of selective administration of PM doses.** |
| **Did the study assess the temporality of exposure and outcome?** | **++** | *«The expression of mRNA was* ***assessed*** *in samples collected at* ***two time points****: after one day and five days of exposure to complete emissions…».* |

***Yu et al., 2017 (Tier 1)***

| **RoB criteria** | **RoB assessment** | **Evidence** |
| --- | --- | --- |
| **Can we be confident in the exposure characterization?** | **++** | *«In order to quantify sizes of particles distributed in ME, we used two aerodynamic particle size spectrometers, TSI-3321 (…) and SMPS-3938 (…) to determine the* ***number, surface area and mass concentration of ME particles*** *in all diameters (TSI Corporation, USA). The* ***CO and CO2 concentrations*** *in the emission were measured using non-dispersive infrared analyzer SM-200 for CO, and GXH-3011 for CO2 (…). The* ***concentration of total volatile organic compounds*** *(TVOCs) was determined using volatile toxic gas analyzer TVA-1000(…). The* ***total hydrocarbon (THC) level*** *was analyzed by gas chromatography GC-2010 (…). The* ***NOx concentration*** *was determined using N-(1-naphthyl)- ethylenediamine dihydrochloride spectrophotometric method as described previously (Yu et al., 2014) ».* |
| **Were experimental conditions identical across study groups?** | **+** | *«The ALI exposure system consisting of three glass medium-containing wells was temperature controlled by circulating 37 ºC water. The ME or clean air was drawn into exposure wells through upper chambers at a flow rate of 25 ml/min/well for 1 h to A549 cells, and 15 ml/min/well for 30, 60, or 90min to BEAS-2B cells».* **Differences in experimental conditions between study groups are considered in the study and should not bias results.** |
| **Can we be confident in the outcome assessment?** | **++** | *«In order to determine the* ***cellular toxicity*** *following ME exposure, both* ***CCK-8 assay*** *(purchased from Dojindo Laboratories, Japan) and* ***LDH released assay*** *(purchased from Beyotime Bioengineering Institute, China) were applied in A549 cells, and CCK-8 assay was used in BEAS-2B cells». «****Intracellular ROS*** *generation was detected by* ***flow cytometry*** *using a peroxide-sensitive fluorescent probe 2’,7’-dichloro-dihydro-fluorescein diacetate (****DCFH-DA****, Nanjing Jiancheng Bioengineering Institute, China) as described by Zhang et al. (2015) with slight modification».* |
| **Did the study employ appropriate statistical approaches?** | **++** | *«Statistical analyses of the differences among three groups were carried by one-way ANOVA with post hoc comparisons by the Tukey test. Data from the time-course study of cell viability in BEAS-2B cell following clean air and fME exposures were analyzed by two-way ANOVA with the post hoc multi-comparisons of the Tukey test. All statistical tests were two-sided with a significant level of p ≤ 0.05».* |
| **Were all measured outcomes reported?** | **++** | *«Table 3* ***Cytotoxicity*** *and the* ***ROS generation*** *of A549 cells following non-fME and fME exposures». «Fig. 4. Time-dependent* ***cytotoxicity*** *of BEAS-2B cells induced by fME using* ***CCK-8 assay****».* |
| **Was the administered dose or exposure level adequately randomized?** | **+** | *«As show in Fig. 1, cells were exposed to either clean air consisted of 21% O2 and 79% N2 (Beijing Oriental Medical Gas Co., Ltd., China); or non-filtered ME (marked as non-filtered ME exposed group, non-fME) and filtered ME using the Acro® 50 Vent Device with a 0.20 mm PTFE Membrane (PN4251, Pall Corporation, USA) (marked as filtered ME exposed group, fME) ».* **No evidence of selective administration of exposure doses to any group.** |
| **Did the study assess the temporality of exposure and outcome?** | **++** | *«To explore the* ***time-dependent cytotoxicity effect****, we exposed BEAS-2B cells to fME and evaluate the cell viability using CCK-8 assay». «Four exposure* ***time points*** *were set to* ***0, 30, 60, and 90min****. Fig. 4 shows no significant difference in CRV percentages after 30min exposure (p = 0.311), but CRV percentages decreases remarkably after 60min and 90min exposure, and was significantly lower than those of the corresponding clean air control groups».* |
